# Five new 2-(2-phenylethyl)chromone derivatives and three new sesquiterpenoids from the heartwood of *Aquilaria sinensis*, an aromatic medicine in China

**DOI:** 10.1007/s13659-022-00326-3

**Published:** 2022-01-28

**Authors:** Lu Zhang, Ping Yi, Hui Yan, Xiao-Nian Li, Meng-Yuan Xia, Jun Yang, Ji-Feng Luo, Yue-Qiu He, Yue-Hu Wang

**Affiliations:** 1 Key Laboratory of Economic Plants and Biotechnology, Yunnan Key Laboratory for Wild Plant Resources, and State Key Laboratory of Phytochemistry and Plant Resources in West China, Kunming Institute of Botany, Chinese Academy of Sciences, Kunming, 650201 People’s Republic of China; 2 Key Laboratory of Chemistry for Natural Products of Guizhou Province, Chinese Academy of Sciences, Guiyang, 550014 People’s Republic of China; 3 Faculty of Plant Protection, Yunnan Agricultural University, Kunming, 650201 People’s Republic of China

**Keywords:** Thymelaeaceae, *Aquilaria sinensis*, Sesquiterpenoids, 2-(2-Phenylethyl)chromones, Neuroprotective

## Abstract

Five new 2-(2-phenylethyl)chromone derivatives, (5*S*,6*R*,7*R*,8*S*,7ʹ*R*)-7ʹ-hydroxyagarotetrol (**1**), (5*S*,6*R*,7*R*,8*S*,7ʹ*S*)-7ʹ-hydroxyagarotetrol (**2**), (6*S*,7*S*,8*R*)-2‑[2‑(4-methoxyphenyl)ethyl]‑6,7,8‑trihydroxy‑5,6,7,8‑tetrahydrochromone (**3**), (6*S*,7*S*,8*R*)-2‑(2-phenylethyl)‑6,7,8‑trihydroxy‑5,6,7,8‑tetrahydrochromone (**4**), (5*S*,6*R*,7*S*,8*R*)-2-(2-phenylethyl)-5,6,7-trihydroxy-5,6,7,8-tetrahydro-8-[2-(2-phenylethyl)-7-methoxychromonyl-6-oxy]chromone (**5**), three new sesquiterpenoids, (4*S*,5*S*,7*S*,8*S*,10*S*,13*R*)-7,8,13-trihydroxyrotunda-1,11-dien-3-one (**6**), (4*S*,5*S*,7*S*,8*S*,10*S*,13*S*)-7,8,13-trihydroxyrotunda-1,11-dien-3-one (**7**), and (4*R*,5*S*,7*S*,8*S*,10*S*,13*S*)-7,8,13-trihydroxyrotunda-1,11-dien-3-one (**8**), along with 14 known compounds were isolated from the resinous heartwood of *Aquilaria sinensis* (Thymelaeaceae). The chemical structures of these new compounds were elucidated by 1D and 2D NMR and MS data, single-crystal X-ray diffraction analysis, and electronic circular dichroism (ECD) calculations. The neuroprotective activities of these isolates were evaluated using an in vitro model of rat adrenal pheochromocytoma (PC12) cell injury induced by corticosterone. At concentrations from 5 to 40 µM, compounds **4** and **6**, agarotetrol (**9**), and 6-hydroxy-2-(2-phenylethyl)chromone (**17**) showed significant protective activities against corticosterone-induced PC12 cell injury (*P* < 0.001).

**Graphical Abstract:**

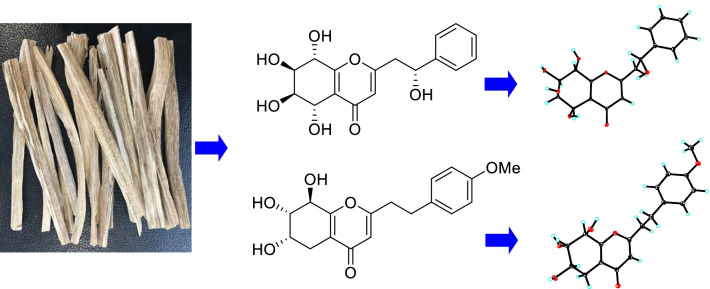

**Supplementary Information:**

The online version contains supplementary material available at 10.1007/s13659-022-00326-3.

## Introduction

**Fig. 1:**
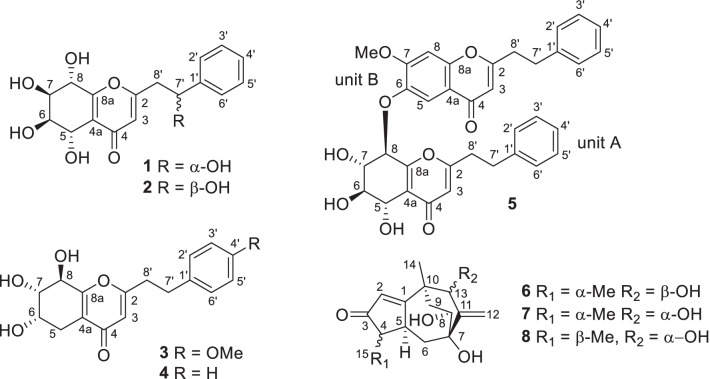
Chemical structures of compounds **1**–**8** from *Aquilaria sinensis*

Chen-xiang (Aquilariae Lignum Resinatum), resinous heartwoods of the Thymelaeaceous plant *Aquilaria sinensis* (Lour.) Spreng., is one of the most well-known aromatic medicines in China [1, 2]. More than 240 compounds, mainly sesquiterpenoids, diterpenoids, steroids, benzyl acetones, chromones, phenolic acids, and aliphatic compounds, have been found in chen-xiang. Some compounds showed antibacterial, anticancer, acetylcholinesterase inhibitory, and other pharmacological activities [3]. Aromatic plants are thought to be a source of chemical constituents with neuroprotective effects [4]. In our continuing efforts to search for neuroprotective compounds from chen-xiang [5, 6], five new 2-(2-phenylethyl)chromone derivatives (**1**–**5**, Fig. 1) and three new sesquiterpenoids (**6**–**8**, Fig. 1), along with 14 known compounds (**9**–**22**, Additional file 1: Fig. S1), were isolated. In the present paper, structural elucidation of these new compounds and bioassay results for the neuroprotective activity of these isolates are reported.

## Results and discussion

### Structure elucidation

Compound **1** was obtained as colorless needles (MeOH). Based on the HRESIMS at *m/z* 357.0942 [M + Na]^+^ (calcd for C_17_H_18_NaO_7_, 357.0950) and ^13^C NMR data (Table 1), its molecular formula was deduced to be C_17_H_18_O_7_ with nine indices of hydrogen deficiency. Its IR spectrum indicated the presence of hydroxy groups (3406 cm^−1^), an *α*,β-unsaturated carbonyl (1658 cm^−1^), and a monosubstituted phenyl ring (1601, 1448, and 701 cm^−1^). According to the ^1^H NMR data of compound **1** (Table 1), a trisubstituted double bond [*δ*_H_ 6.19 (1H, s)] and a monosubstituted phenyl ring [*δ*_H_ 7.26–7.41 (5H, m)] were deduced. Its NMR data (Table 1) were very similar to those of agarotetrol (**9**) [7, 8], a common 2-(2-phenylethyl)-5,6,7,8-tetrahydrochromone found in *Aquilaria* plants. The main difference was that signals for a methylene in agarotetrol were replaced by signals [*δ*_H_ 5.10 (dd, *J* = 8.0, 5.8 Hz); *δ*_C_ 72.4] for an oxygenated methine in compound **1**.

**Table 1:** ^1^H (500 MHz) and ^13^C (126 MHz) NMR data of **1** and** 2** in methanol-*d*_4_ (*δ* in ppm, *J* in Hz)

No	**1**		**2**	
	*δ*_H_	*δ*_C_	*δ*_H_	*δ*_C_
2		169.1		169.2
3	6.19 (s)	115.5	6.22 (s)	115.5
4		181.9		181.9
4a		121.8		121.9
5	4.75 (d, 4.0)	66.7	4.75 (d, 4.0)	66.7
6	4.02 (dd, 4.0, 2.4)	74.0	4.02 (dd, 4.0, 2.3)	74.0
7	4.04 (dd, 7.5, 2.4)	72.4	4.04 (dd, 7.5, 2.3)	72.4
8	4.56 (d, 7.5)	70.1	4.56 (d, 7.5)	70.1
8a		165.4		165.4
1′		144.8		145.0
2′,6′	7.41 (m)	126.9	7.41 (m)	126.9
3′,5′	7.34 (m)	129.5	7.35 (m)	129.5
4′	7.26 (m)	128.8	7.27 (m)	128.8
7′	5.10 (dd, 8.0, 5.8)	72.4	5.11 (dd, 8.0, 5.4)	72.3
8′*α* 8′*β*	3.01 (dd, 14.6, 8.0) 2.97 (dd, 14.6, 5.8)	44.4	3.00 (dd, 14.6, 5.4) 2.96 (dd, 14.6, 8.0)	44.4

**Fig. 2:**
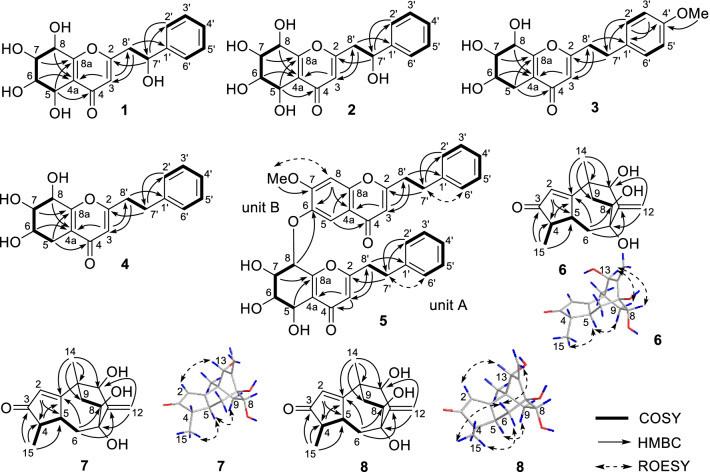
Key 2D NMR correlations of compounds **1**–**8**

Based on the ^1^H‒^1^H COSY correlations of **1** (Fig. 2), fragments of C-5 to C-8, C-2ʹ to C-6ʹ, and C-7ʹ to C-8ʹ were deduced. HMBC correlations (Fig. 2) from *δ*_H_ 7.41 (H-2′, H-6′) to *δ*_C_ 72.3 (C-7′) and from *δ*_H_ 2.97 (H-8′*β*) to *δ*_C_ 144.8 (C-1′) indicated the presence of a 2-hydroxy-2-phenylethyl fragment in **1**. HMBC correlations from *δ*_H_ 2.97 (H-8′*β*) to *δ*_C_ 115.5 (C-3), from *δ*_H_ 6.19 (H-3) to *δ*_C_ 44.4 (C-8′), and from *δ*_H_ 5.11 (H-7ʹ) to *δ*_C_ 169.1 (C-2) implied that the 2-hydroxy-2-phenylethyl fragment was located at C-2. HMBC correlations from *δ*_H_ 4.75 (H-5) to *δ*_C_ 181.9 (C-4) and *δ*_C_ 165.4 (C-8a), from *δ*_H_ 4.56 (H-8) to *δ*_C_ 121.8 (C-4a), and from *δ*_H_ 6.19 (H-3) to *δ*_C_ 121.8 (C-4a), a planar structure 2‑(2‑phenylethyl)‑5,6,7,8,7ʹ‑pentahydroxy‑5,6,7,8‑tetrahydrochromone was deduced.

**Fig. 3:**
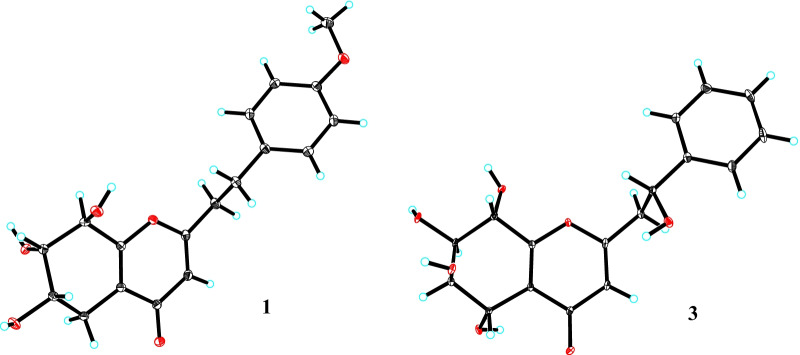
X-ray crystallographic structures of** 1** and **3**

It is difficult to determine the relative configuration of 5,6,7,8-tetrahydroxy-5,6,7,8-tetrahydrochromones by ROESY correlations, while the coupling constants of H-5 to H-8 are helpful. *J*_5,6_ (4.0 Hz), *J*_6,7_ (2.4 Hz), and *J*_7,8_ (7.5 Hz) values of compound **1** were close to those of 5*α*,6*β*,7*β*,8*α*-tetrahydroxy-5,6,7,8-tetrahydrochromone analogs [9], implying the 5*α*,6*β*,7*β*,8*α*-tetrahydroxy configuration in **1**. The relative configuration of H-7ʹ was also determined by comparing its coupling constant with that of known compounds. In isomers (5*S*,6*R*,7*S*,8*R*,7ʹ*R*)-7ʹ-hydroxyisoagarotetrol (**15**) [10] and (5*S*,6*R*,7*S*,8*R*,7′*S*)-7′-hydroxyisoagarotetrol (**16**) [10], the main differences in ^1^H NMR spectra were *J*_7ʹ,8ʹ_ values. Because *J*_7ʹ,8ʹ*α*_ (8.0 Hz) and *J*_7ʹ,8ʹ*β*_ (5.8 Hz) values of compound **1** were close to those of the 7ʹ*α*-OH isomer (**15**; *J*_7ʹ,8ʹ*α*_ = 8.0 Hz and *J*_7ʹ,8ʹ*β*_ = 5.5 Hz), 7ʹ-OH of **1** was deduced to be *α*-oriented. The absolute configuration of **1** was determined to be (5*S*,6*R*,7*R*,8*S*,7ʹ*R*)-7ʹ-hydroxyagarotetrol (Fig. 3) by single-crystal X-ray diffraction using graphite monochromated CuK*α* radiation with a Flack parameter of 0.11 (10).

The molecular formula of **2** was deduced to be the same as that of compound **1**, C_17_H_18_O_7_, by ^13^C NMR (Table 1) and HRESIMS at *m/z* 357.0945 [M + Na]^+^ (calcd for C_17_H_18_NaO_7_, 357.0950). Detailed comparison of its NMR data with those of (5*S*,6*R*,7*S*,8*R*,7ʹ*R*)-7ʹ-hydroxyisoagarotetrol (**1**), (5*S*,6*R*,7*S*,8*R*,7ʹ*R*)-7ʹ-hydroxyisoagarotetrol (**15**), and (5*S*,6*R*,7*S*,8*R*,7′*S*)-7′-hydroxyisoagarotetrol (**16**) [10], compound **2** was elucidated to be 7ʹ-epimer of **1**, which was confirmed by 2D NMR correlations of **2** (Fig. 2). Because *J*_7ʹ,8ʹ*α*_ (5.4 Hz) and *J*_7ʹ,8ʹ*β*_ (8.0 Hz) values of compound **2** were close to those of the 7ʹ*β*-OH isomer (**16**; *J*_7ʹ,8ʹ*α*_ = 5.0 Hz and *J*_7ʹ,8ʹ*β*_ = 8.5 Hz) [10], the 7ʹ-OH of **2** was deduced to be *β*-oriented. Thus, compound **2** was elucidated to be (5*S*,6*R*,7*S*,8*R*,7ʹ*S*)-7ʹ-hydroxyisoagarotetrol.

Compound **3** had the molecular formula C_18_H_20_O_6_ based on ^13^C NMR data (Table 2) and the positive ion at *m*/*z* 355.1150 [M + Na]^+^ (calcd for C_18_H_20_NaO_6_, 355.1158) in the HRESIMS. The ^1^H and ^13^C NMR spectra showed resonances for one *p*-disubstituted phenyl ring [*δ*_H_ 7.12 (2H, br d, *J* = 8.6 Hz) and 6.82 (2H, br d, *J* = 8.6 Hz); *δ*_C_ 159.8, 133.2, 130.4 × 2, and 115.0 × 2], one trisubstituted 4*H*-pyran-4-one [*δ*_H_ 6.07 (s); *δ*_C_ 182.0, 171.2, 163.0, 120.9, and 113.1], one methoxy group [*δ*_H_ 3.75 (3H, s); *δ*_C_ 55.6], three *sp*^3^ methylenes (*δ*_C_ 36.6, 33.0, and 26.5), and three oxygenated methine [*δ*_H_ 4.50 (d, *J* = 5.0 Hz), 4.11 (ddd, *J* = 7.4, 5.1, 2.2 Hz), and 3.90 (dd, *J* = 5.0, 2.2 Hz); *δ*_C_ 75.0, 71.2, and 67.3], implying that it might also be a 5,6,7,8-tetrahydrochromone with the substituted mode of three hydroxy groups at ring B rather than the usual mode of four hydroxy groups at ring B. Four fragments, C-5 to C-8, C-2ʹ to C-3ʹ, C-5ʹ to C-6ʹ, and C-7ʹ to C-8ʹ, were deduced by ^1^H‒^1^H COSY correlations (Fig. 2). Its planar structure was deduced to be 2‑[2‑(4-methoxyphenyl)ethyl]‑6,7,8‑trihydroxy‑5,6,7,8‑tetrahydrochromone by key HMBC correlations (Fig. 2) from H-3 to C-4a and C-8ʹ, from H-5 to C-4 and C-8a, from H-6 and H-8 to C-4a, from H-7 to C-8a, from H-2ʹ and H-6ʹ to C-7ʹ, H-3ʹ and H-5ʹ to C-1ʹ, 4ʹ-OMe to C-4ʹ, H_2_-7ʹ to C-2, C-2ʹ, and C-6ʹ, and H_2_-8ʹ to C-1ʹ and C-3. Finally, the absolute configuration of **3** was determined to be (6*S*,7*S*,8*R*)-2‑[2‑(4-methoxyphenyl)ethyl]‑6,7,8‑trihydroxy‑5,6,7,8‑tetrahydrochromone (Fig. 3) by single-crystal X-ray diffraction using graphite monochromated CuK*α* radiation with a Flack parameter of 0.09 (4).

**Table 2:** ^1^H and ^13^C NMR data of **3** and **4** in methanol-*d*_4_ (*δ* in ppm, *J* in Hz)

No	**3**		**4**	
	*δ*_H_ (600 MHz)	*δ*_C_ (151 MHz)	*δ*_H_ (500 MHz)	*δ*_C_ (126 MHz)
2		171.2		171.1
3	6.07 (s)	113.1	6.09 (s)	113.0
4		182.0		181.9
4a		120.9		120.9
5*β* 5*α*	2.66 (dd, 16.9, 5.1) 2.51 (dd, 16.9, 7.4)	26.5	2.67 (dd, 17.2, 5.0) 2.51 (dd, 17.2, 7.5)	26.5
6	4.11 (ddd, 7.4, 5.1, 2.2)	67.3	4.11 (ddd, 7.5, 5.0, 2.1)	67.3
7	3.90 (dd, 5.0, 2.2)	75.0	3.90 (dd, 5.0, 2.1)	75.0
8	4.50 (d, 5.0)	71.2	4.50 (d, 5.0)	71.2
8a		163.0		163.0
1′		133.2		141.2
2′,6′	7.12 (br d, 8.6)	130.4	7.26 (m)	129.6
3′,5′	6.82 (br d, 8.6)	115.0	7.22 (m)	129.5
4′		159.8	7.18 (m)	127.4
7′	2.96 (m)	33.0	3.02 (m)	33.8
8′	2.89 (m)	36.6	2.92 (m)	36.3
4′-OMe	3.75 (s)	55.6		

**Fig. 4:**
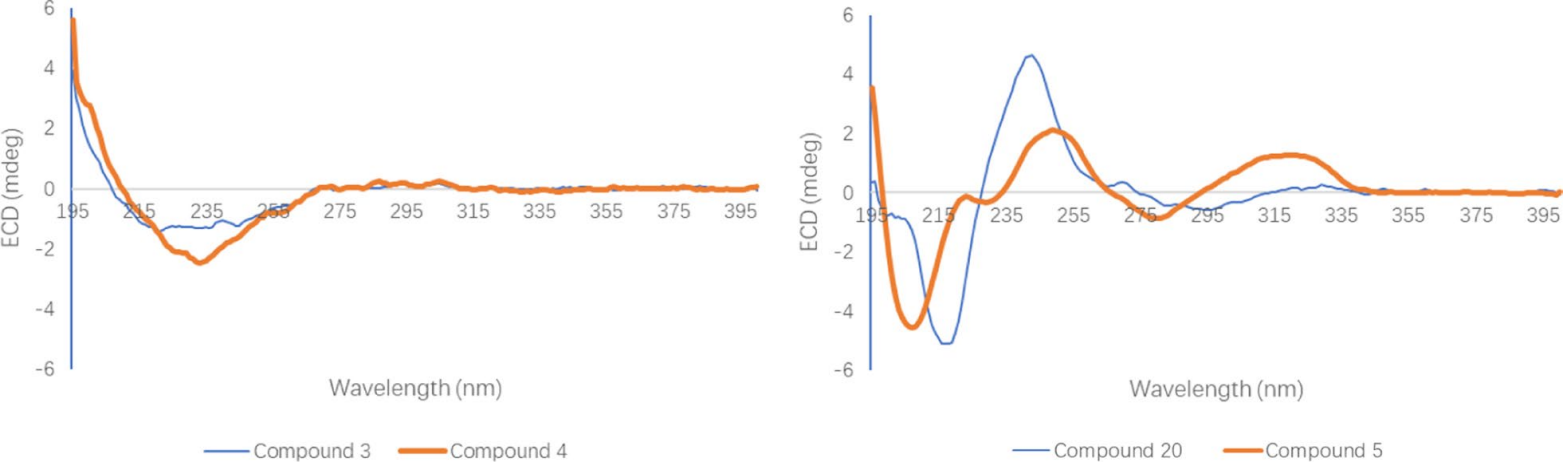
ECD spectra of compounds **3**–**5** and **20**

Compound **4** was assigned the molecular formula C_17_H_18_O_5_ based on ^13^C NMR data (Table 2) and the positive ion at *m*/*z* 325.1046 [M + Na]^+^ (calcd for C_17_H_18_NaO_5_, 325.1052) in the HRESIMS. By extensively comparing the NMR data (Table 2) of compounds **3** and **4**, signals for one monosubstituted phenyl ring were found in **4** rather than the disubstituted phenyl ring in **3**, and signals for a methoxy group disappeared in **4**. Otherwise, the NMR data of these two compounds were very close to each other, implying that compound **4** is the 4ʹ-demethoxy derivative of **3**, which was confirmed by the ^1^H‒^1^H COSY and HMBC correlations of **4** (Fig. 2). The chemical shifts and coupling constants of H-6 [*δ*_H_ 4.11 (ddd, *J* = 7.5, 5.0, 2.1 Hz)], H-7 [*δ*_H_ 3.90 (dd, *J* = 5.0, 2.1 Hz)], and H-8 [*δ*_H_ 4.50 (d, *J* = 5.0 Hz)] of compound **4** were very close to those of H-6 [*δ*_H_ 4.11 (ddd, *J* = 7.4, 5.1, 2.2 Hz)], H-7 [*δ*_H_ 3.90 (dd, *J* = 5.0, 2.2 Hz)], and H-8 [*δ*_H_ 4.50 (d, *J* = 5.0 Hz)] of compound **3**, implying 6*α*-OH, 7*α*-OH, and 8*β*-OH configurations in **4**. The absolute configuration of **4** was suggested to be (6*S*,7*S*,8*R*)-2‑(2-phenylethyl)‑6,7,8‑trihydroxy‑5,6,7,8‑tetrahydrochromone in view of its ECD spectrum similar to that of compound **3** (Fig. 4).

A molecular formula, C_35_H_32_O_9_, was assigned to compound **5** by positive HRESIMS with an ion at *m/z* 619.1939 [M + Na]^+^ (calcd C_35_H_32_NaO_9_, 619.1944) and ^13^C NMR data (Table 3). According to its NMR data (Table 3), signals for two sets of phenylethyl moieties (*δ*_C_ 141.3, 140.9, 129.2 × 2, 129.6 × 2, 129.5 × 4, 127.5, 127.4, 37.0, 36.2, 34.1, and 33.4), one 5,6,7,8-tretrahydroxy-5,6,7,8-tetrahydrochromone moiety [*δ*_H_ 6.13 (s), 5.45 (dd, *J* = 7.3, 1.3 Hz), 4.75 (dd, *J* = 6.9, 1.3 Hz), 4.00 (dd, *J* = 9.6, 7.3 Hz), and 3.83 (dd, *J* = 9.6, 6.9 Hz); *δ*_C_182.0, 171.2, 160.7, 122.5, 114.4, 79.5, 74.9, 73.6, and 70.3], one trisubstituted chromone [*δ*_H_ 7.90 (s), 7.22 (s), and 6.11 (s); *δ*_C_ 179.7, 170.9, 157.3, 155.0, 148.8, 117.4, 110.4, 110.1, and 101.7], and one methoxy group [*δ*_H_ 3.98 (s); *δ*_C_ 57.2], were observed, which implied that this compound might be a dimer of a 2-(2-phenylethyl)chromone and a 5,6,7,8-tretrahydroxy-5,6,7,8-tetrahydro-2-(2-phenylethyl)chromone. In unit A of **5** (Fig. 2), three ^1^H‒^1^H COSY fragments, H-5/H-6/H-7/H-8, H-2ʹ/H-3ʹ/H-4ʹ/H-5ʹ/C-6ʹ, and H_2_-7ʹ/H_2_-8ʹ, along with HMBC correlations from H-3 to C-4a and C-8ʹ, from H-5 and H-7 to C-8a, from H-2ʹ and H-6ʹ to C-7ʹ, from H_2_-7ʹ to C-2, C-2ʹ, and C-6ʹ, and from H_2_-8ʹ to C-1ʹ and C-3, were observed, which implied the presence of a 5,6,7,8-tetrahydroxy-5,6,7,8-tetrahydro-2-(2-phenylethyl)chromone moiety in **5**. In unit B of **5** (Fig. 2), two ^1^H‒^1^H COSY fragments, H-2ʹ/H-3ʹ/H-4ʹ/H-5ʹ/C-6ʹ and H_2_-7ʹ/H_2_-8ʹ, along with HMBC correlations from H-3 to C-4a and C-8ʹ, from H-5 to C-4 and C-8a, from 7-OMe to C-7, from H-8 to C-4a and C-6, from H-2ʹ and H-6ʹ to C-7ʹ, from H_2_-7ʹ to C-2, C-2ʹ, and C-6ʹ, and from H_2_-8ʹ to C-1ʹ and C-3, were observed, which implied the presence of a 6-hydroxy-7-methoxy-2-(2-phenylethyl)chromone moiety in **5**. Units A and B were connected through an ether bond by the HMBC correlation from H-8 of unit A to C-6 of unit B (Fig. 2). The relative configuration of unit A was deduced to be the same as that of a structural analog (5*S*,6*R*,7*S*,8*R*)-2-(2-phenylethyl)-5,6,7-trihydroxy-5,6,7,8-tetrahydro-8-[2-(2-phenylethyl)chromonyl-6-oxy]chromone (**20**) [11] by comparing the coupling constants of H-5 to H-8 (*J*_5,6_ = 6.9 Hz, *J*_6.7_ = 9.6 Hz, and *J*_7.8_ = 7.3 Hz) in **5** with those (*J*_5,6_ = 7.0 Hz, *J*_6.7_ = 9.8 Hz, and *J*_7.8_ = 7.5 Hz) of H-5 to H-8 in the known analog [11]. The absolute configuration of **5** was suggested to be (5*S*,6*R*,7*S*,8*R*)-2-(2-phenylethyl)-5,6,7-trihydroxy-5,6,7,8-tetrahydro-8-[2-(2-phenylethyl)-7-methoxychromonyl-6-oxy]chromone because the ECD spectrum of **5** was similar to that of (5*S*,6*R*,7*S*,8*R*)-2-(2-phenylethyl)-5,6,7-trihydroxy-5,6,7,8-tetrahydro-8-[2-(2-phenylethyl)chromonyl-6-oxy]chromone (**20**) (Fig. 4).

**Table 3:** ^1^H (600 MHz) and ^13^C (151 MHz) NMR data of **5** in methanol-*d*_4_ (*δ* in ppm, *J* in Hz)

No	*δ*_H_	*δ*_C_
Unit A		
2		171.2
3	6.13 (s)	114.4
4		182.0
4a		122.5
5	4.75 (dd, 6.9, 1.3)	70.3
6	3.83 (dd, 9.6, 6.9)	74.9
7	4.00 (dd, 9.6, 7.3)	73.6
8	5.45 (dd, 7.3, 1.3)	79.5
8a		160.7
1′		140.9
2′,6′	6.95 (m)	129.2
3′,5′	7.16 (m)	129.5
4′	7.11 (m)	127.4
7′	2.62 (m)	33.4
8′	2.75 (m) 2.65 (m)	36.2
Unit B		
2		170.9
3	6.11 (s)	110.1
4		179.7
4a		117.4
5	7.90 (s)	110.4
6		148.8
7		157.3
8	7.22 (s)	101.7
8a		155.0
1′		141.3
2′,6′	7.22 (m)	129.5
3′,5′	7.24 (m)	129.6
4′	7.17 (m)	127.5
7′	3.08 (t, 7.5)	34.1
8′	3.02 (t, 7.5)	37.0
7-OMe	3.98 (s)	57.2

**Fig. 5:**
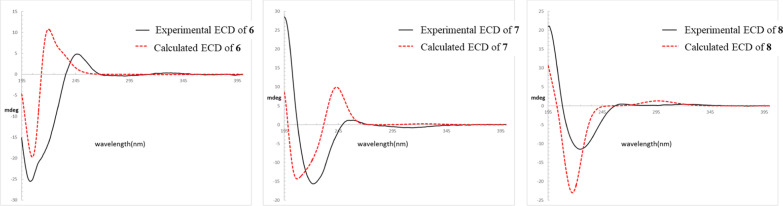
Experimental and calculated ECD spectra of compounds **6–8**

Compound **6** was assigned the molecular formula C_15_H_20_O_4_, as determined by ^13^C NMR data (Table 4) and the positive ion at *m/z* 287.1255 [M + Na]^+^ (calcd for C_15_H_20_NaO_4_, 287.1259) in the HRESIMS. The ^1^H and ^13^C NMR data (Table 4) indicated the presence of one *α*,*β*-unsaturated ketone [*δ*_H_ 5.91 (br s); *δ*_C_ 214.1, 188.3, and 130.4], one exocyclic double bond [*δ*_H_ 5.55 (br s) and 5.36 (br s); *δ*_C_ 156.3 and 112.3], two methyl groups [*δ*_H_ 1.29 (s) and 1.14 (d, *J* = 7.4 Hz); *δ*_C_ 25.2 and 15.4], one methylene, four methines including two oxygenated groups [*δ*_H_ 4.12 (t, *J* = 2.2 Hz) and 3.88 (dd, *J* = 10.7, 5.3 Hz); *δ*_C_ 75.4 and 71.8], and two quaternary carbon atoms (*δ*_C_ 77.3 and 42.8). Its NMR data were similar to those of (4*R*,5*S*,7*R*,8*S*,10*S*,13*R*)-8,13-dihydroxyrotunda-1,11-dien-3-one with a rare tricyclic rotundane skeleton [5]. According to the ^1^H‒^1^H COSY correlations (Fig. 2), two fragments, C-15-C-4-C-5-C-6 and C-8-C-9, were deduced. According to HMBC correlations (Fig. 2) from H-2 to C-4 and C-5, from H_3_-15 to C-3, C-4, and C-5, from H_2_-6 to C-1, C-8, and C-11, from H_2_-12 to C-7 and C-13, and from H_3_-14 to C-1, C-9, C-10, and C-13, the planar structure of **6** was elucidated to be 7,8,13-trihydroxyrotunda-1,11-dien-3-one. The ROESY correlations (Fig. 2) of H_3_-15/H-5 and H-5/H-9*α* indicated that these protons should be cofacial; the ROESY correlations of H-9*β*/H-13 and H-8/H-13 indicated that these protons should also be cofacial. Thus, the relative configuration of **6** was determined, as shown in Fig. 2. By comparing its experimental and calculated ECD spectra (Fig. 5), the structure of compound **6** was finally elucidated to be (4*S*,5*S*,7*S*,8*S*,10*S*,13*R*)-7,8,13-trihydroxyrotunda-1,11-dien-3-one.

**Table 4:** ^1^H and ^13^C NMR data of **6**–**8** in methanol-*d*_4_ (*δ* in ppm, *J* in Hz)

No	**6**		**7**		**8**	
	*δ*_H_^a^	*δ*_C_^b^	*δ*_H_^c^	*δ*_C_^d^	*δ*_H_^c^	*δ*_C_^d^
1		188.3		191.5		191.7
2	5.91 (br s)	130.4	5.95 (d, 1.6)	128.5	5.88 (d, 1.4)	128.5
3		214.1		214.1		214.5
4	1.87 (m)	51.1	1.89 (qd, 7.5, 2.1)	51.5	2.60 (m)	45.5
5	2.98 (m)	47.0	3.02 (m)	47.3	3.62 (m)	41.6
6*α* 6*β*	2.75 (dd, 12.5, 8.9) 1.35 (t, 12.5)	45.9	2.83 (dd, 12.3, 9.3) 1.26 (ddd, 12.3, 10.2, 1.2)	46.8	2.46 (dd, 12.1, 8.8) 1.22 (ddd, 12.1, 11.1, 1.1)	43.2
7		77.3		77.1		77.4
8	3.88 (dd, 10.7, 5.3)	71.8	4.11 (ddd, 10.5, 6.0, 1.2)	71.7	4.09 (ddd, 10.6, 5.6, 0.9)	71.8
9*α* 9*β*	1.88 (m) 2.05 (dd, 15.0, 10.7)	41.5	1.73 (ddd, 14.5, 6.0, 1.0) 2.31 (dd, 14.5, 10.5)	38.6	1.81 (ddd, 14.5, 5.6, 0.9) 2.33 (dd, 14.5, 10.7)	38.4
10		44.8		43.7		43.6
11		156.3		156.3		156.3
12	5.55 (br s) 5.36 (br s)	112.3	5.54 (d, 0.8) 5.19 (br s)	116.5	5.53 (s) 5.17 (s)	116.5
13	4.12 (t, 2.2)	75.4	3.88 (s)	78.5	3.89 (br s)	78.6
14	1.29 (s)	25.2	1.30 (s)	24.5	1.29 (s)	24.5
15	1.14 (d, 7.4)	15.4	1.15 (d, 7.5)	15.9	1.02 (d, 7.6)	10.5

^a^Measured at 500 MHz; ^b^measured at 126 MHz; ^c^measured at 800 MHz; ^d^measured at 201 MHz

Compound **7** has the molecular formula C_15_H_20_O_4_ according to its ^13^C NMR data (Table 4) and HRESIMS at 287.1253 [M + Na]^+^ (calcd for C_15_H_20_NaO_4_, 287.1259). Its ^13^C NMR data exhibited 15 signals for one *α*,*β*-unsaturated ketone functionality (*δ*_C_ 214.1, 191.5, and 128.5), one exocyclic double bond (*δ*_C_ 156.3 and 116.5), two quaternary carbon atoms (*δ*_C_ 77.1 and 43.7), one methine, four methylenes including two oxygenated groups (*δ*_C_ 78.5 and 71.7), and two methyl groups (*δ*_C_ 24.5 and 15.9). The NMR data of compounds **6** and **7** were very close to each other, and both of these compounds have the same molecular formula, which implied that compound **7** might also be a rotundane-type sesquiterpenoid.

The ^1^H‒^1^H COSY fragments H_3_-15/H-4/H-5/H_2_-6 and H-8/H_2_-9 were determined from the ^1^H‒^1^H COSY correlations of **7** (Fig. 2). Based on the HMBC correlations (Fig. 2) from H-2 to C-4 and C-5, from H_3_-15 to C-3, C-4, and C-5, from H_2_-6 to C-1, C-8, and C-11, from H_2_-12 to C-7 and C-13, and from H_3_-14 to C-1, C-9, C-10, and C-13, the planar structure of **7** was elucidated to be the same as that of compound **6**, namely, 7,8,13-trihydroxyrotunda-1,11-dien-3-one. Because *J*_8,9*α*_ (6.0 Hz) and *J*_8,9*β*_ (10.5 Hz) values in the ^1^H NMR data of compound **7** were similar to those (*J*_8,9*α*_ = 5.3 Hz and *J*_8,9*β*_ = 10.8 Hz) of compound **6**, H-8 in compound **7** was elucidated to be *β*-oriented. Correlations of H_3_-15/H-5, H-5/H-9*α*, and H-2/H-13 were observed in the ROESY spectrum (Fig. 2), indicating that compound **7** is a C-13 epimer of compound **6**. Finally, the absolute configuration of **7** was elucidated to be (4*S*,5*S*,7*S*,8*S*,10*S*,13*S*)-7,8,13-trihydroxyrotunda-1,11-dien-3-one based on the ECD calculations (Fig. 5).

Compound **8** was assigned the molecular formula C_15_H_20_O_4_, the same as that of **6** and **7**, by ^13^C NMR data (Table 4) and the ion peak at *m/z* 264.1359 [M]^+^ (calcd for C_15_H_20_O_4_, 264.1362) in the HREIMS. The ^1^H and ^13^C NMR data (Table 4) indicated that this compound might also be a rotundane-type sesquiterpenoid with one *α,β*-unsaturated ketone functionality (*δ*_C_ 214.5, 191.7, and 128.5), one exocyclic double bond (*δ*_C_ 156.3 and 116.5), two quaternary carbon atoms (*δ*_C_ 77.4 and 43.6), one methine, four methylenes including two oxygenated groups (*δ*_C_ 78.6 and 71.8), and two methyl groups (*δ*_C_ 24.5 and 10.5). Based on its ^1^H‒^1^H COSY and HMBC correlations (Fig. 2), the planar structure of **8**, namely, 7,8,13-trihydroxyrotunda-1,11-dien-3-one, was deduced to be the same as that of compounds **6** and **7**. The H-4*α*, H-5*α*, H-8*β*, and H-13*β* configurations were determined by the key ROESY correlations of H_3_-15/H-6*α*, H_3_-15/H-6*β*, H-5/H-9*α*, and H-2/H-13 (Fig. 2) and by comparing *J* values in its ^1^H NMR spectrum with those of compounds **6** and **7**. Finally, the absolute configuration of **8** was elucidated to be (4*R*,5*S*,7*S*,8*S*,10*S*,13*S*)-7,8,13-trihydroxyrotunda-1,11-dien-3-one, a C-4 epimer of **7**, based on the ECD calculations (Fig. 5).

NMR data of C-5 to C-8 in agarotetrol (**9**) were not correctly assigned before [7, 8], which were revised by 2D NMR correlations (Additional file 1: Fig. S2). NMR data of 4ʹ-methoxyagarotetrol (**11**) in DMSO-*d*_6_ [12], 2ʹ-hydroxyagarotetrol (**13**) in DMSO-*d*_6_ [13], (5*S*,6*R*,7*R*,8*S*)-2-(2-phenylethyl)-5,6,7-trihydroxy-5,6,7,8-tetrahydro-8-[2-(2-phenylethyl)chromonyl-6-oxy]chromone (**19**) in DMSO-*d*_6_ [14], and (−)-aquisinenone G (**21**) in CDCl_3_ [15] have been reported in the literature. Their NMR data in methanol-*d*_4_ are presented in this paper. Other known compounds, isoagarotetrol (**10**) [8], 4ʹ-methoxyisoagarotetrol (**12**) [16], (5*S*,6*R*,7*S*,8*R*,7ʹ*R*)-7ʹ-hydroxyisoagarotetrol (**15**) [10], (5*S*,6*R*,7*S*,8*R*,7′*S*)-7′-hydroxyisoagarotetrol (**16**) [10], (5*S*,6*S*,7*S*,8*R*)‑8‑chloro‑2‑(2‑phenylethyl)‑5,6,7‑trihydroxy‑5,6,7,8‑tetrahydrochromone (**14**) [5, 17], 6-hydroxy-2-(2-phenylethyl)chromone (**17**) [18], 2,2ʹ-di-(2-phenylethyl)-8,6ʹ-dihydroxy-5,5ʹ-bichromone (**18**) [19], (5*R*,6*R*,7*R*,8*S*)-2-(2-phenylethyl)-5,6,7-trihydroxy-5,6,7,8-tetrahydro-8-[2-(2-phenylethyl)chromonyl-6-oxy]chromone (**20**) [11], and syringin (**22**) [20], were identified by comparing their spectroscopic data with those in literature.

Rat adrenal pheochromocytoma (PC12) cell injury induced by corticosterone is an in vitro model for screening neuroprotective and antidepressant compounds [6]. All isolates except for compound **3** were evaluated for their protective activities against corticosterone-induced damage in PC12 cells. After testing these compounds at a single concentration of 20 µM (Table 5), several compounds were selected for testing at gradient concentrations of 2.5, 5, 10, 20, and 40 µM. Among them, (6*S*,7*S*,8*R*)-2‑(2-phenylethyl)‑6,7,8‑trihydroxy‑5,6,7,8‑tetrahydrochromone (**4**), (4*S*,5*S*,7*S*,8*S*,10*S*,13*R*)-7,8,13-trihydroxyrotunda-1,11-dien-3-one (**6**), agarotetrol (**9**), and 6-hydroxy-2-(2-phenylethyl)chromone (**17**) showed the most protective activities against corticosterone-induced PC12 cell injury at concentrations from 5 to 40 µM (*P* < 0.001) (Table 6). Among the chromone derivatives (**1**–**5 **and** 9**–**21**), the types and positions of substituent groups seem to have effects on the activity, although no obvious patterns of structure-activity relationships (SAR) were observed. Nevertheless, a hydroxy substituent at C-2ʹ or C-7ʹ would reduce the activity by comparing the bioassay data of agarotetrol (**9**) and their derivatives (**1**, **2**, **11**, and **13**) (Tables 5 and 6).

**Table 5:** The effects of compounds at a single concentration on PC12 cell injury induced by corticosterone^a^

Compound	Concentration (µM)	Survival rate ± SD (%)^b^
Blank	–	100.00 ± 0.22***
Negative control	–	59.92 ± 0.33
Desipramine (positive control)	10	89.66 ± 0.78***
**1**	20	60.24 ± 0.51
**2**	20	59.27 ± 1.01
**4**	20	72.14 ± 1.35***
**6**	20	74.79 ± 0.73***
**7**	20	63.10 ± 0.82**
**9**	20	79.50 ± 1.79***
**10**	20	68.50 ± 1.43***
**11**	20	65.28 ± 1.54**
**12**	20	71.64 ± 1.08***
**17**	20	76.24 ± 1.10***
Blank	–	100.00 ± 0.73***
Negative control	–	60.83 ± 0.93
Desipramine (positive control)	10	90.07 ± 0.45***
**5**	20	60.26 ± 1.14
**8**	20	60.18 ± 1.84
**13**	20	54.29 ± 1.04
**14**	20	60.16 ± 1.20
**15**	20	67.07 ± 1.27**
**16**	20	59.47 ± 0.81
**18**	20	60.27 ± 1.24
**19**	20	60.27 ± 1.55
**20**	20	65.69 ± 0.57**
**21**	20	71.34 ± 1.01***
**22**	20	60.55 ± 1.21

^a^The concentration of corticosterone was 150 μM

^b^Compared with the negative control, **P < 0.01, ***P < 0.001

**Table 6:** The effects of compounds at gradient concentrations on PC12 cell injury induced by corticosterone^a^

Compound	Concentration (µM)	Survival rate ± SD (%)^b^
Blank	–	100.01 ± 0.77***
Negative control	–	60.29 ± 0.44
Desipramine (positive control)	10	89.74 ± 0.58***
**4**	40	70.30 ± 0.12***
	20	72.18 ± 0.33***
	10	67.46 ± 0.61***
	5	64.05 ± 0.50***
	2.5	60.39 ± 0.50
**6**	40	71.77 ± 0.39***
	20	74.22 ± 0.54***
	10	69.90 ± 0.26***
	5	66.76 ± 0.79***
	2.5	60.18 ± 0.53
**7**	40	68.02 ± 0.44***
	20	63.67 ± 0.27***
	10	60.79 ± 0.42
	5	60.16 ± 0.25
	2.5	59.97 ± 0.39
**9**	40	72.00 ± 0.98***
	20	79.11 ± 0.80***
	10	73.53 ± 0.39***
	5	64.87 ± 0.49***
	2.5	60.17 ± 0.41
**10**	40	66.05 ± 0.21***
	20	68.63 ± 1.05***
	10	64.60 ± 0.27***
	5	60.06 ± 0.34
	2.5	60.21 ± 0.30
**11**	40	70.06 ± 0.15***
	20	65.87 ± 0.37***
	10	63.91 ± 0.19***
	5	59.97 ± 0.36
	2.5	60.13 ± 0.38
**12**	40	71.66 ± 0.22***
	20	70.86 ± 0.54***
	10	63.50 ± 0.85**
	5	62.01 ± 0.54*
	2.5	59.98 ± 0.39
**17**	40	77.87 ± 0.70***
	20	76.06 ± 0.40***
	10	71.62 ± 0.40***
	5	66.26 ± 1.07***
	2.5	60.39 ± 0.50

^a^The concentration of corticosterone was 150 μM

^b^Compared with the negative control, *P < 0.05, **P < 0.01, ***P < 0.001

## Experimental section

### General experimental procedures

The material and instruments used for isolating compounds and measuring spectroscopic data are provided in the Additional file 1.

### Plant material

The plant material was purchased from the Flagship Store of Jiabaohua Pharmacy, Zhuhai, Guangdong, China (order number: 172979790097330735) in June 2018, produced by Guangdong Huiqun Chinese Traditional Medicine Co., Ltd., Shantou, Guangdong, China (lot number: 20171101), and identified as the resinous heartwood of *Aquilaria sinensis* (Lour.) Spreng. by Professor Shu-De Yang at Yunnan University of Traditional Chinese Medicine, China. The voucher specimen (No. GD171101) was kept in the Key Laboratory of Economic Plants and Biotechnology, Kunming Institute of Botany, Chinese Academy of Sciences.

### Extraction and isolation

The dried resinous heartwood of *A. sinensis* (2.9 kg) was ultrasonically extracted with 90% EtOH (10 L × 5) at 60 °C for half an hour each time. The crude extract (459.3 g) was suspended in 1 L of water, followed by extraction with petroleum ether (1 L × 5), EtOAc (1 L × 5), and *n*-BuOH (1 L × 5). After removing the solvent, the petroleum ether-soluble fraction (0.7 g), EtOAc-soluble fraction (374.8 g), and *n*-BuOH-soluble fraction (55.9 g) were obtained.

The *n*-BuOH-soluble fraction (55.9 g) was separated by a silica gel column with EtOAc/MeOH (100:0 → 0:1, v/v) as the eluent to yield five further fractions (Fr. 1 to Fr. 5). Fr. 1 (786.8 mg) was subjected to a reversed-phase (RP) C_18_ silica gel column (MeOH/H_2_O, 5% → 100%) to yield 10 further fractions (Fr. 1-1 to Fr. 1-10). Compound **17** (2.1 mg) is obtained from Fr. 1–6 by recrystallization (MeOH).

Fr. 2 (16.7 g) was purified by an RP C_18_ column (MeOH/H_2_O, 5% → 100%) to yield 13 further fractions (Fr. 2-1 to Fr. 2-13). Fr. 2-6 was recrystallized from MeOH to yield **9** (1.1 g). Fr. 2-3 (456.6 mg) was applied to a silica gel column via elution by CH_2_Cl_2_/MeOH (50:1 → 1:1) to yield five further fractions (Fr. 2-3-1 to Fr. 2-3-5). Fr. 2-3-2 (114.7 mg) was purified by semipreparative HPLC (Welch Ultimate AQ-C_18_, *ϕ* 7.8 × 250 mm; MeOH/H_2_O, 20:80, *v* = 2 mL/min) to yield **6** (10.6 mg, *t*_R_ = 15.072 min) and **7** (11.9 mg, *t*_R_ = 22.831 min). Fr. 2-3-5 (58.3 mg) was further purified by semipreparative HPLC (Welch Ultimate AQ-C_18_, *ϕ* 7.8 × 250 mm; MeOH/H_2_O, 40:60, *v* = 2 mL/min) to yield **1** (14.1 mg, *t*_R_ = 9.282 min) and **2** (7.1 mg, *t*_R_ = 10.161 min). Fr. 2-3-4 (41.3 mg) was separated by a silica gel column (CH_2_Cl_2_/MeOH, 30:1 → 1:1) and was then purified by semipreparative HPLC (Welch Ultimate AQ-C_18_, *ϕ* 7.8 × 250 mm; MeCN/H_2_O, 10:90, *v* = 2 mL/min) to yield **15** (1.5 mg, *t*_R_ = 22.158 min). Fr. 2-8 (524.3 mg) was separated by a silica gel column (CH_2_Cl_2_/MeOH, 50:1) to yield 11 further fractions (Fr. 2-8-1 to Fr. 2-8-11). Fr. 2-8-6 was recrystallized from MeOH to yield **12** (204.0 mg). Fr. 2-8-9 (51.3 mg) was purified by semipreparative HPLC (Welch Ultimate AQ-C_18_, *ϕ* 7.8 × 250 mm; MeOH/H_2_O, 30:70, *v* = 2 mL/min) to yield **4** (4.2 mg, *t*_R_ = 37.011 min) and **3** (2.0 mg, *t*_R_ = 41.898 min). Fr. 2-8-10 (49.3 mg) was applied to a silica gel column via elution by CH_2_Cl_2_/MeOH (30:1 → 1:1) and was further purified by semipreparative HPLC (Welch Ultimate AQ-C_18_, *ϕ* 7.8 × 250 mm; MeOH/H_2_O, 33:67, *v* = 2 mL/min) to yield **11** (1.4 mg, *t*_R_ = 21.190 min). Fr. 2-9 (489.4 mg) was applied to a silica gel column and eluted with CH_2_Cl_2_/MeOH (100:1 → 1:1) to yield five further fractions (Fr. 2–9-1 to Fr. 2-9-5). Fr. 2-9-2 (118.8 mg) was separated by Sephadex LH-20 (MeOH) and was further purified by semipreparative HPLC (YMC-Pack ODS-A, *ϕ* 10 × 250 mm; MeOH/H_2_O, 40:60, *v* = 2 mL/min) to yield **14** (2.9 mg, *t*_R_ = 14.865 min). Fr 2-9-4 (46.9 mg) was purified by Sephadex LH-20 (MeOH) to yield **10** (4.0 mg). Fr 2-12 (599.0 mg) was subjected to a silica gel column via elution by CH_2_Cl_2_/MeOH (100:1 → 1:1) to yield five further fractions (Fr. 2-12-1 to Fr. 2-12-5). Fr. 2-12-1 was recrystallized from MeOH to yield **18** (1.2 mg). Fr. 2-12-2 (164.1 mg) was applied to a silica gel column (CH_2_Cl_2_/MeOH, 100:1 → 1:1) to yield three further fractions (Fr. 2-12-2-1 to Fr. 2-12-2-3). Fr. 2-12-2-1 (80.0 mg) was purified by semipreparative HPLC (YMC-Pack ODS-A, *ϕ* 10 × 250 mm; MeCN/H_2_O, 48:52, *v* = 2 mL/min) to yield **21** (4.0 mg, *t*_R_ = 27.886 min). Fr. 2-12-2-3 (60.9 mg) was purified by semipreparative HPLC (Welch Ultimate AQ-C_18_, *ϕ* 7.8 × 250 mm; MeOH/H_2_O, 65:35, *v* = 2 mL/min) to yield **5** (2.3 mg, *t*_R_ = 26.957 min). Fr. 2-12-4 (29.0 mg) was separated by Sephadex LH-20 gel column chromatography (MeOH) and then by semipreparative HPLC (Welch Ultimate AQ-C_18_, *ϕ* 7.8 × 250 mm; MeOH/H_2_O, 65:35, *v* = 2 mL/min) to yield **19** (3.7 mg, *t*_R_ = 25.515 min) and **20** (3.9 mg, *t*_R_ = 30.107 min).

Fr. 3 (6.0 g) was separated by an RP C_18_ silica gel column (MeOH/H_2_O, 5% → 100%) to yield 11 further fractions (Fr. 3-1 to Fr. 3-11). Fr. 3–3 (158.0 mg) was separated by a silica gel column (CH_2_Cl_2_/MeOH, 50:1) and then purified by semipreparative HPLC (Welch Ultimate AQ-C_18_, *ϕ* 7.8 × 250 mm; MeOH/H_2_O, 30:70, *v* = 2 mL/min) to yield **8** (0.8 mg, *t*_R_ = 10.757 min). Fr. 3-4 (195.5 mg) was separated by a silica gel column (CH_2_Cl_2_/MeOH, 50:1) and was then purified by semipreparative HPLC (YMC-Pack ODS-A, *ϕ* 10 × 250 mm; MeOH/H_2_O, 35:65, *v* = 2 mL/min) to yield **22** (7.1 mg, *t*_R_ = 11.198 min) and **16** (0.9 mg, *t*_R_ = 15.727 min). Fr. 3-5 (39.0 mg) was purified by semipreparative HPLC (YMC-Pack ODS-A, *ϕ* 10 × 250 mm; MeCN/H_2_O, 20:80, *v* = 2 mL/min) to yield **13** (7.5 mg, *t*_R_ = 31.475 min).

### Spectroscopic data of compounds 1–9, 11, 13, and 19–21

#### (5*S*,6*R*,7*R*,8*S*,7′*R*)-7′-hydroxyagarotetrol (1)

Colorless needle crystal (MeOH); [*α*]_D_^21^ − 29.1 (*c* = 0.13, MeOH); UV (MeOH) *λ*_max_ (log*ε*) 252 (4.03), 207 (4.17) nm; ECD (*c* 0.013, MeOH) *λ*_max_ (Δ*ε*) 298 (+ 0.68), 261 (− 0.14), 245 (+ 0.13), 222 (− 1.02), 212 (+ 1.35), 194 (+ 3.92) nm; IR *v*_max_ (KBr) 3406, 1658, 1601, 1448, 1089, 1057, 1039, 1018, 701 cm^−1^; ^1^H NMR and ^13^C NMR data see Table 1; ESIMS (positive) *m/z* 357 [M + Na]^+^, 691 [2 M + Na]^+^; HRESIMS (positive) *m/z* 357.0942 [M + Na]^+^ (calcd for C_17_H_18_NaO_7_, 357.0950).

Crystal data of compound **1**: C_17_H_18_O_7_·2(H_2_O), *M* = 370.34, *a* = 5.5739(3) Å, *b* = 8.0353(5) Å, *c* = 19.5345(12) Å, *α* = 90°, *β* = 97.596(2)°, *γ* = 90°, *V* = 867.23(9) Å^3^, *T* = 100(2) K, space group *P*1211, *Z* = 2, *μ*(Cu K*α*) = 0.987 mm^−1^, 7517 reflections measured, 2687 independent reflections (*R*_*int*_ = 0.0345). The final *R*_*1*_ values were 0.0287 [*I* > 2*σ*(*I*)]. The final *wR*(*F*^2^) values were 0.0916 [*I* > 2*σ*(*I*)]. The final *R*_*1*_ values were 0.0292 (all data). The final *wR*(*F*^2^) values were 0.0929 (all data). The goodness of fit on *F*^2^ was 0.837. Flack parameter = 0.11(10). The crystallographic data for the structure of **1** have been deposited in the Cambridge Crystallographic Data Centre (deposition number CCDC 2,118,605). Copies of the data can be obtained free of charge from the CCDC via www.ccdc.cam.ac.uk.

#### (5*S*,6*R*,7*R*,8*S*,7′*S*)-7′-hydroxyagarotetrol (2)

Light yellow powder; [*α*]_D_^21^ − 28.3 (*c* = 0.35, MeOH); UV (MeOH) *λ*_max_ (log*ε*) 252 (3.85), 207 (4.00) nm; ECD (*c* 0.018, MeOH) *λ*_max_ (Δ*ε*) 301 (+ 0.14), 254 (− 0.93), 223 (+ 1.69) nm; IR *v*_max_ (KBr) 3423, 1658, 1601, 1447, 1384, 1044, 703 cm^−1^; ^1^H NMR and ^13^C NMR data see Table 1; ESIMS (positive) *m/z* 357 [M + Na]^+^; HRESIMS (positive) *m/z* 357.0945 [M + Na]^+^ (calcd for C_17_H_18_NaO_7_, 357.0950).

#### (6*S*,7*S*,8*R*)-2‑[2‑(4-methoxyphenyl)ethyl]‑6,7,8‑trihydroxy‑5,6,7,8‑tetrahydrochromone (3)

Colorless plate crystal (MeOH); [*α*]_D_^27^ + 7.4 (*c* = 0.23, MeOH); UV (MeOH) *λ*_max_ (log*ε*) 435 (1.81), 365 (2.05), 254 (3.88), 220 (3.99), 201(4.04) nm; ECD (*c* 0.0099, MeOH) *λ*_max_ (Δ*ε*) 221 (− 1.43) nm; ^1^H NMR and ^13^C NMR data see Table 2; ESIMS (positive) *m/z* 302 [M + Na]^+^, 627 [2 M + Na]^+^; HRESIMS (positive) *m/z* 355.1150 [M + Na]^+^ (calcd for C_18_H_20_NaO_6_, 355.1158).

Crystal data of compound **3**: C_18_H_20_O_6_, *M* = 332.34, *a* = 4.9601(2) Å, *b* = 7.0550(2) Å, *c* = 23.2769(7) Å, *α* = 90°, *β* = 93.5950(10)°, *γ* = 90°, *V* = 812.94(5) Å^3^, *T* = 100.(2) K, space group *P*1211, *Z* = 2, *μ*(Cu K*α*) = 0.850 mm^−1^, 12,448 reflections measured, 3180 independent reflections (*R*_*int*_ = 0.0352). The final *R*_*1*_ values were 0.0271 [*I* > 2*σ*(*I*)]. The final *wR*(*F*^2^) values were 0.0704 [*I* > 2*σ*(*I*)]. The final *R*_*1*_ values were 0.0277 (all data). The final *wR*(*F*^2^) values were 0.0710 (all data). The goodness of fit on *F*^2^ was 1.031. Flack parameter = 0.09(4). The crystallographic data for the structure of **3** have been deposited in the Cambridge Crystallographic Data Centre (deposition number CCDC 2118606). Copies of the data can be obtained free of charge from the CCDC via www.ccdc.cam.ac.uk.

#### (6*S*,7*S*,8*R*)-2‑(2-phenylethyl)‑6,7,8‑trihydroxy‑5,6,7,8‑tetrahydrochromone (4)

Yellow powder; [*α*]_D_^27^ + 14.2 (*c* = 0.50, MeOH); UV (MeOH) *λ*_max_ (log*ε*) 372 (2.22), 254 (4.07), 209 (4.19) nm; ECD (*c* 0.0072, MeOH) *λ*_max_ (Δ*ε*) 233 (− 3.14) nm; ^1^H NMR and ^13^C NMR data see Table 2; ESIMS (positive) *m/z* 302 [M + Na]^+^, 627 [2 M + Na]^+^; HRESIMS (positive) *m/z* 325.1046 [M + Na]^+^ (calcd for C_17_H_18_NaO_5_, 325.1052).

#### (5*S*,6*R*,7*S*,8*R*)-2-(2-phenylethyl)-5,6,7-trihydroxy-5,6,7,8-tetrahydro-8- [2-(2-phenylethyl)-7-methoxychromonyl-6-oxy]chromone (5)

Yellow powder; [*α*]_D_^20^ + 98.6 (*c* = 0.10, MeOH); UV (MeOH) *λ*_max_ (log*ε*) 314 (4.29), 237 (4.85), 204 (5.02) nm; ECD (*c* 0.0041, MeOH) *λ*_max_ (Δ*ε*) 320 (+ 5.57), 280 (− 3.87), 249 (+ 9.33), 229 (− 1.47), 207 (− 20.23) nm; IR *v*_max_ (KBr) 3387, 1657, 1603, 1504, 1453, 1384, 1271, 1216, 1197, 1101, 1080, 998, 957, 847, 749, 700 cm^−1^; ^1^H NMR and ^13^C NMR data see Table 3; ESIMS (positive) *m/z* 619 [M + Na]^+^; HRESIMS (positive) *m/z* 619.1939 [M + Na]^+^ (calcd C_35_H_32_NaO_9_, 619.1944).

#### (4*S*,5*S*,7*S*,8*S*,10*S*,13*R*)-7,8,13-trihydroxyrotunda-1,11-dien-3-one (6)

Pale yellow oil; [*α*]_D_^27^ − 95.4 (*c* = 0.50, MeOH); UV (MeOH) *λ*_max_ (log*ε*) 234 (4.12) (3.81) nm; ECD (*c* 0.015, MeOH) *λ*_max_ (Δ*ε*) 246 (+ 2.59), 203 (− 13.63) nm; IR *ν*_max_ (KBr) 3416, 2962, 2932, 2872, 1686, 1683, 1600, 1456, 1384, 1281, 1196, 1082, 1044, 1025, 997, 930 cm^−1^; ^1^H NMR and ^13^C NMR data see Table 4; ESIMS (negative) *m/z* 299 [M + Cl]^−^, 309 [M + HCOO^−^]^+^; ESIMS (positive) *m/z* 287 [M + Na]^+^, 551 [2 M + Na]^+^; HRESIMS (positive) *m/z* 287.1255 [M + Na]^+^ (calcd for C_15_H_20_NaO_4_, 287.1259).

#### (4*S*,5*S*,7*S*,8*S*,10*S*,13*S*)-7,8,13-trihydroxyrotunda-1,11-dien-3-one (7)

Pale yellow oil; [*α*]_D_^27^ − 87.1 (*c* = 0.09, MeOH); UV (MeOH) *λ*_max_ (log*ε*) 236 (4.02) nm; ECD (*c* 0.018, MeOH) *λ*_max_ (Δ*ε*) 258 (+ 0.52), 222 (− 6.96) nm; IR *ν*_max_ (KBr) 3425, 2964, 2927, 2874, 2852, 1688, 1633, 1597, 1456, 1384, 1284, 1188, 1073, 1055, 1028, 982, 928 cm^−1^; ^1^H NMR and ^13^C NMR data see Table 4; ESIMS (positive) *m/z* 287 [M + Na]^+^, 551 [2 M + Na]^+^; HRESIMS (positive) *m/z* 287.1253 [M + Na]^+^ (calcd for C_15_H_20_NaO_4_, 287.1259).

#### (4*R*,5*S*,7*S*,8*S*,10*S*,13*S*)-7,8,13-trihydroxyrotunda-1,11-dien-3-one (8)

Pale yellow oil; [*α*]_D_^25^ − 13.3 (*c* = 0.05, MeOH); UV (MeOH) *λ*_max_ (log*ε*) 235 (4.01) nm; ECD (*c* 0.015, MeOH) *λ*_max_ (Δ*ε*) 223 (− 6.08) nm; ^1^H NMR and ^13^C NMR data see Table 4; ESIMS (positive) *m/z* 287 [M + Na]^+^, 551 [2 M + Na]^+^; EIMS *m/z* (rel. int.) 264 [M]^+^ (30), 246 (15), 179 (47), 136 (77), 91 (68), 55 (90), 43 (100); HREIMS *m/z* 264.1359 [M]^+^ (calcd for C_15_H_20_O_4_, 264.1362).

#### Agarotetrol (9)

White solid; [*α*]_D_^18^ − 17.9 (*c* = 0.20, MeOH); ^1^H NMR (methanol-*d*_4_, 500 MHz)* δ* 7.26 (2H, m, H-3ʹ,5ʹ), 7.22 (2H, m, H-2ʹ,6ʹ), 7.18 (1H, m, H-4ʹ), 6.12 (1H, s, H-3), 4.74 (1H, d, *J* = 4.0 Hz, H-5), 4.57 (1H, d, *J* = 7.5 Hz, H-8), 4.04 (1H, dd, *J* = 7.5, 2.3 Hz, H-7), 4.02 (1H, dd, *J* = 4.0, 2.3 Hz, H-6), 3.03 (2H, dd, *J* = 7.7, 7.3 Hz, H_2_-7ʹ), 2.93 (2H, m, H_2_-8ʹ); ^13^C NMR (methanol-*d*_4_, 126 MHz) *δ* 182.0 (C-4), 171.2 (C-2), 165.4 (C-8a), 141.2 (C-1ʹ), 129.6 (C-3ʹ,5ʹ), 129.5 (C-2ʹ,6ʹ), 127.5 (C-4ʹ), 121.8 (C-4a), 114.1 (C-3), 74.0 (C-6), 72.4 (C-7), 70.1 (C-8), 66.7 (C-5), 36.3 (C-8ʹ), 33.7 (C-7ʹ); ESIMS (positive) *m/z* 341 [M + Na]^+^, 659 [2 M + Na]^+^.

#### 4′-Methoxyagarotetrol (11)

Light yellow solid; [*α*]_D_^24^ − 18.3 (*c* = 0.10, MeOH); ^1^H NMR (methanol-*d*_4_, 500 MHz) *δ* 7.13 (2H, br d, *J* = 8.6 Hz, H-2ʹ,6ʹ), 6.82 (2H, br d, *J* = 8.6 Hz, H-3ʹ,5ʹ), 6.10 (1H, s, H-3), 4.74 (1H, d, *J* = 4.0 Hz, H-5), 4.56 (1H, d, *J* = 7.5 Hz, H-8), 4.04 (1H, dd, *J* = 7.5, 2.3 Hz, H-7), 4.01 (1H, dd, *J* = 4.0, 2.3 Hz, H-6), 3.74 (3H, s, 4ʹ-OMe), 2.89 (2H, m, H_2_-8ʹ), 2.97 (2H, m, H_2_-7ʹ); ^13^C NMR (methanol-*d*_4_, 126 MHz) *δ* 182.0 (C-4), 171.4 (C-2), 165.4 (C-8a), 159.8 (C-4ʹ), 133.1 (C-1ʹ), 130.4 (C-2ʹ,6ʹ), 121.7 (C-4a), 115.0 (C-3ʹ,5ʹ), 114.1 (C-3), 74.0 (C-6), 72.4 (C-7), 70.1 (C-8), 66.7 (C-5), 55.6 (4ʹ-OMe), 36.5 (C-8ʹ), 32.9 (C-7ʹ); ESIMS (positive) *m/z* 371 [M + Na]^+^, 719 [2 M + Na]^+^; HRESIMS (positive) *m/z* 371.1100 [M + Na]^+^ (calcd for C_18_H_20_NaO_7_, 371.1107).

#### 2′-Hydroxyagarotetrol (13)

Light yellow solid; [*α*]_D_^28^ − 8.3 (*c* = 0.18, MeOH); ^1^H NMR (methanol-*d*_4_, 500 MHz) *δ* 7.05 (1H, dd, *J* = 7.4, 1.6 Hz, H-6ʹ), 7.01 (1H, ddd, *J* = 8.0, 7.4, 1.6 Hz, H-4ʹ), 6.74 (1H, br d, *J* = 8.0 Hz, H-3ʹ), 6.72 (1H, ddd, *J* = 7.4, 7.4, 1.2 Hz, H-5ʹ), 6.10 (1H, s, H-3), 4.74 (1H, d, *J* = 4.0 Hz, H-5), 4.56 (1H, d, *J* = 7.4 Hz, H-8), 4.04 (1H, dd, *J* = 7.4, 2.3 Hz, H-7), 4.02 (1H, dd, *J* = 4.0, 2.3 Hz, H-6), 3.00 (2H, m, H_2_-7ʹ), 2.92 (2H, m, H_2_-8ʹ); ^13^C NMR (methanol-*d*_4_, 126 MHz) *δ* 182.1 (C-4), 172.0 (C-2), 165.4 (C-8a), 156.5 (C-2ʹ), 131.2 (C-6ʹ), 128.7 (C-4ʹ), 127.3 (C-1ʹ), 121.6 (C-4a), 120.6 (C-5ʹ), 115.9 (C-3ʹ), 113.8 (C-3), 74.0 (C-6), 72.5 (C-7), 70.1 (C-8), 66.7 (C-5), 34.7 (C-8ʹ), 29.0 (C-7ʹ); ESIMS (positive) *m/z* 357 [M + Na]^+^, 691 [2 M + Na]^+^.

#### (5*S*,6*R*,7*R*,8*S*)-2-(2-Phenylethyl)-5,6,7-trihydroxy-5,6,7,8-tetrahydro-8- [2-(2-phenylethyl)chromonyl-6-oxy]chromone (19)

Yellow powder; [*α*]_D_^24^ − 75.8 (*c* = 0.38, MeOH); ^1^H NMR (methanol-*d*_4_, 500 MHz) Unit A *δ* 7.15 (2H, m, H-3ʹ,5ʹ), 7.10 (1H, m, H-4ʹ), 6.92 (2H, m, H-2ʹ,6ʹ), 6.15 (1H, s, H-3), 5.46 (1H, d, *J* = 7.7 Hz, H-8), 4.80 (1H, d, *J* = 3.7 Hz, H-5), 4.38 (1H, dd, *J* = 7.7, 2.3 Hz, H-7), 4.10 (1H, dd, *J* = 3.7, 2.3 Hz, H-6), 2.71 (2H, m, H_2_-8ʹ), 2.62 (2H, m, H_2_-7ʹ); Unit B *δ* 7.89 (1H, d, *J* = 2.5 Hz, H-5), 7.62 (1H, dd, *J* = 9.2, 2.5 Hz, H-7), 7.60 (1H, d, *J* = 9.2 Hz, H-8), 7.24 (2H, m, H-3ʹ,5ʹ), 7.22 (2H, m, H-2ʹ,6ʹ), 7.16 (1H, m, H-4ʹ), 6.16 (1H, s, H-3), 3.08 (2H, m, H_2_-7ʹ), 3.03 (2H, m, H_2_-8ʹ); ^13^C NMR (methanol-*d*_4_, 126 MHz) Unit A *δ* 181.5 (C-4), 170.9 (C-2), 162.8 (C-8a), 140.9 (C-1ʹ), 129.4 (C-3ʹ,5ʹ), 129.2 (C-2ʹ,6ʹ), 127.4 (C-4ʹ), 122.7 (C-4a), 114.5 (C-3), 78.2 (C-8), 74.5 (C-6), 70.8 (C-7), 66.3 (C-5), 36.2 (C-8ʹ), 33.5 (C-7ʹ); Unit B *δ* 180.2 (C-4), 171.6 (C-2), 158.6 (C-6), 153.4 (C-8a), 141.2 (C-1ʹ), 129.6 (C-3ʹ,5ʹ), 129.5 (C-2ʹ,6ʹ), 127.5 (C-4ʹ), 126.2 (C-7), 125.0 (C-4a), 120.8 (C-8), 110.4 (C-5), 110.1 (C-3), 37.0 (C-8ʹ), 34.0 (C-7ʹ); ESIMS (positive) *m/z* 589 [M + Na]^+^; HRESIMS (positive) *m/z* 589.1831 [M + Na]^+^ (calcd for C_34_H_30_NaO_8_, 589.1838).

#### (5*S*,6*R*,7*S*,8*R*)-2-(2-phenylethyl)-5,6,7-trihydroxy-5,6,7,8-tetrahydro-8-[2-(2-phenylethyl)chromonyl-6-oxy]chromone (20)

Yellow powder; [*α*]_D_^23^ + 22.5 (*c* = 0.23, MeOH); ECD (*c* 0.01, MeOH) *λ*_max_ (Δ*ε*) 243 (+ 8.07), 216 (− 8.85) nm; ESIMS (positive) *m/z* 567 [M + H]^+^; HRESIMS (positive) *m/z* 589.1833 [M + Na]^+^ (calcd C_34_H_30_NaO_8_, 589.1838).

#### (−)-Aquisinenone G (21)

Yellow powder; [*α*]_D_^20^ − 26.8 (*c* = 0.22, MeOH); UV (MeOH) *λ*_max_ (log*ε*) 314 (3.99), 229 (4.58) nm; ECD (*c* 0.007, MeOH) *λ*_max_ (Δ*ε*) 313 (− 1.36), 277 (+ 4.38), 249 (− 5.58), 221 (− 7.71), 205 (+ 8.69) nm; ^1^H NMR (methanol-*d*_4_, 500 MHz) Unit A *δ* 7.16 (1H, m, H-4ʹ), 7.08 (2H, m, H-3ʹ,5ʹ), 6.95 (2H, m, H-2ʹ,6ʹ), 6.03 (1H, s, H-3), 5.52 (1H, dd, *J* = 3.0, 2.0 Hz, H-5), 4.77 (1H, m, H-7), 4.59 (1H, dd, *J* = 3.3, 3.0 Hz, H-6), 4.45 (1H, br s, H-8), 2.86 (2H, m, H_2_-7ʹ), 2.80 (2H, m, H_2_-8ʹ); Unit B *δ* 7.67 (1H, s, H-5), 7.23 (2H, m, H-3ʹ,5ʹ), 7.20 (2H, m, H-2ʹ,6ʹ), 7.06 (1H, m, H-4ʹ), 6.89 (1H, s, H-8), 6.12 (1H, s, H-3), 3.02 (2H, m, H_2_-7ʹ), 2.96 (2H, m, H_2_-8ʹ); ^13^C NMR (methanol-*d*_4_, 126 MHz) Unit A *δ* 179.8 (C-4), 171.5 (C-2), 166.0 (C-8a), 140.8 (C-1ʹ), 129.4 (C-2ʹ,3ʹ,5ʹ,6ʹ), 127.5 (C-4ʹ), 117.4 (C-4a), 114.5 (C-3), 79.3 (C-7), 72.6 (C-5), 69.7 (C-6), 66.8 (C-8), 36.1 (C-8ʹ), 33.6 (C-7ʹ); Unit B *δ* 179.5 (C-4), 171.6 (C-2), 155.0 (C-8a), 154.5 (C-7), 147.0 (C-6), 141.2 (C-1ʹ), 129.6 (C-3ʹ,5ʹ), 129.3 (C-2ʹ,6ʹ), 127.3 (C-4ʹ), 120.9 (C-4a), 119.1 (C-5), 112.5 (C-8), 110.2 (C-3), 36.9 (C-8ʹ), 33.9 (C-7ʹ); ESIMS (positive) *m/z* 587 [M + Na]^+^.

### Computational methods

Theoretical calculations of ECD spectra for compounds **6**–**8** were performed with the Gaussian 16 program package [21]. The preliminary conformational distribution search was performed by Tripos sybyl- × 2 software [22]. Selected conformers with distributions higher than 1% were further optimized by the DFT method at the B3LYP/6-311 g (d) level in the Gaussian 16 program package. The ECD of the conformer of selected conformers was then calculated by the TD-DFT method at the CAM-B3LYP/tzvp levels with the PCM model in methanol solution. The overall calculated ECD curves were weighted by Boltzmann distribution. The calculated ECD spectra were produced by SpecDis 1.71 [23]. Detailed calculated parameters are provided in the Additional file 1.

### Corticosterone‑induced damage in PC12 cellular assay

The method for bioassay testing was carried out according to our previously published papers [5, 6].

## Conclusion

Five new 2-(2-phenylethyl)chromone derivatives, three new sesquiterpenoids, and 14 known compounds were isolated from the resinous heartwood of *Aquilaria sinensis*. The neuroprotective activities of these isolates were evaluated using an in vitro model of PC12 cell injury induced by corticosterone. (6*S*,7*S*,8*R*)-2‑(2-Phenylethyl)‑6,7,8‑trihydroxy‑5,6,7,8‑tetrahydrochromone (**4**), (4*S*,5*S*,7*S*,8*S*,10*S*,13*R*)-7,8,13-trihydroxyrotunda-1,11-dien-3-one (**6**), agarotetrol (**9**), and 6-hydroxy-2-(2-phenylethyl)chromone (**17**) showed the most protective activities against corticosterone-induced PC12 cell injury at concentrations from 5 to 40 µM (*P* < 0.001).

## Supplementary Information

**Additional file 1.** Chemical structures of known compounds (**9–22**), key 2D NMR correlations of agarotetrol (**9**), general experimental procedures, computational methods for ECD of compounds **6–8**, and NMR, HRMS, and ECD spectra of compounds **1–8**.
