# Supplementary material for: Five new 2-(2-phenylethyl)chromone derivatives and three new sesquiterpenoids from the heartwood of Aquilaria sinensis, an aromatic medicine in China
Source: Nat Prod Bioprospect. 2022 Jan 28;12(1):2. doi: 10.1007/s13659-022-00326-3 (PMC8795264; doi:10.1007/s13659-022-00326-3)

Supplementary Material for

Five New 2-(2-Phenylethyl)chromone Derivatives and Three New Sesquiterpenoids from the Heartwood of *Aquilaria sinensis*, an Aromatic Medicine in China

Lu Zhang[1] · Ping Yi[2] · Hui Yan[1] · Xiao-Nian Li[1] · Meng-Yuan Xia[1] · Jun Yang[1] · Ji-Feng Luo[1] · Yue-Qiu He[3] · Yue-Hu Wang[1]

Lu Zhang and Ping Yi contributed equally to this work.

✉ Yue-Qiu He
ynfh2007@163.com

✉ Yue-Hu Wang
wangyuehu@mail.kib.ac.cn

[1] Key Laboratory of Economic Plants and Biotechnology, Yunnan Key Laboratory for Wild Plant Resources, and State Key Laboratory of Phytochemistry and Plant Resources in West China, Kunming Institute of Botany, Chinese Academy of Sciences, Kunming 650201, People's Republic of China

[2] Key Laboratory of Chemistry for Natural Products of Guizhou Province and Chinese Academy of Sciences, Guiyang 550014, People's Republic of China

[3] Faculty of Plant Protection, Yunnan Agricultural University, Kunming 650201, People's Republic of China

Contents

**Fig. S1** Chemical structures of known compounds (**9**–**22**) from *Aquilaria sinensis*
**Fig. S2** Key 2D NMR correlations of agarotetrol (**9**)
**General Experimental Procedures**
**Computational methods for ECD of compounds 6–8.**
**Fig. S3.** ^{1}H NMR spectrum of **1** (methanol-d_4, 500 MHz).
**Fig. S4.** ^{13}C NMR spectrum of **1** (methanol-d_4, 126 MHz).
**Fig. S5.** HSQC spectrum of **1**.
**Fig. S6.** ^{1}H–^{1}H COSY spectrum of **1**.
**Fig. S7.** HMBC spectrum of **1**.
**Fig. S8.** ROESY spectrum of **1**.
**Fig. S9.** HRESIMS spectrum of **1**.
**Fig. S10.** ECD spectrum of **1**.
**Fig. S11.** ^{1}H NMR spectrum of **2** (methanol-d_4, 500 MHz).
**Fig. S12.** ^{13}C NMR spectrum of **2** (methanol-d_4, 126 MHz).
**Fig. S13.** HSQC spectrum of **2**.
**Fig. S14.** ^{1}H–^{1}H COSY spectrum of **2**.
**Fig. S15.** HMBC spectrum of **2**.
**Fig. S16.** ROESY spectrum of **2**.
**Fig. S17.** HRESIMS spectrum of **2**.
**Fig. S18.** ECD spectrum of **2**.
**Fig. S19.** ^{1}H NMR spectrum of **3** (methanol-d_4, 600 MHz).
**Fig. S20.** ^{13}C NMR spectrum of **3** (methanol-d_4, 151 MHz).
**Fig. S21.** HSQC spectrum of **3**.
**Fig. S22.** ^{1}H–^{1}H COSY spectrum of **3**.
**Fig. S23.** HMBC spectrum of **3**.
**Fig. S24.** ROESY spectrum of **3**.
**Fig. S25.** HRESIMS spectrum of **3**.
**Fig. S26.** ECD spectrum of **3**.
**Fig. S27.** ^{1}H NMR spectrum of **4** (methanol-d_4, 500 MHz).
**Fig. S28.** ^{13}C NMR spectrum of **4** (methanol-d_4, 126 MHz).
**Fig. S29.** HSQC spectrum of **4**.
**Fig. S30.** ^{1}H–^{1}H COSY spectrum of **4**.
**Fig. S31.** HMBC spectrum of **4**.
**Fig. S32.** ROESY spectrum of **4**.
**Fig. S33.** HRESIMS spectrum of **4**.
**Fig. S34.** ECD spectrum of **4**.
**Fig. S35.** ^{1}H NMR spectrum of **5** (methanol-d_4, 600 MHz).
**Fig. S36.** ^{13}C NMR spectrum of **5** (methanol-d_4, 151 MHz).
**Fig. S37.** HSQC spectrum of **5**.
**Fig. S38.** ^{1}H–^{1}H COSY spectrum of **5**.
**Fig. S39.** HMBC spectrum of **5**.
**Fig. S40.** ROESY spectrum of **5**.

**Fig. S41.** HRESIMS spectrum of **5**.
**Fig. S42.** ECD spectrum of **5**.
**Fig. S43.** ^{1}H NMR spectrum of **6** (methanol-d_4, 500 MHz).
**Fig. S44.** ^{13}C NMR spectrum of **6** (methanol-d_4, 126 MHz).
**Fig. S45.** HSQC spectrum of **6**.
**Fig. S46.** ^{1}H–^{1}H COSY spectrum of **6**.
**Fig. S47.** HMBC spectrum of **6**.
**Fig. S48.** ROESY spectrum of **6**.
**Fig. S49.** HRESIMS spectrum of **6**.
**Fig. S50.** ECD spectrum of **6**.
**Fig. S51.** ^{1}H NMR spectrum of **7** (methanol-d_4, 800 MHz).
**Fig. S52.** ^{13}C NMR spectrum of **7** (methanol-d_4, 201 MHz).
**Fig. S53.** HSQC spectrum of **7**.
**Fig. S54.** ^{1}H–^{1}H COSY spectrum of **7**.
**Fig. S55.** HMBC spectrum of **7**.
**Fig. S56.** ROESY spectrum of **7**.
**Fig. S57.** HRESIMS spectrum of **7**.
**Fig. S58.** ECD spectrum of **7**.
**Fig. S59.** ^{1}H NMR spectrum of **8** (methanol-d_4, 800 MHz).
**Fig. S60.** ^{13}C NMR spectrum of **8** (methanol-d_4, 201 MHz).
**Fig. S61.** HSQC spectrum of **8**.
**Fig. S62.** ^{1}H–^{1}H COSY spectrum of **8**.
**Fig. S63.** HMBC spectrum of **8**.
**Fig. S64.** ROESY spectrum of **8**.
**Fig. S65.** HREIMS spectrum of **8**.
**Fig. S66.** ECD spectrum of **8**.
**Fig. S67.** ^{1}H NMR spectrum of **9** (methanol-d_4, 500 MHz).
**Fig. S68.** ^{13}C NMR spectrum of **9** (methanol-d_4, 126 MHz).
**Fig. S69.** HSQC spectrum of **9**.
**Fig. S70.** ^{1}H–^{1}H COSY spectrum of **9**.
**Fig. S71.** HMBC spectrum of **9**.
**Fig. S72.** ROESY spectrum of **9**.

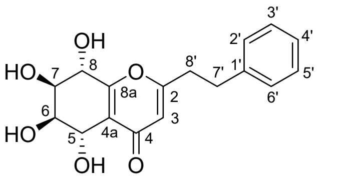

agarotetrol (**9**)

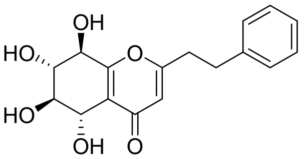

isoagarotetrol (**10**)

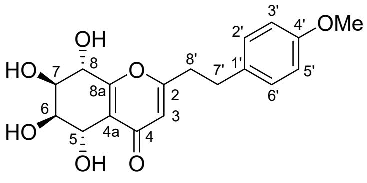

4'-methoxyagarotetrol (**11**)

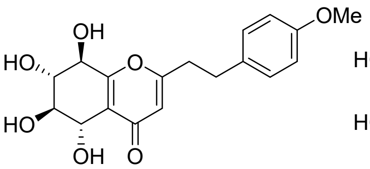

4'-methoxyisoagarotetrol (**12**)

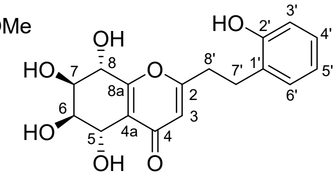

2'-hydroxyagarotetrol (**13**)

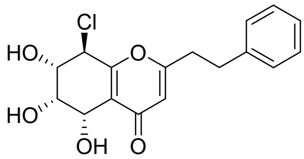

(5*S*,6*S*,7*S*,8*R*)-8-chloro-2-(2-phenylethyl)-5,6,7-trihydroxy-5,6,7,8-tetrahydrochromone (**14**)

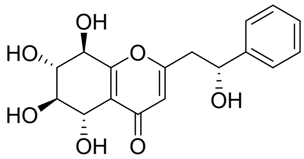

(5*S*,6*R*,7*S*,8*R*,7'*R*)-7'-hydroxyisoagarotetrol (**15**)

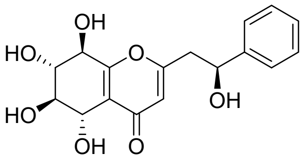

(5*S*,6*R*,7*S*,8*R*,7'*S*)-7'-hydroxyisoagarotetrol (**16**)

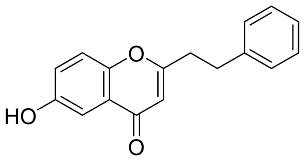

6-hydroxy-2-(2-phenylethyl)chromone (**17**)

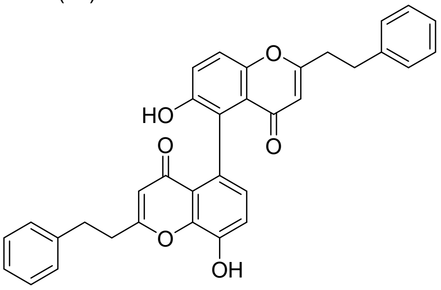

2,2'-di-(2-phenylethyl)-8,6'-dihydroxy-5,5'-bichromone (**18**)

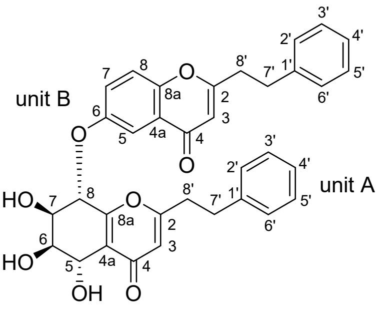

(5*S*,6*R*,7*R*,8*S*)-2-(2-phenylethyl)-5,6,7-trihydroxy-5,6,7,8-tetrahydro-8-[2-(2-phenylethyl)chromonyl-6-oxy]chromone (**19**)

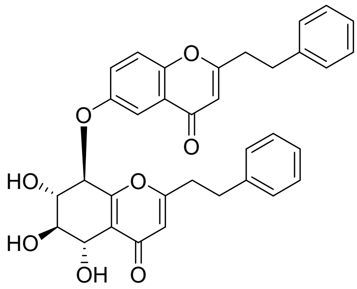

(5*S*,6*R*,7*S*,8*R*)-2-(2-phenylethyl)-5,6,7-trihydroxy-5,6,7,8-tetrahydro-8-[2-(2-phenylethyl)chromonyl-6-oxy]chromone (**20**)

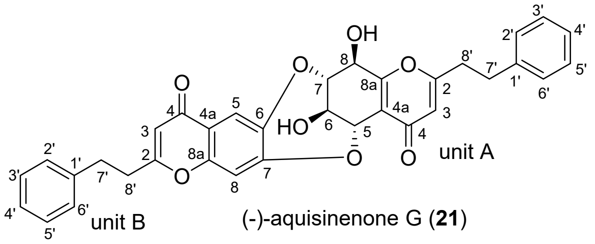

(-)-aquisinenone G (**21**)

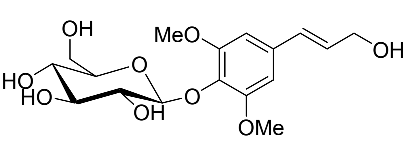

syringin (**22**)

Fig. S1 Chemical structures of known compounds (**9**–**22**) from *Aquilaria sinensis*

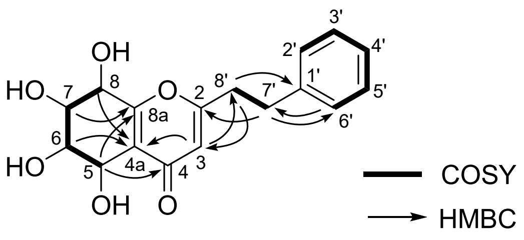

Fig. S2 Key 2D NMR correlations of agarotetrol (**9**)

General Experimental Procedures

Optical rotations were collected on a JASCO P-1020 Polarimeter (Jasco Corp., Tokyo, Japan). UV spectra were obtained using a Shimadzu UV-2401 PC spectrophotometer (Shimadzu, Kyoto, Japan). Electronic circular dichroism (ECD) spectra were recorded on a Chirascan CD spectrometer (Applied Photophysics Ltd., Leatherhead, UK). IR spectra were measured on a Bruker Tensor 27 FTIR Spectrometer (Bruker Corp., Ettlingen, Germany) with KBr disks. 1D and 2D nuclear magnetic resonance (NMR) spectra were measured on Bruker AM-400, DRX-500, Avance III-600, and Ascend^{TM} 800 MHz spectrometers (Bruker Corporation, Karlsruhe, Germany), with tetramethylsilane (TMS) as an internal standard. The chemical shifts (δ) were expressed in ppm as a reference to the solvent signals. ESIMS and HRESIMS analyses were performed on an API QSTAR Pulsar 1 spectrometer (Applied Biosystems/MDS Sciex, Foster City, CA, USA). EIMS and HREIMS were recorded on a Waters AutoSpec Premier p776 spectrometer (Waters, Millford, MA, USA). Silica gel G (80–100 and 300–400 mesh, Qingdao Meigao Chemical Co., Ltd., Qingdao, China), reversed-phase (RP) C_{18} silica gel (40–75 μm, Fuji Silysia Chemical Ltd., Aichi, Japan), and Sephadex LH-20 (GE Healthcare Bio-Sciences AB, Uppsala, Sweden) were used for column chromatography. Silica gel GF_{254} (Qingdao Meigao Chemical Co., Ltd., Qingdao, China) was used for thin-layer chromatography (TLC) to monitor the fraction by heating the silica gel plates after immersion in 5% H_2SO_4 in EtOH. Semipreparative high-performance liquid chromatography (HPLC) was performed on an Agilent 1200 series pump and an Agilent 1260 series pump (Agilent Technologies, Santa Clara, USA) equipped with a diode array detector, a Welch Ultimate AQ-C_{18} column (5.0 μm, ϕ 7.8 × 250 mm), and a YMC-Pack ODS-A column (5.0 μm, ϕ 10 × 250 mm).

Computational Methods for ECD of Compounds 6–8.

1. Calculations for ECD of 6

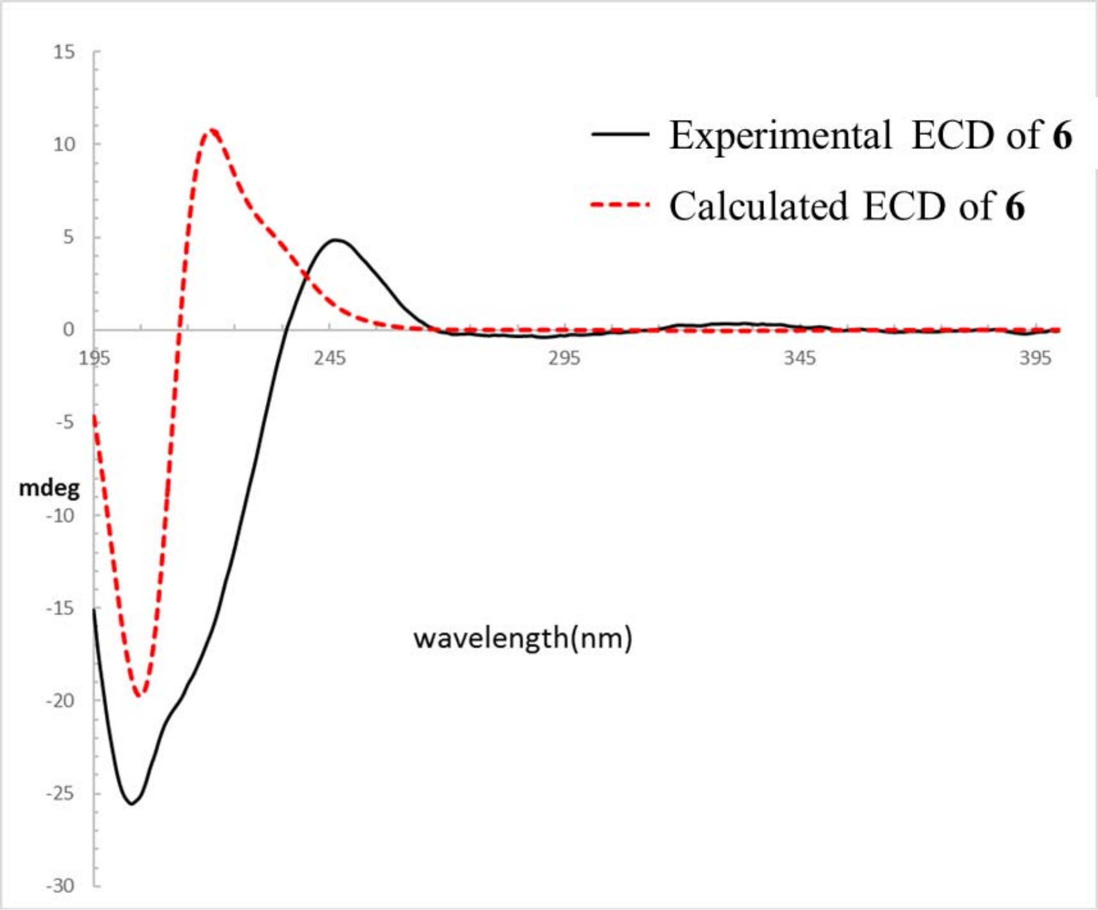

b3lyp/6-311+g(d,2p) optimized lowest energy 3D conformer of 6

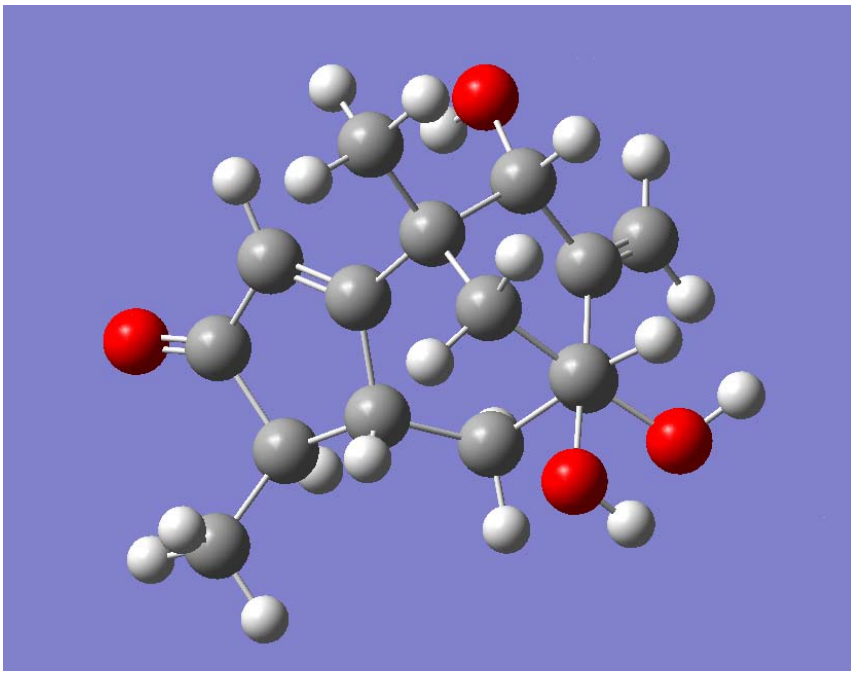

Absolute configuration of 6

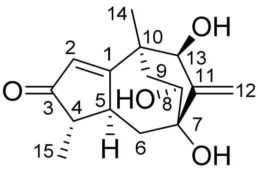

Computational methods for ECD of compound 6

--

td=(50-50,nstates=50) cam-b3lyp/tzvp scrf=(solvent= methanol)

--

Input orientation:

Center Number	Atomic Number	Atomic Type	Coordinates (Angstroms) X	Y	Z
1	6	0	-0.845059	0.600869	0.400641
2	6	0	-0.965070	-0.896823	0.155733
3	6	0	0.156470	-1.496375	-0.715728
4	6	0	1.580177	-0.943413	-0.475526
5	6	0	1.734675	0.449064	-1.081395
6	6	0	1.367901	1.630704	-0.185498
7	6	0	0.420211	1.202010	0.984405
8	6	0	-1.969111	1.257680	0.055858

9	6	0	-2.988411	0.324188	-0.454822
10	6	0	-2.380914	-1.087804	-0.440374
11	6	0	-3.276394	-2.085592	0.302796
12	8	0	-4.115441	0.599943	-0.812754
13	6	0	0.105898	2.427704	1.859873
14	8	0	0.882205	2.739902	-0.923310
15	6	0	2.155122	0.636260	-2.333417
16	8	0	2.435809	-1.904602	-1.120439
17	1	0	-0.914086	-1.390541	1.130186
18	6	0	1.194581	0.157881	1.833426
19	6	0	1.975350	-0.891853	1.018832
20	8	0	1.801251	-2.159888	1.641058
21	1	0	0.190242	-2.573350	-0.550172
22	1	0	-0.065481	-1.340157	-1.772142
23	1	0	2.279493	2.006273	0.290147
24	1	0	-2.168293	2.312799	0.166075
25	1	0	-2.304188	-1.401179	-1.485630
26	1	0	-2.865071	-3.094854	0.251458
27	1	0	-4.273191	-2.095404	-0.137238
28	1	0	-3.375773	-1.813521	1.355718
29	1	0	-0.397319	3.208353	1.294913
30	1	0	-0.526025	2.143590	2.702230
31	1	0	1.030332	2.852608	2.254866
32	1	0	0.123503	2.449321	-1.442518
33	1	0	2.246572	1.631738	-2.744485
34	1	0	2.400618	-0.196900	-2.977777
35	1	0	3.295000	-1.494033	-1.267053
36	1	0	1.892458	0.694199	2.477219
37	1	0	0.508323	-0.366093	2.497983
38	1	0	3.042470	-0.631947	1.036083
39	1	0	2.257256	-2.810739	1.094877

----

2. **Calculations for ECD of 7**

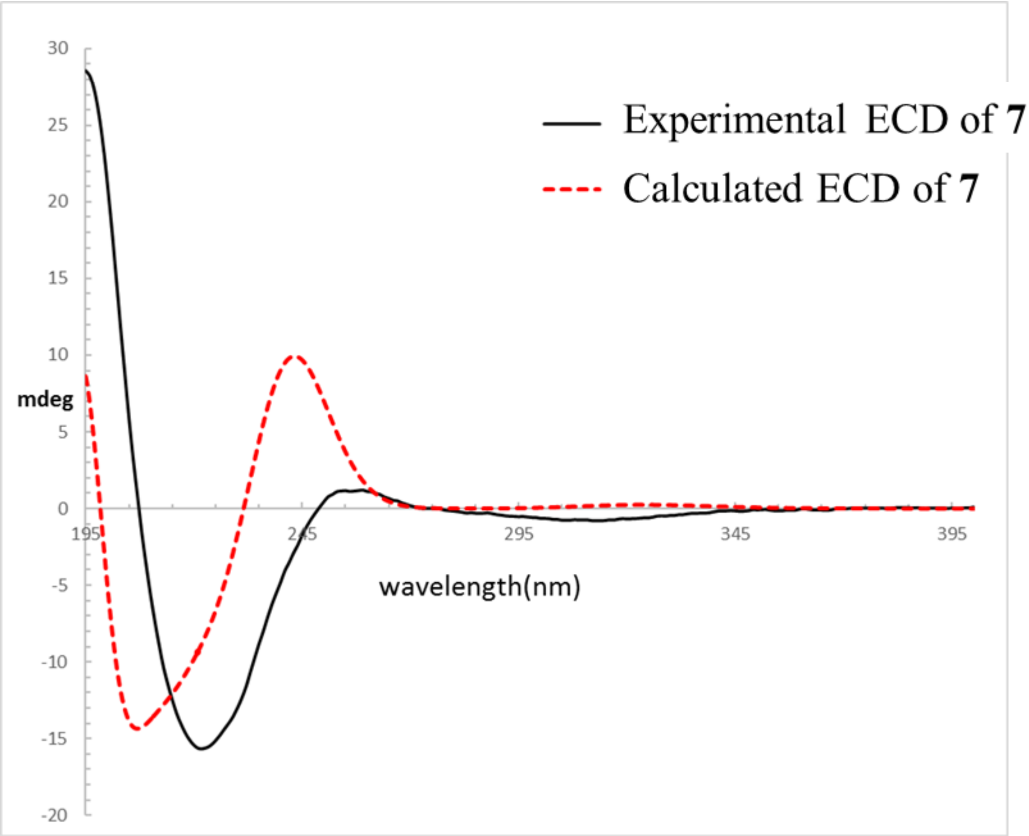

b3lyp/6-311+g(d,2p) optimized lowest energy 3D conformer of 7

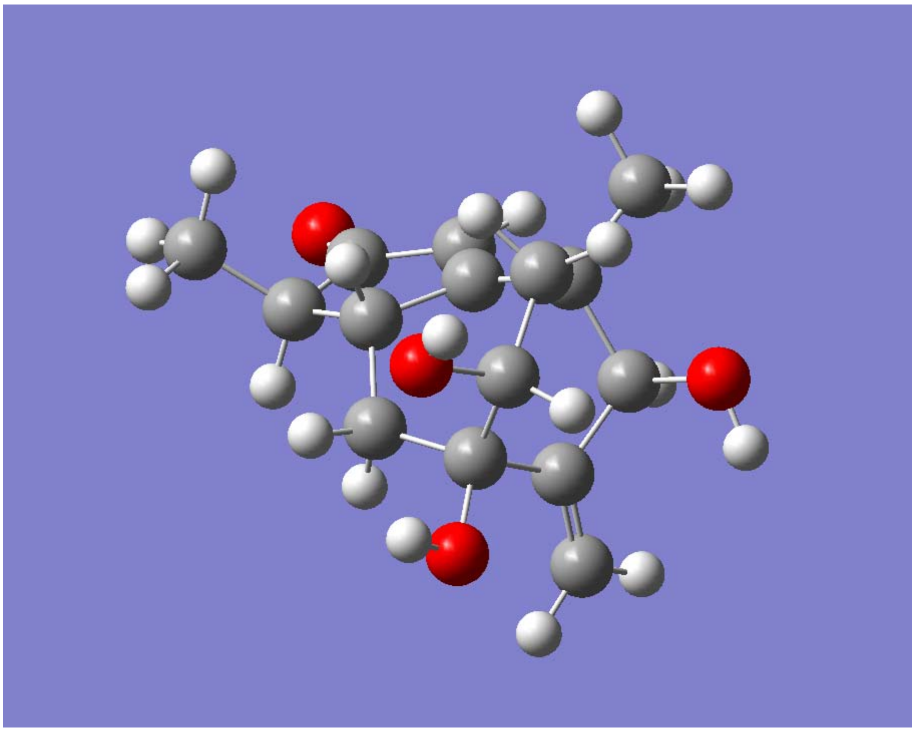

Absolute configuration of 7

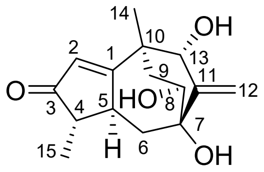

Computational methods for ECD of compound 7

---

td=(50-50,nstates=50) cam-b3lyp/tzvp scrf=(solvent= methanol)

Input orientation:

Center Number	Atomic Number	Atomic Type	Coordinates (Angstroms) X	Y	Z
1	6	0	0.872501	0.764305	-0.224300
2	6	0	1.133299	-0.734923	-0.283278
3	6	0	0.091708	-1.605370	0.453349
4	6	0	-1.385117	-1.123117	0.412692
5	6	0	-1.545308	0.115546	1.283771
6	6	0	-1.359092	1.458574	0.603946
7	6	0	-0.463899	1.350497	-0.657718
8	6	0	1.949994	1.444507	0.206752
9	6	0	3.067181	0.531820	0.499217
10	6	0	2.578069	-0.903357	0.246971
11	6	0	3.529830	-1.681771	-0.667411
12	8	0	4.185188	0.838444	0.861730
13	6	0	-0.282743	2.741220	-1.292655
14	8	0	-2.632068	1.974166	0.166083
15	6	0	-1.848186	0.041333	2.579748
16	8	0	-2.201182	-2.157922	0.966219
17	1	0	1.107599	-1.025098	-1.337675
18	6	0	-1.176015	0.404547	-1.667251
19	6	0	-1.872929	-0.812247	-1.016893
20	8	0	-1.681507	-2.023032	-1.772690
21	1	0	0.126222	-2.611868	0.033268
22	1	0	0.352493	-1.685207	1.509187
23	1	0	-0.905958	2.165986	1.304683
24	1	0	2.050643	2.514285	0.307824
25	1	0	2.555305	-1.393708	1.224680
26	1	0	3.204805	-2.717023	-0.782800
27	1	0	4.536480	-1.679975	-0.250207

28	1	0	3.576959	-1.228555	-1.659991
29	1	0	0.189969	3.438735	-0.601062
30	1	0	0.341240	2.675986	-2.185117
31	1	0	-1.251081	3.152727	-1.570867
32	1	0	-3.263946	1.834186	0.877244
33	1	0	-1.902824	0.934661	3.190553
34	1	0	-2.054510	-0.904585	3.059082
35	1	0	-2.186785	-2.885462	0.334117
36	1	0	-1.922296	0.986284	-2.205787
37	1	0	-0.455534	0.047657	-2.403322
38	1	0	-2.943380	-0.615431	-0.933200
39	1	0	-2.254616	-2.006238	-2.542980

----

3. Calculations for ECD of 8

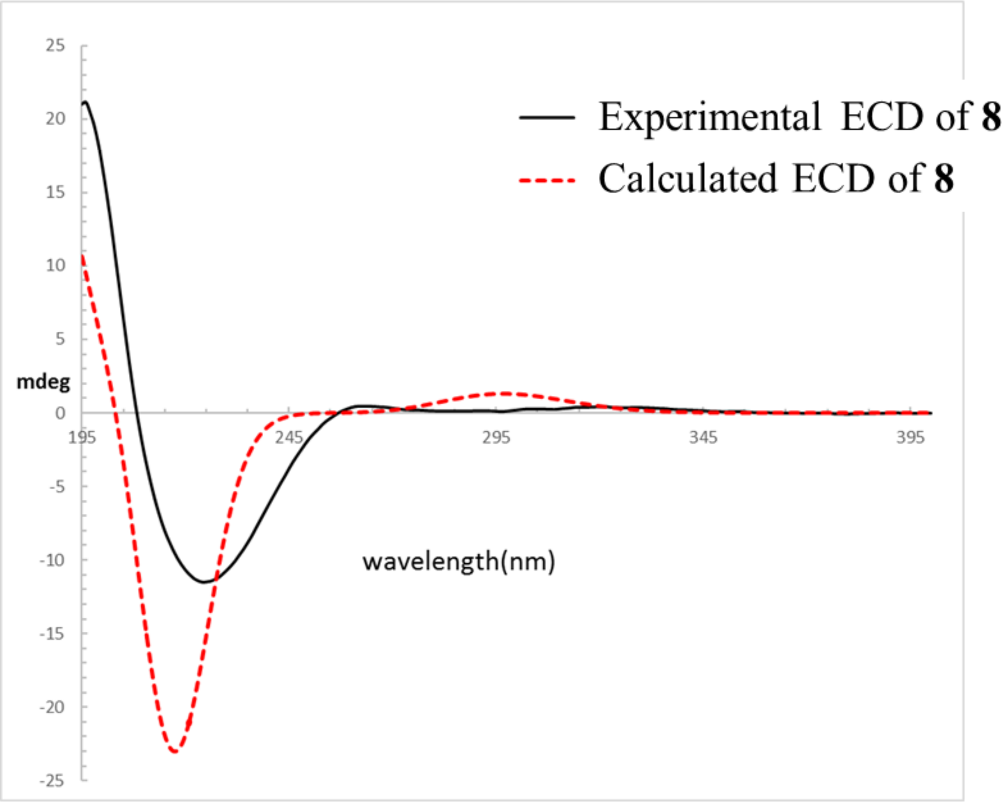

b3lyp/6-311+g(d,2p) optimized lowest energy 3D conformer of 8

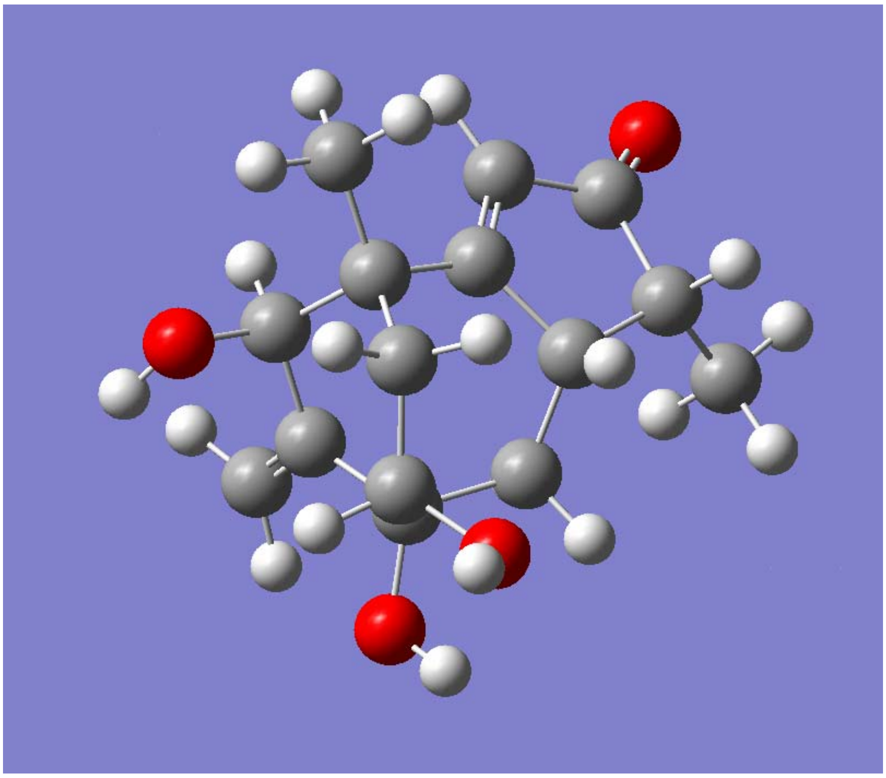

Absolute configuration of 8

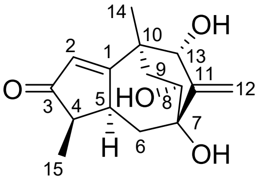

Computational methods for ECD of compound 8

----

td=(50-50,nstates=50) cam-b3lyp/tzvp scrf=(solvent= methanol)

Input orientation:

Center Number	Atomic Number	Atomic Type	Coordinates (Angstroms) X	Y	Z
1	6	0	-0.796873	0.885126	-0.351349
2	6	0	-1.121324	-0.554208	-0.720143
3	6	0	-0.284121	-1.575378	0.081693
4	6	0	1.211865	-1.210945	0.311072
5	6	0	1.326486	-0.112822	1.360926
6	6	0	1.314140	1.319817	0.859935
7	6	0	0.611411	1.439363	-0.517356
8	6	0	-1.864416	1.532990	0.147835
9	6	0	-3.044900	0.653088	0.178367
10	6	0	-2.664937	-0.652910	-0.537994
11	6	0	-3.264314	-1.894776	0.124611
12	8	0	-4.131825	0.904459	0.658433
13	6	0	0.579127	2.910052	-0.971495
14	8	0	2.661542	1.799817	0.684259
15	6	0	1.447059	-0.380673	2.661041
16	8	0	1.870913	-2.372457	0.821200
17	1	0	-0.872493	-0.706042	-1.772358
18	6	0	1.425915	0.594878	-1.536041
19	6	0	1.925560	-0.759570	-0.980351
20	8	0	1.759663	-1.836780	-1.921925
21	1	0	-0.315305	-2.531463	-0.442149
22	1	0	-0.710113	-1.731345	1.071442
23	1	0	0.796762	1.956994	1.583145
24	1	0	-1.905022	2.551747	0.502594
25	1	0	-3.114761	-0.559550	-1.532732
26	1	0	-3.038869	-2.798502	-0.443108
27	1	0	-4.346843	-1.783755	0.177143

28	1	0	-2.901972	-2.031791	1.143367
29	1	0	0.027643	3.533730	-0.267899
30	1	0	1.592410	3.301257	-1.043263
31	1	0	0.098664	2.998041	-1.947045
32	1	0	3.174468	1.516932	1.446751
33	1	0	1.471761	0.417332	3.393662
34	1	0	1.529309	-1.394641	3.024391
35	1	0	1.895891	-3.012008	0.100622
36	1	0	0.822550	0.413087	-2.425436
37	1	0	2.287317	1.183026	-1.847899
38	1	0	2.983534	-0.677525	-0.723837
39	1	0	2.438669	-1.770021	-2.597638

Fig. S3. ^{1}H NMR spectrum of **1** (methanol-d_4, 500 MHz).

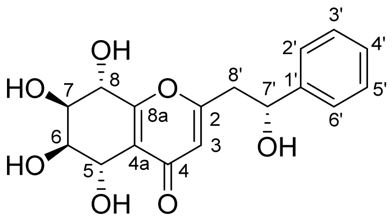

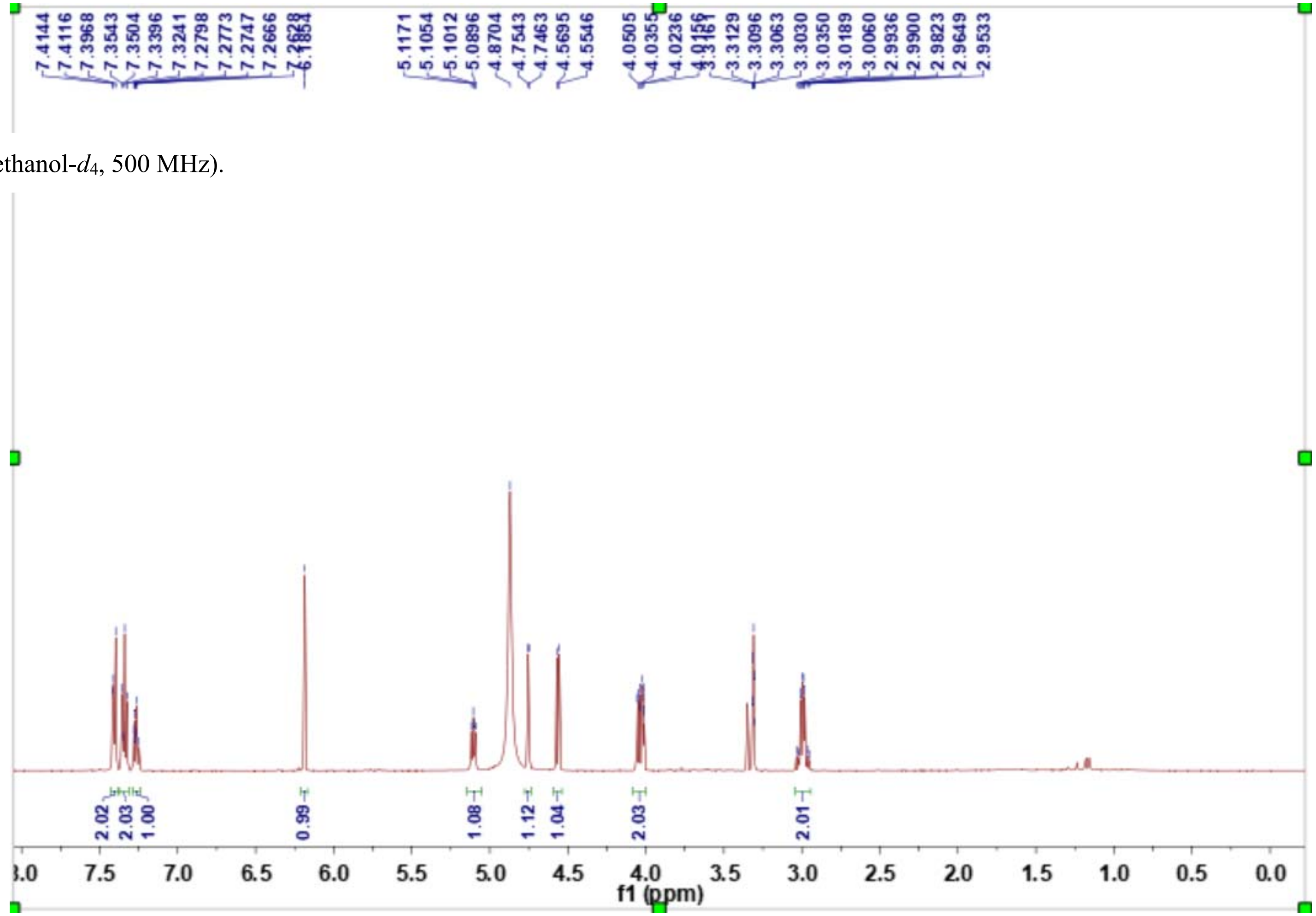

Fig. S4. ^{13}C NMR spectrum of **1** (methanol-d_4, 126 MHz).

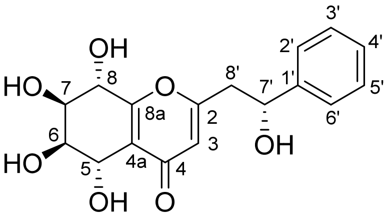

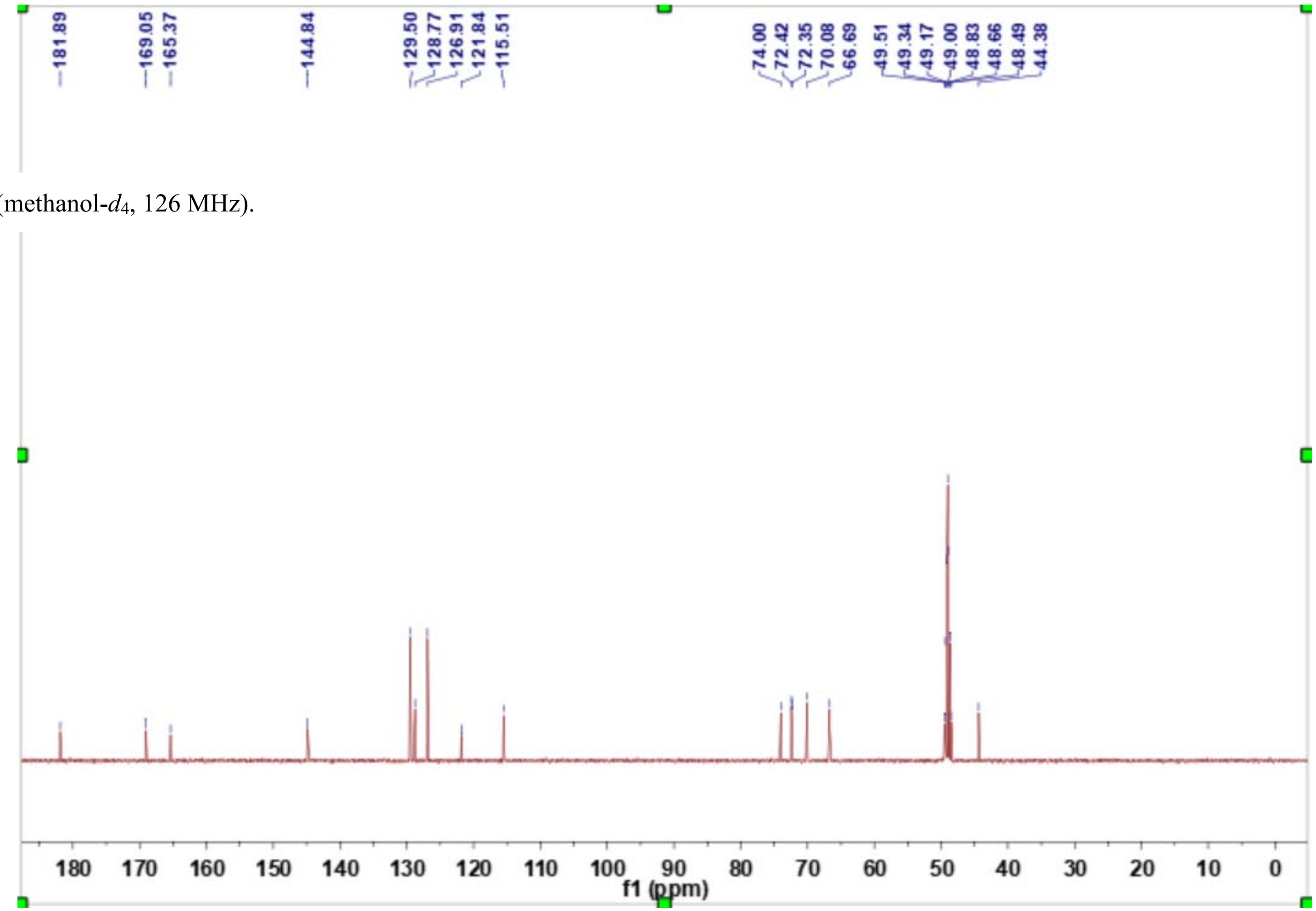

Fig. S5. HSQC spectrum of **1**.

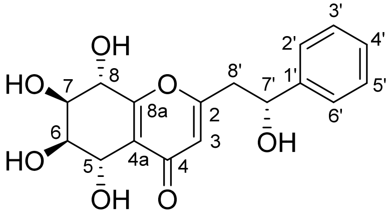

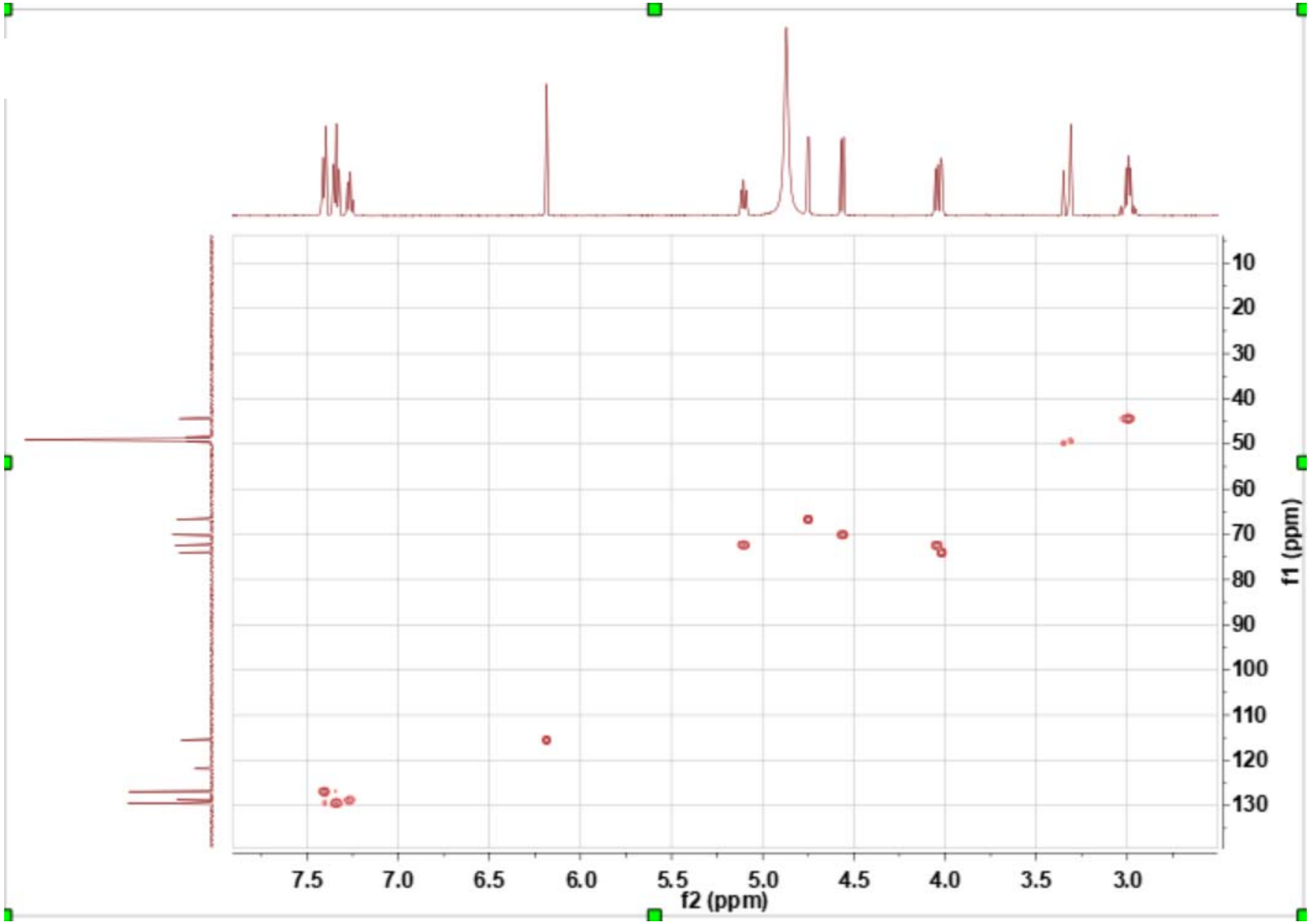

Fig. S6. ^1H–^1H COSY spectrum of **1**.

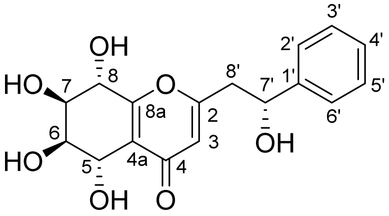

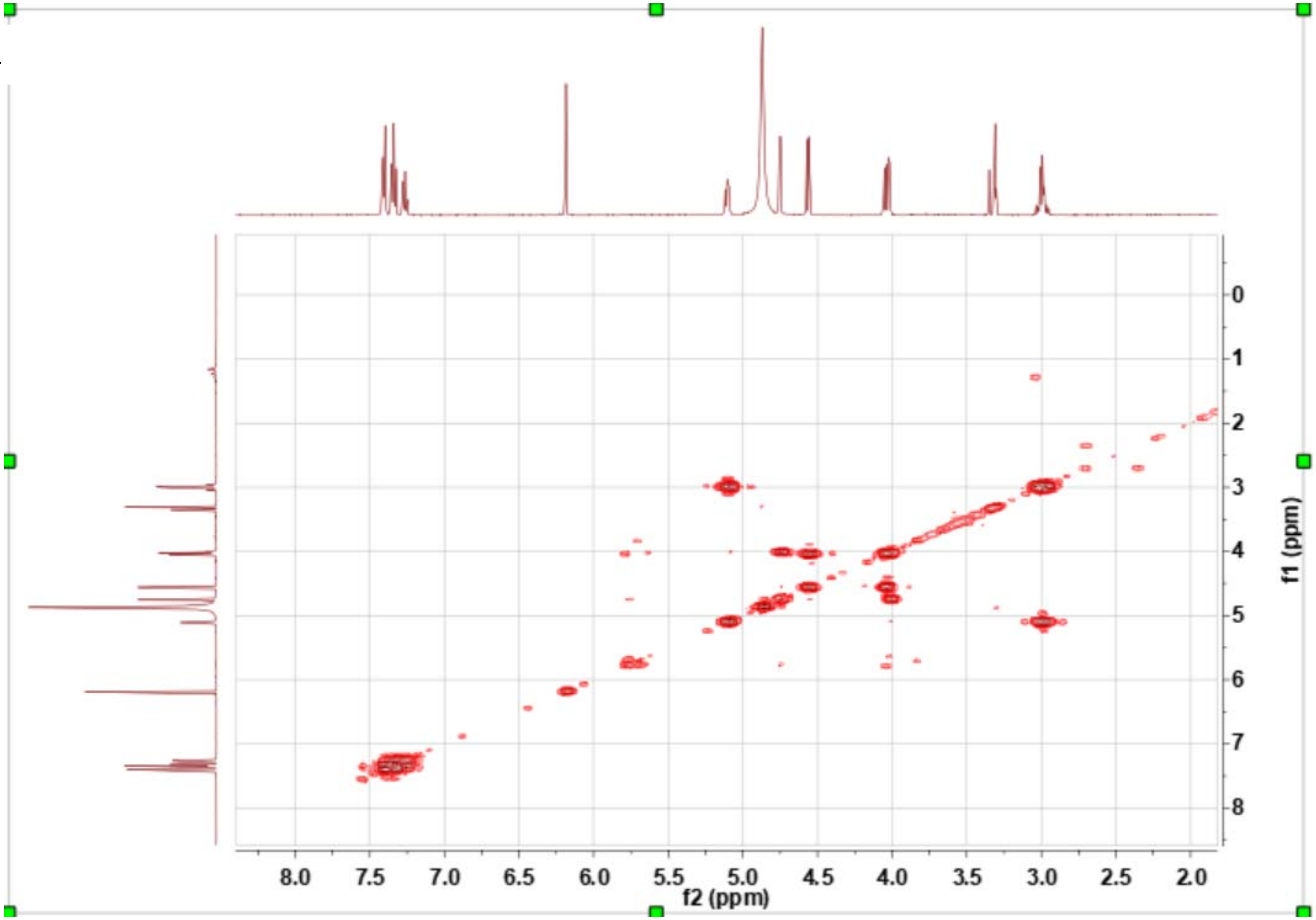

Fig. S7. HMBC spectrum of **1**.

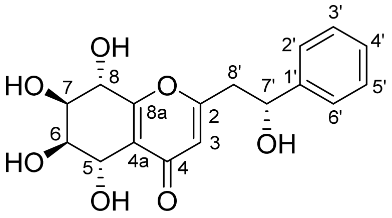

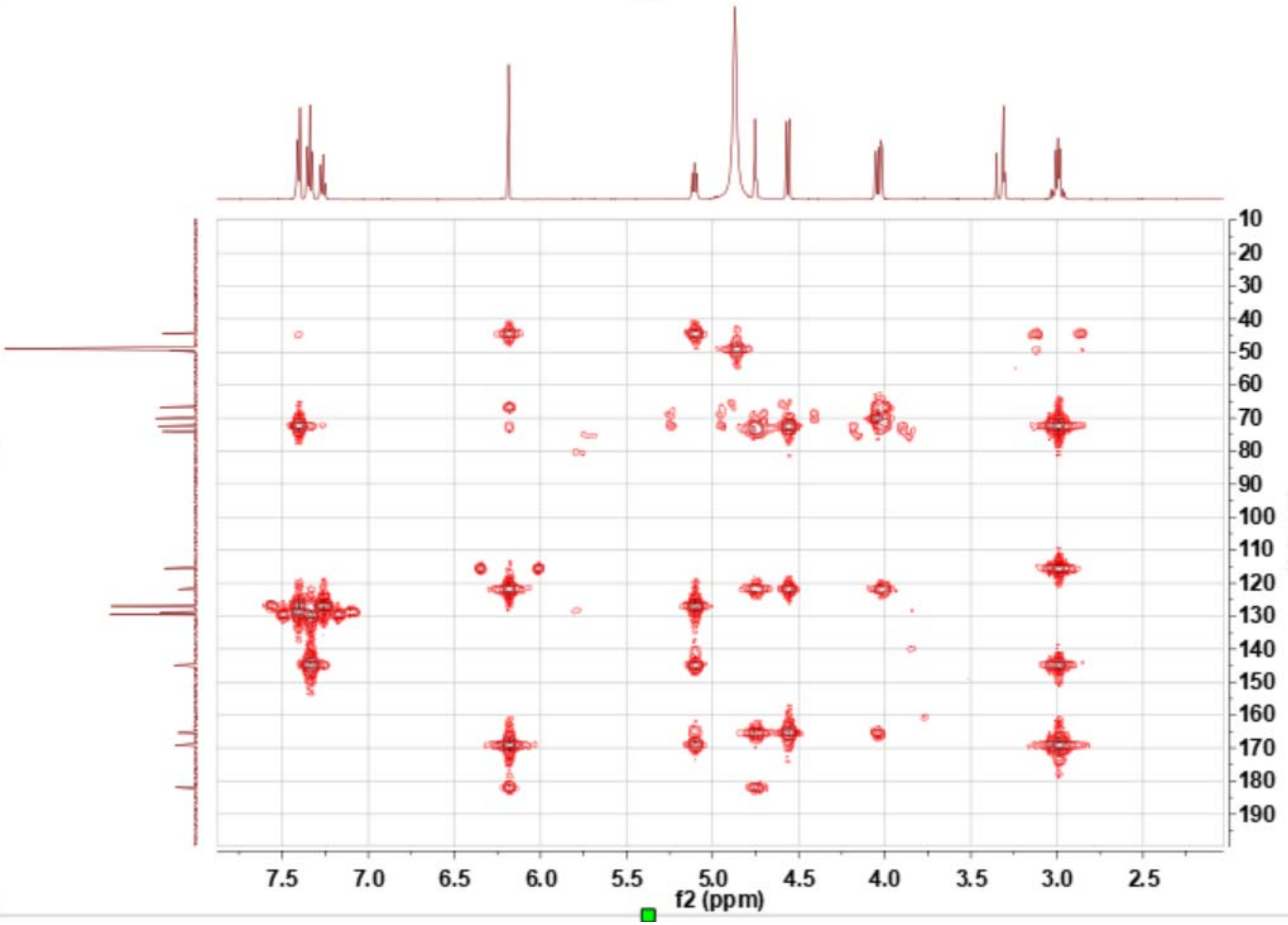

Fig. S8. ROESY spectrum of **1**.

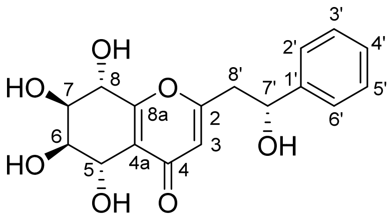

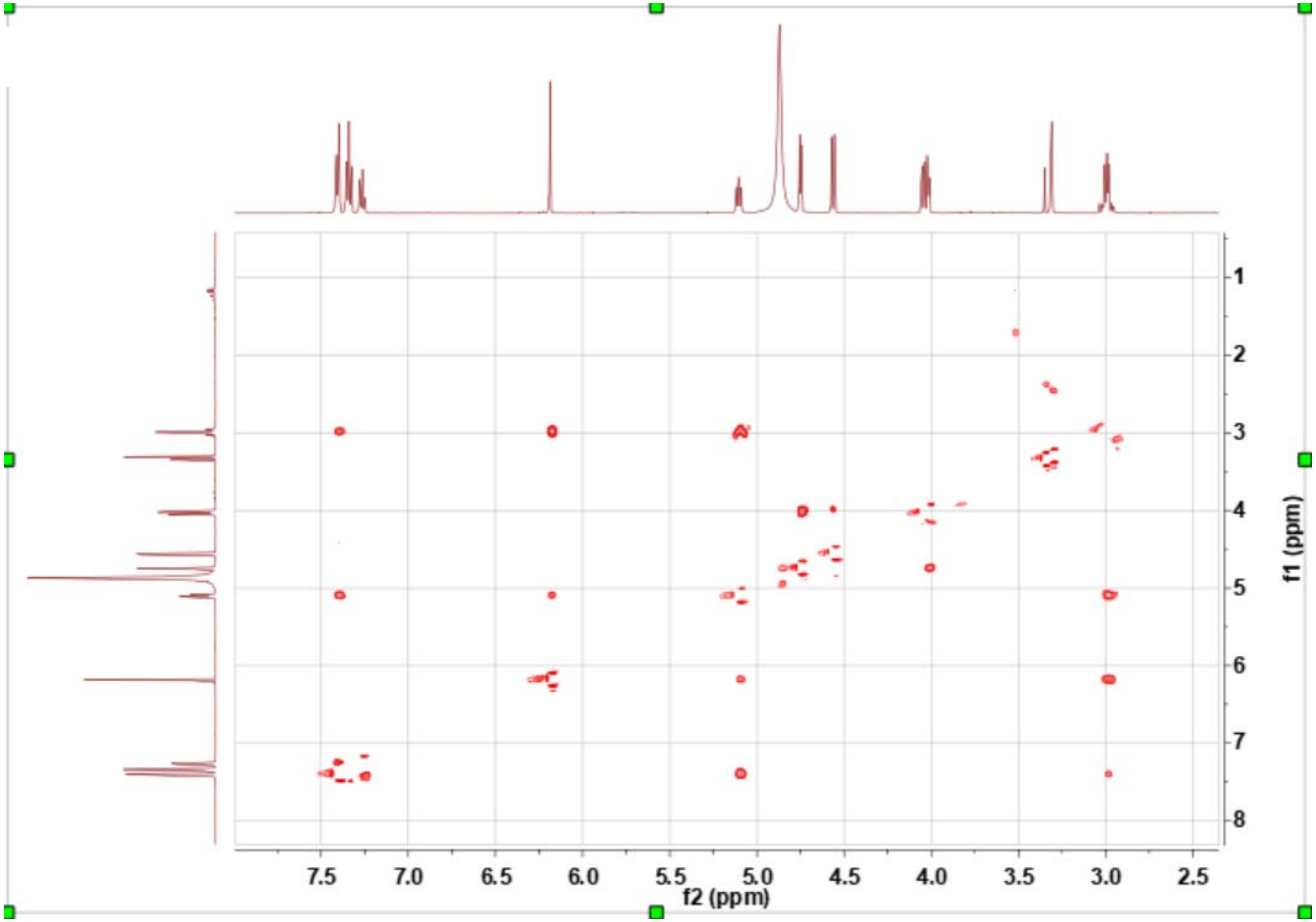

Fig. S9. HRESIMS spectrum of **1**.

Qualitative Analysis Report

**Data Filename**	190722ESIA1.d	**Sample Name**	pes8
**Sample Type**	Sample	**Position**	
**Instrument Name**	Agilent G6230 TOF MS	**User Name**	KIB
**Acq Method**	ESI.m	**Acquired Time**	7/22/2019 2:37:43 PM
**IRM Calibration Status**	Success	**DA Method**	ESI.m
**Comment**			

**Sample Group**		**Info.**
**Acquisition SW**	6200 series TOF/6500 series	
**Version**	Q-TOF B.05.01 (B5125.2)	

User Spectra

Fragmentor Voltage	Collision Energy	Ionization Mode
200	0	ESI

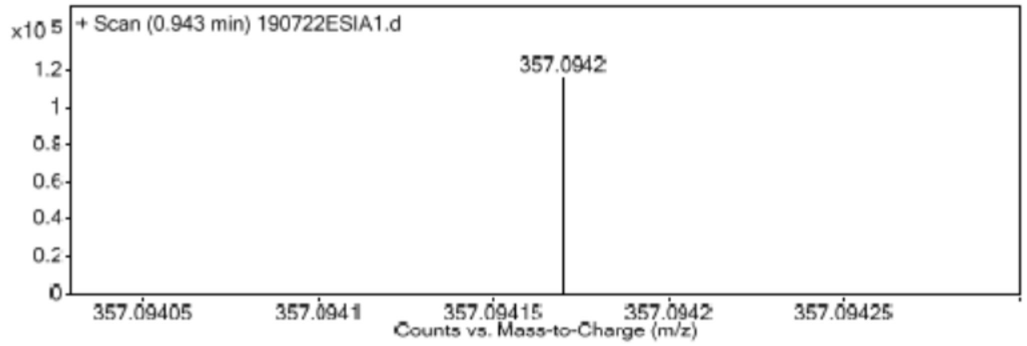

Peak List

m/z	z	Abund	Formula	Ion
121.0509	1	203239.73		
122.0551	1	24435.04		
293.1741	1	23895.3		
311.1823	1	21308.97		
357.0942	1	116030.03	C17 H18 Na O7	M+
358.0979	1	22161.25	C17 H18 Na O7	M+
398.1206	1	33174.25		
691.1995	1	77019.5		
692.2021	1	29713.47		
922.0098	1	79005.2		

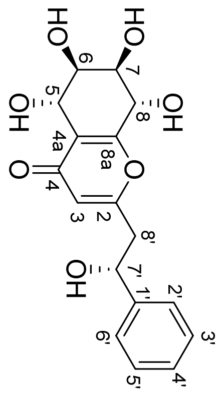

Formula Calculator Element Limits

Element	Min	Max
C	0	200
H	0	400
O	0	10
Na	1	1

Formula Calculator Results

Formula	CalculatedMass	Mz	Diff.(mDa)	Diff. (ppm)	DBE
C17 H18 Na O7	357.0950	357.0942	0.8	2.3	8.5

--- End Of Report ---

Fig. S10. ECD spectrum of **1**.

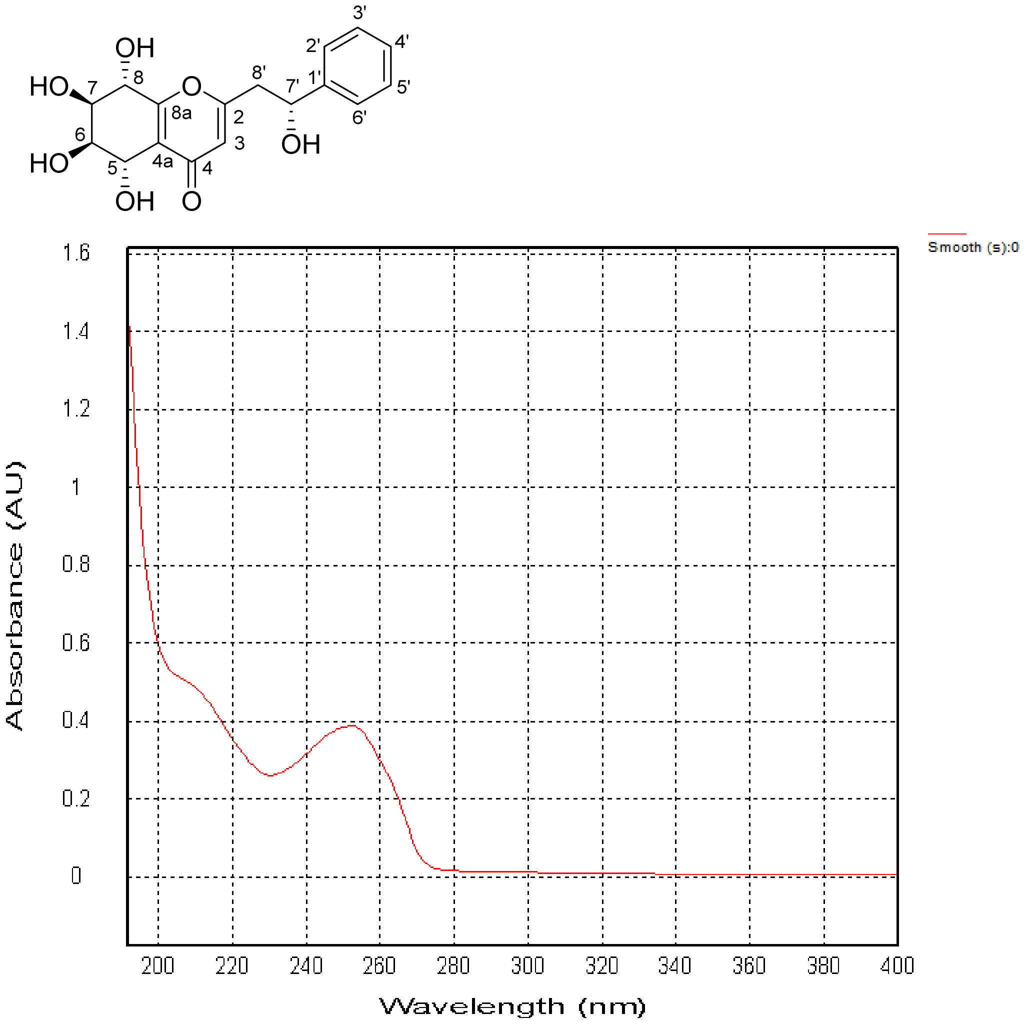

ProBinaryX

Attributes :

- Time Stamp :Fri Mar 29 13:39:12 2019

- File ID : {A2006467-9CCE-4a53-A374-15330326962E}
- Is CFR Compliant : false
- Original data has not been modified.

Remarks:

- User: CD
- Date: 2019/03/29
- Instrument: 0547
- DetectorType: LAAPD
- DichOS Calibration Correction Curve: 0547/2
- HV (CDDC channel): 0 v
- Time per point: 0.25 s
- Description: pes8
- Concentration: 0.13 mg/ml CH_3OH
- Pathlength: 1 mm
- Temperature: ---- C

Settings:

- Time-per-point: 0.25s (25us x 10000)
- SE
- Wavelength: 192nm - 400nm
- Step Size: 1nm
- Bandwidth: 1nm
- 3 repeats in set.
- -iter option selected

Fig. S11. ^{1}H NMR spectrum of **2** (methanol-d_4, 500 MHz).

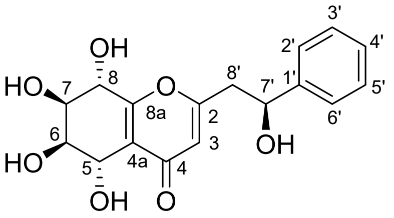

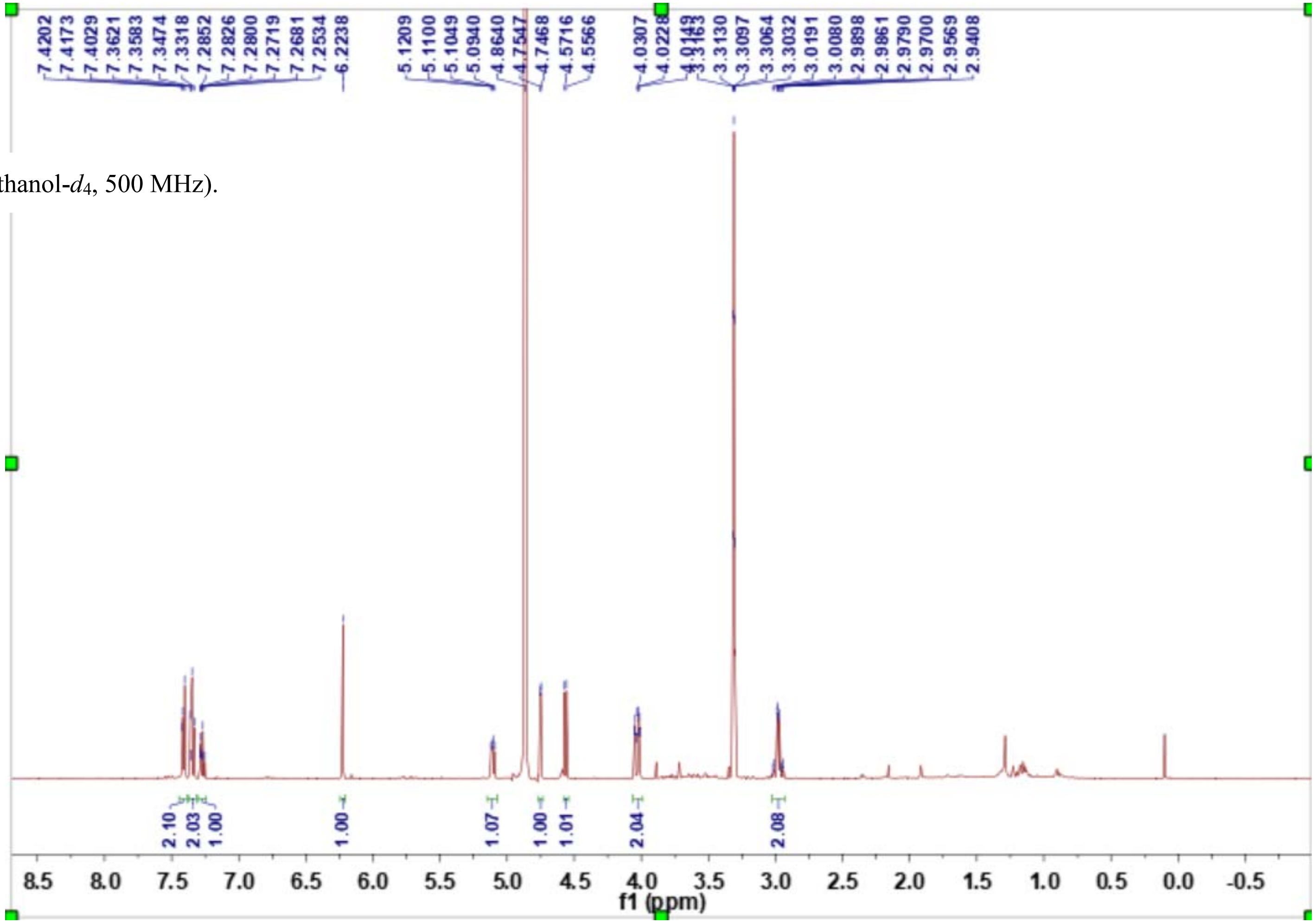

Fig. S12. ^{13}C NMR spectrum of **2** (methanol-d_4, 126 MHz).

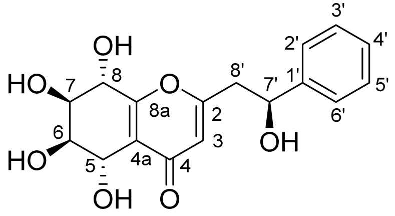

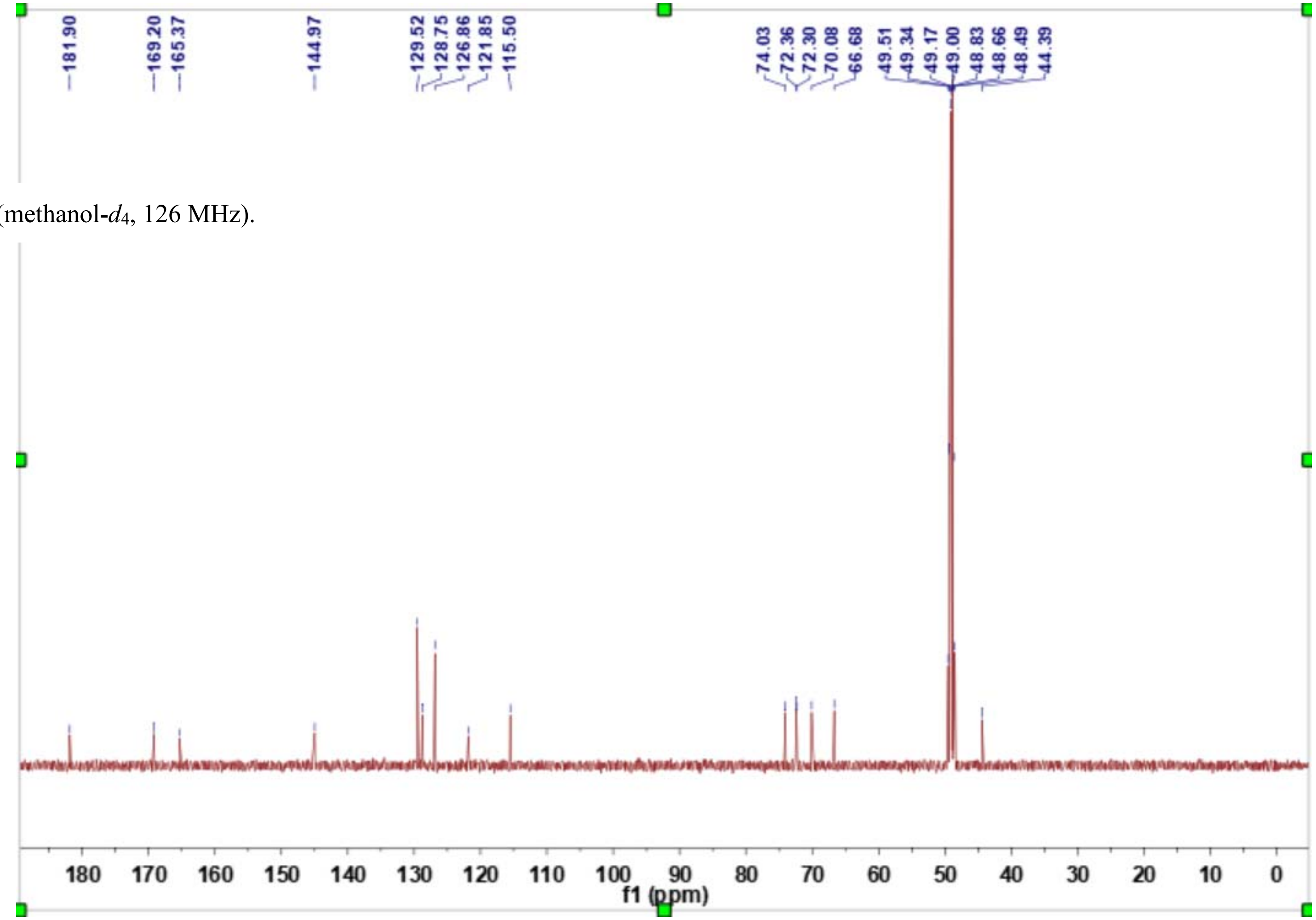

Fig. S13. HSQC spectrum of **2**.

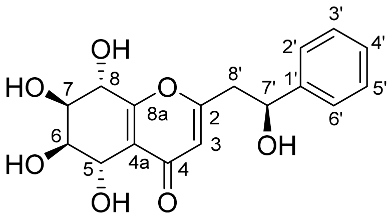

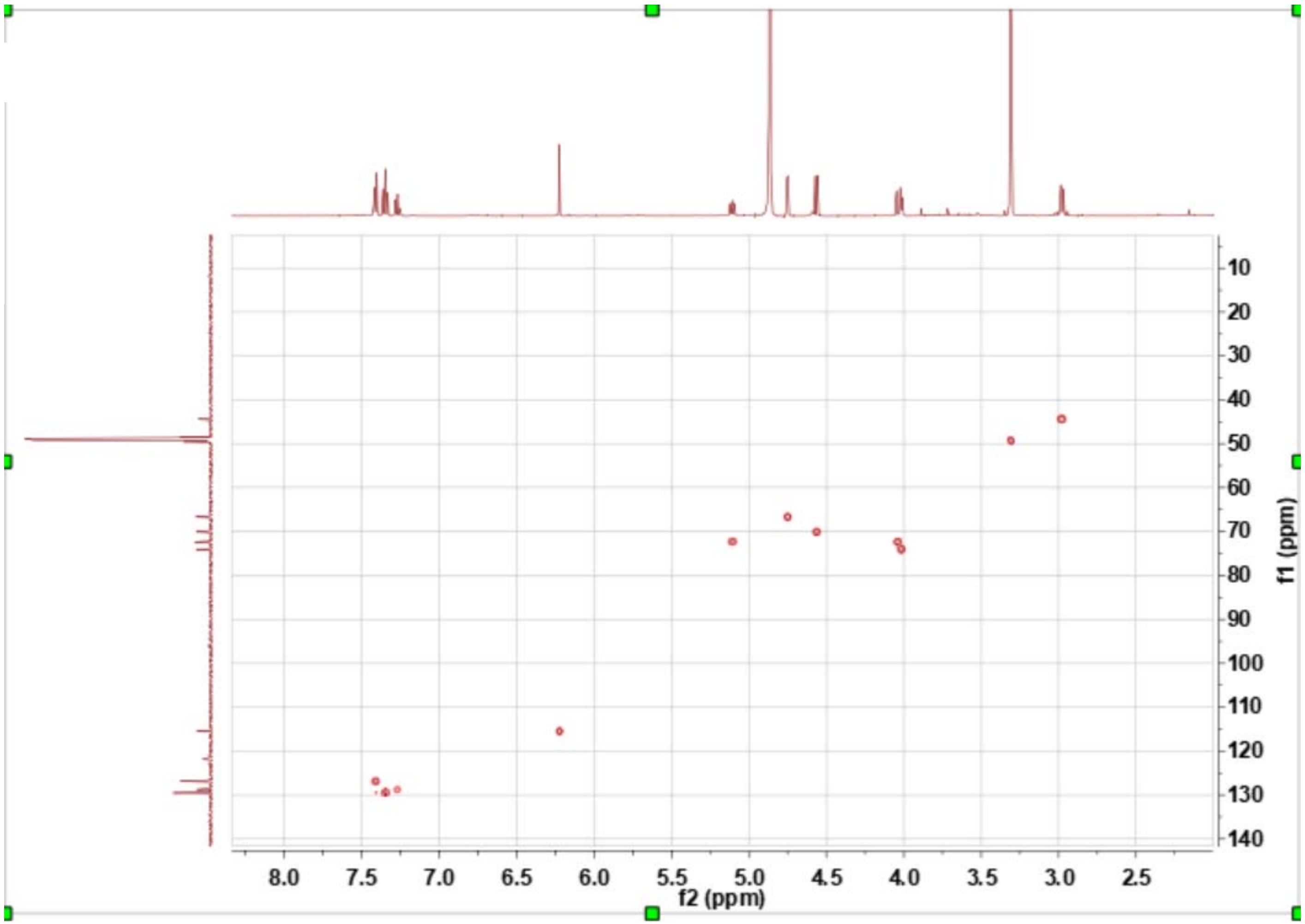

Fig. S14. ^{1}H–^{1}H COSY spectrum of **2**.

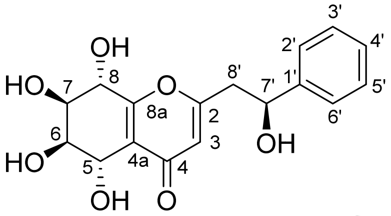

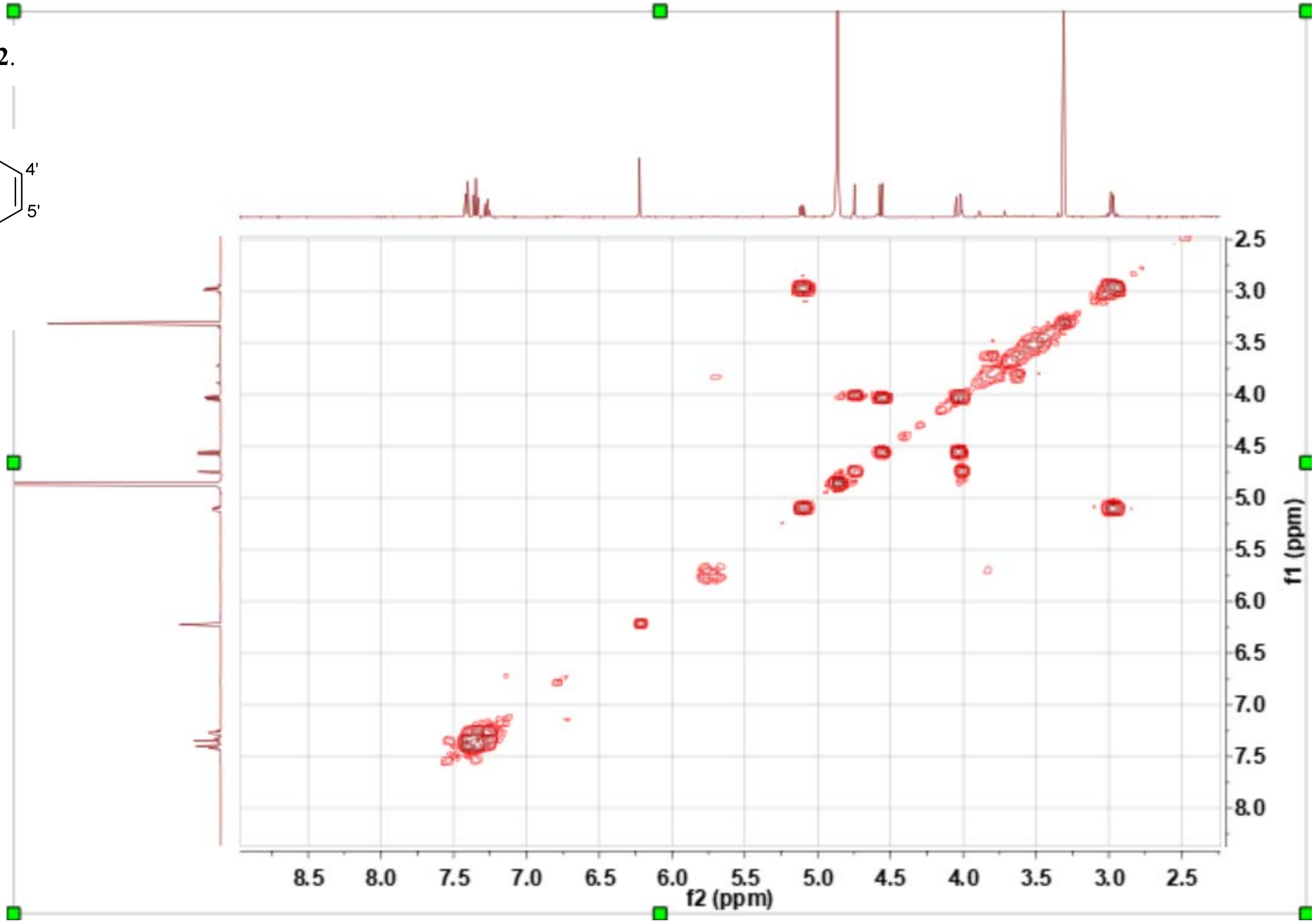

Fig. S15. HMBC spectrum of **2**.

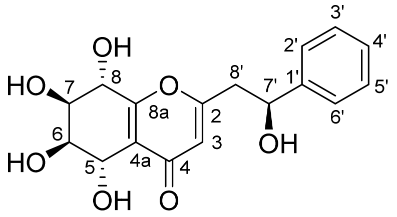

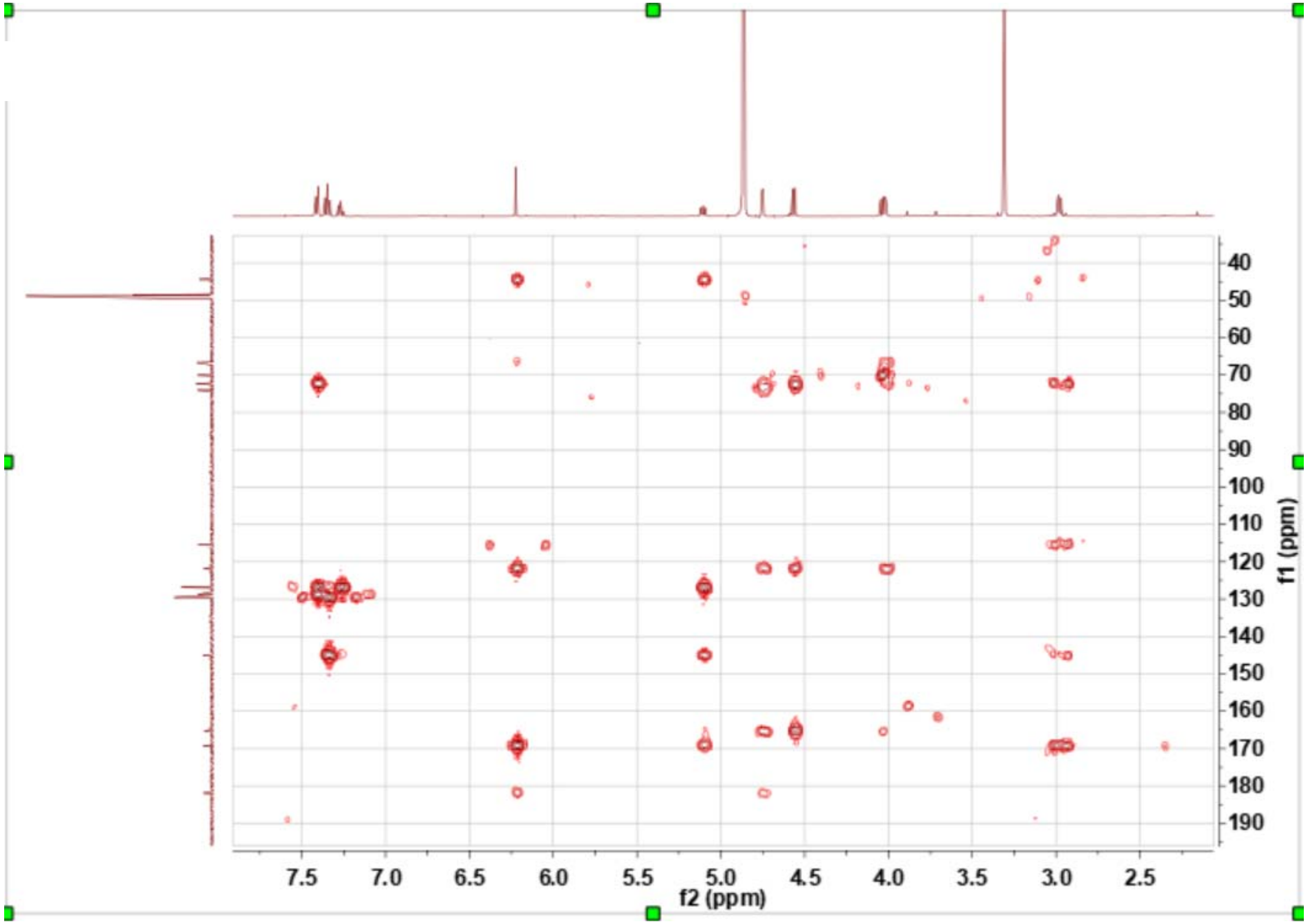

Fig. S16. ROESY spectrum of **2**.

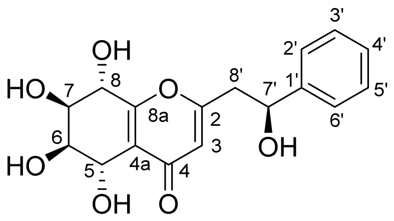

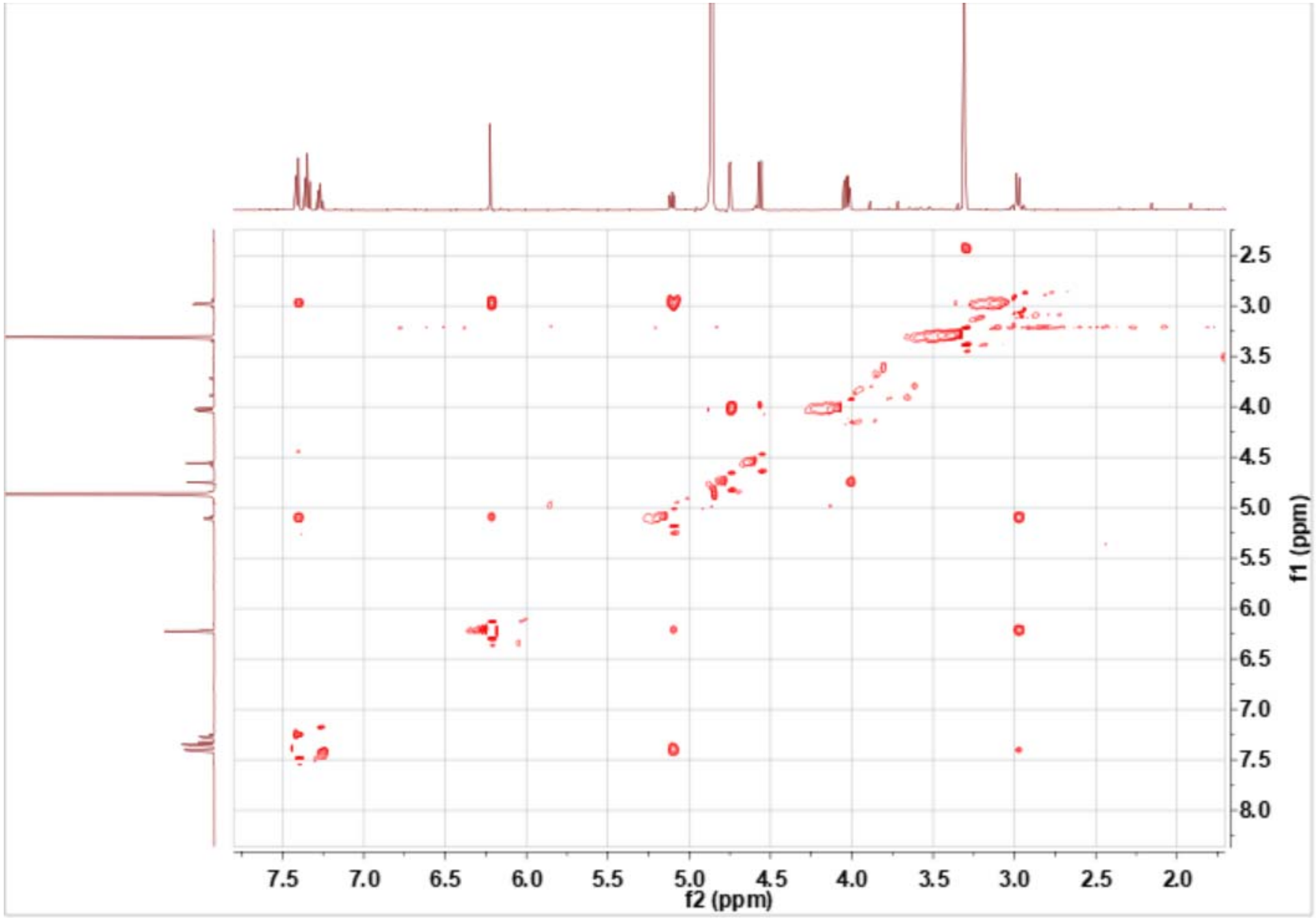

Fig. S17. HRESIMS spectrum of **2**.

Qualitative Analysis Report

**Data Filename**	190722ESIA2.d	**Sample Name**	pes9
**Sample Type**	Sample	**Position**	
**Instrument Name**	Agilent G6230 TOF MS	**User Name**	KIB
**Acq Method**	ESI.m	**Acquired Time**	7/22/2019 2:39:45 PM
**IRM Calibration Status**	Success	**DA Method**	ESI.m
**Comment**			

**Sample Group**		**Info.**
**Acquisition SW**	6200 series TOF/6500 series	
**Version**	Q-TOF B.05.01 (B5125.2)	

User Spectra

Fragmentor Voltage	Collision Energy	Ionization Mode
200	0	ESI

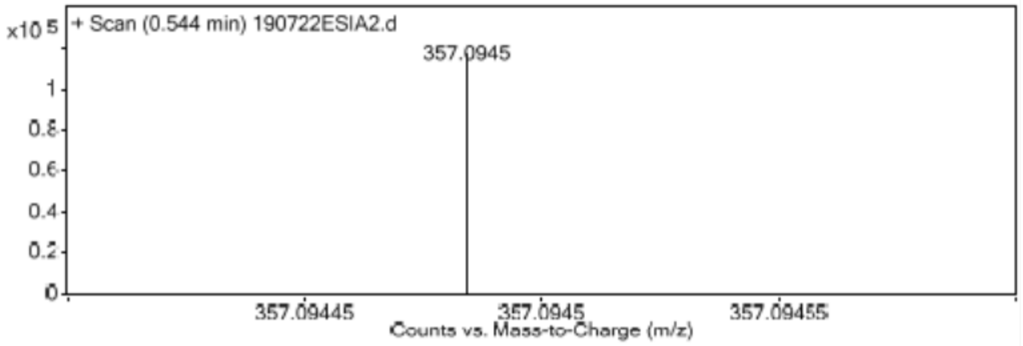

Peak List

m/z	z	Abund	Formula	Ion
121.0509	1	83357.57		
311.1821	1	45893.13		
333.11	1	24320.54		
357.0945	1	116727.62	C17 H18 Na O7	M+
373.0684	1	25141.26		
398.1207	1	36471.59		
521.1388	2	21234.15		
691.1998	1	77843.66		
692.2031	1	28780.44		
922.0098	1	39696.55		

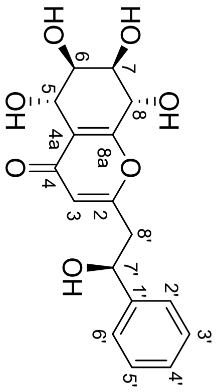

Formula Calculator Element Limits

Element	Min	Max
C	0	200
H	0	400
O	0	10
Na	1	1

Formula Calculator Results

Formula	CalculatedMass	Mz	Diff.(mDa)	Diff. (ppm)	DBE
C17 H18 Na O7	357.0950	357.0945	0.5	1.5	8.5

--- End Of Report ---

Fig. S18. ECD spectrum of **2**.

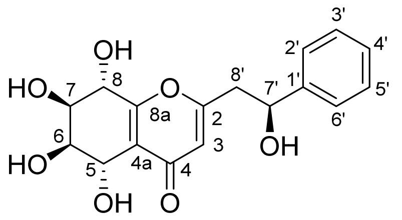

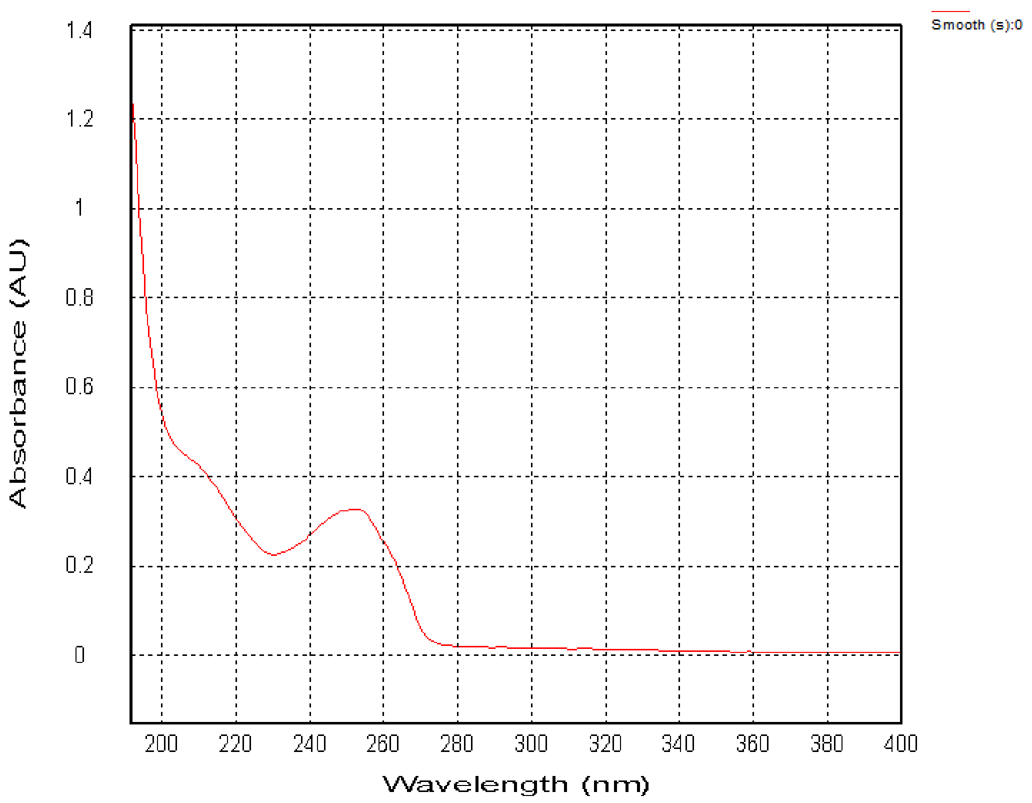

ProBinaryX
Attributes :
- Time Stamp :Fri Mar 29 13:45:48 2019

- File ID : {88E60AA6-76F2-4ea6-B147-442D95BD3288}
- Is CFR Compliant : false
- Original data has not been modified.

Remarks:
- User: CD
- Date: 2019/03/29
- Instrument: 0547
- DetectorType: LAAPD
- DichOS Calibration Correction Curve: 0547/2
- HV (CDDC channel): 0 v
- Time per point: 0.25 s
- Description: pes9
- Concentration: 0.175 mg/ml CH_3OH
- Pathlength: 1 mm
- Temperature: ---- C

Settings:
- Time-per-point: 0.25s (25us x 10000)
- SE
- Wavelength: 192nm - 400nm
- Step Size: 1nm
- Bandwidth: 1nm
- 3 repeats in set.
- -iter option selected

Fig. S19. ^{1}H NMR spectrum of **3** (methanol-d_4, 600 MHz).

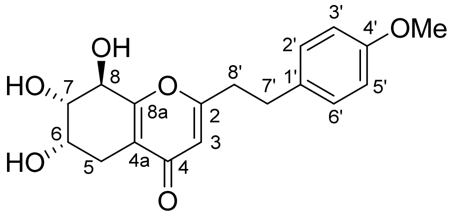

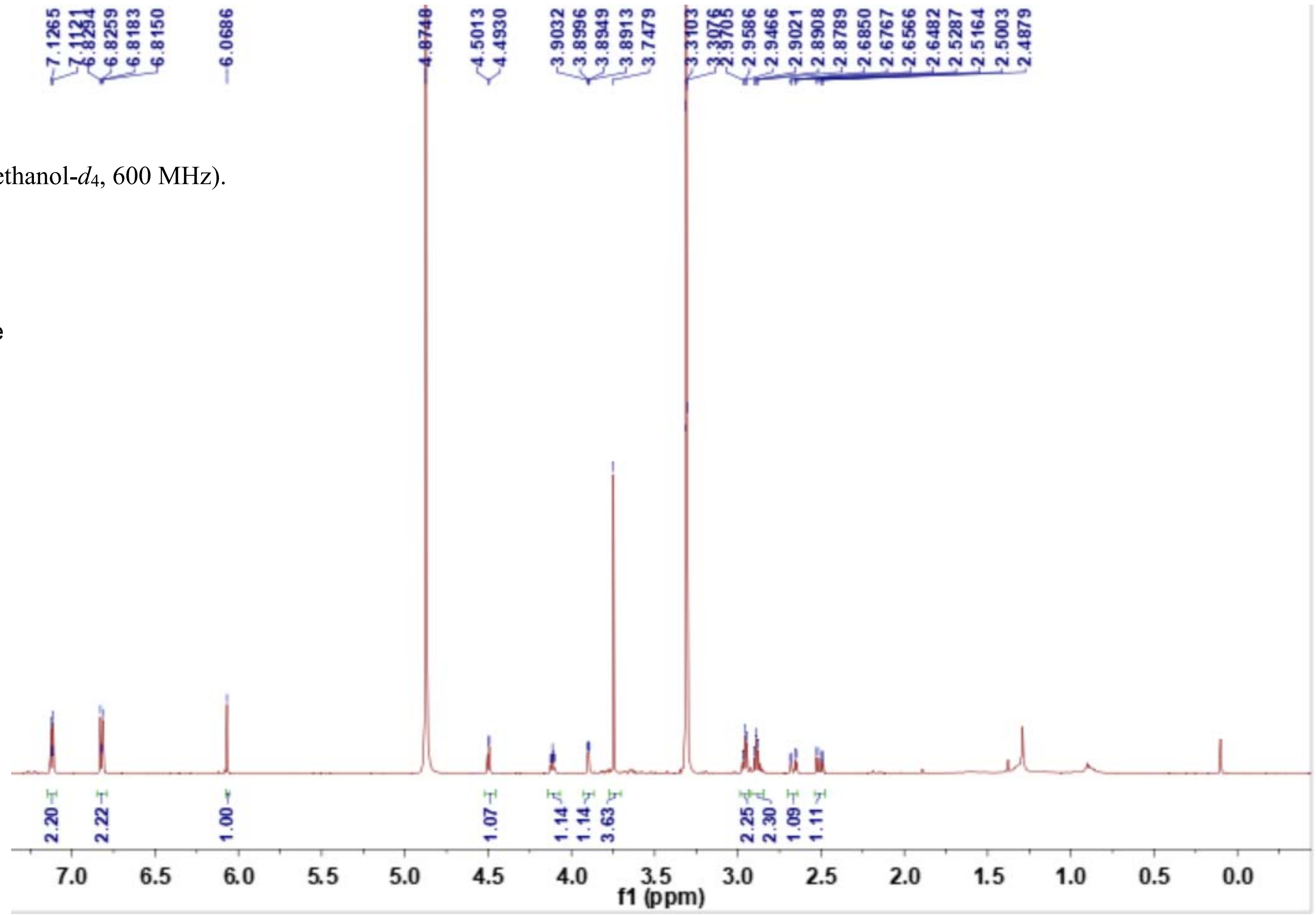

Fig. S20. ^{13}C NMR spectrum of **3** (methanol-d_4, 151 MHz).

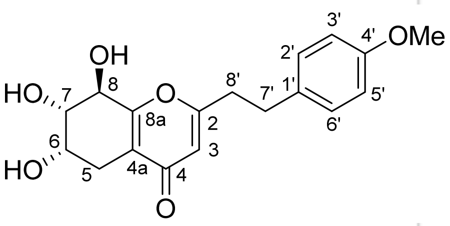

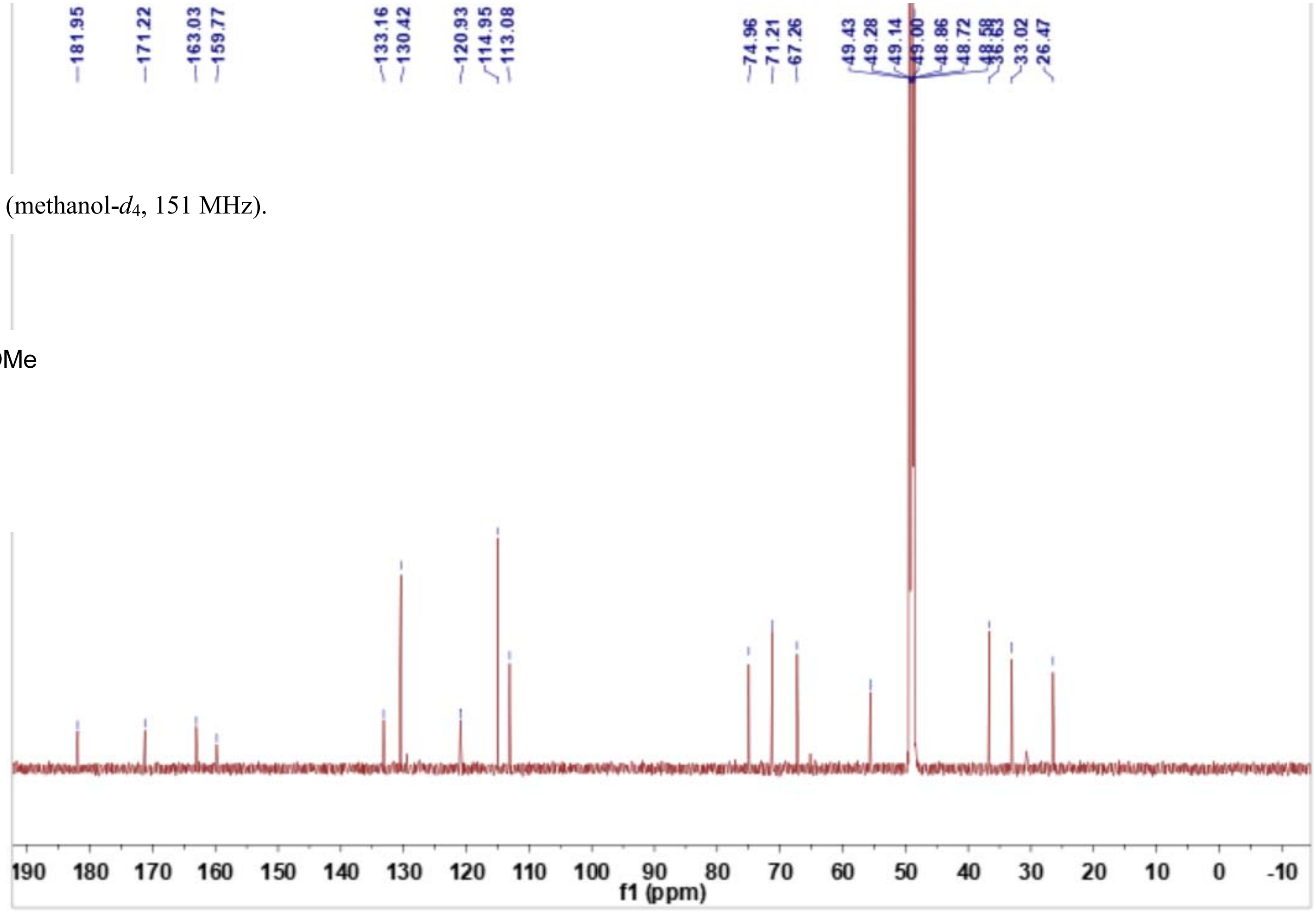

Fig. S21. HSQC spectrum of **3**.

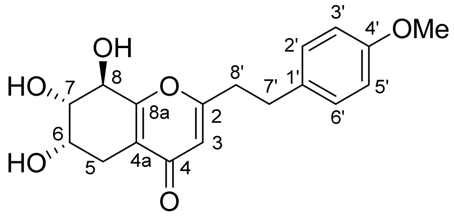

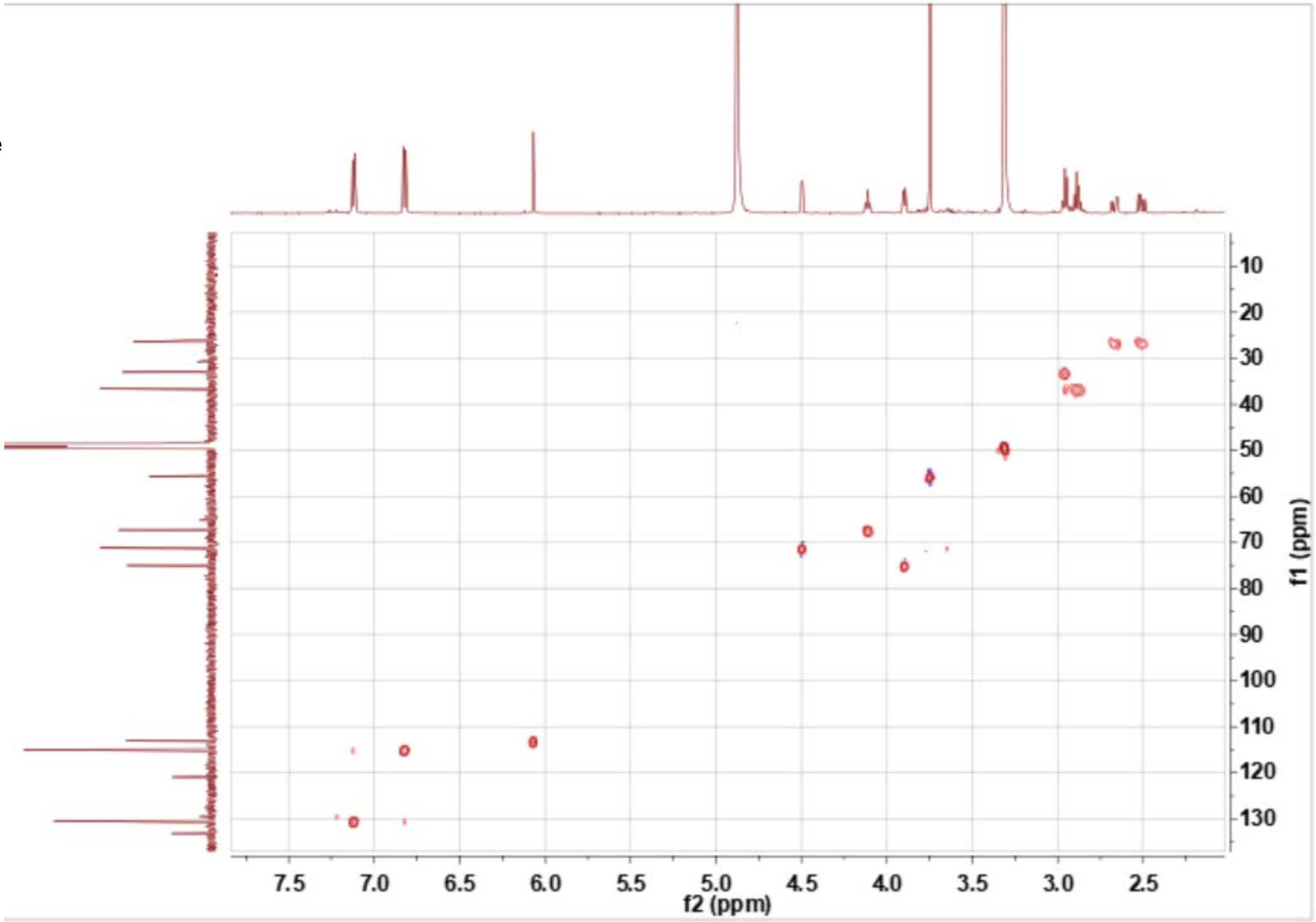

Fig. S22. ^1H–^1H COSY spectrum of **3**.

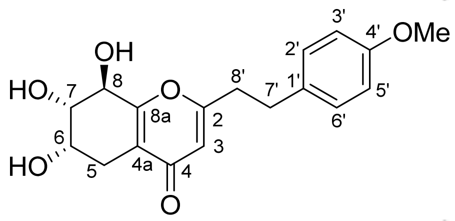

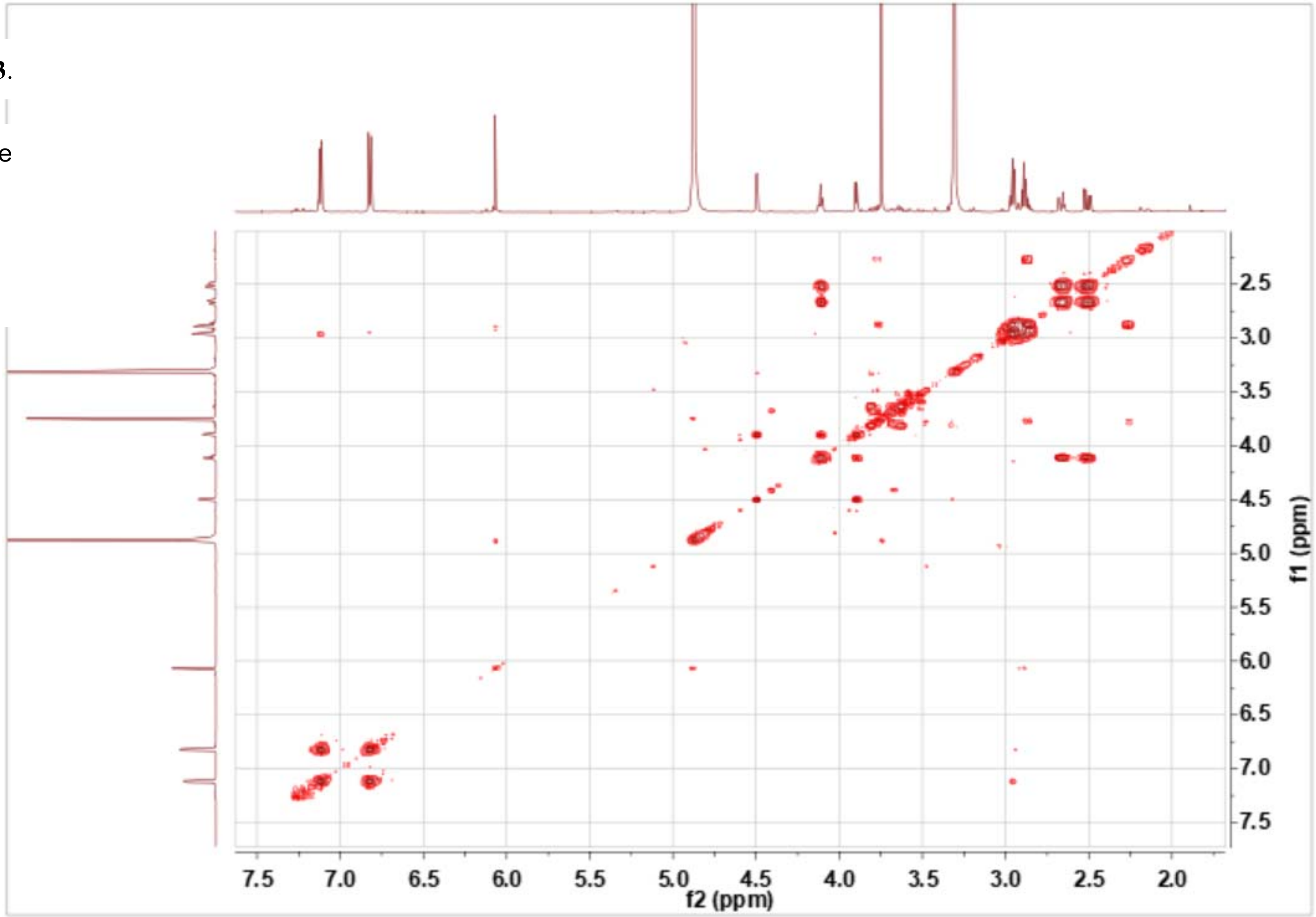

Fig. S23. HMBC spectrum of **3**.

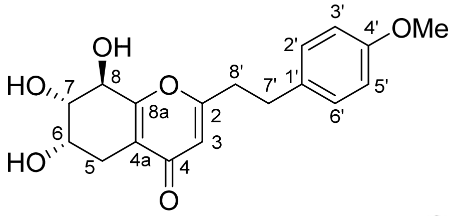

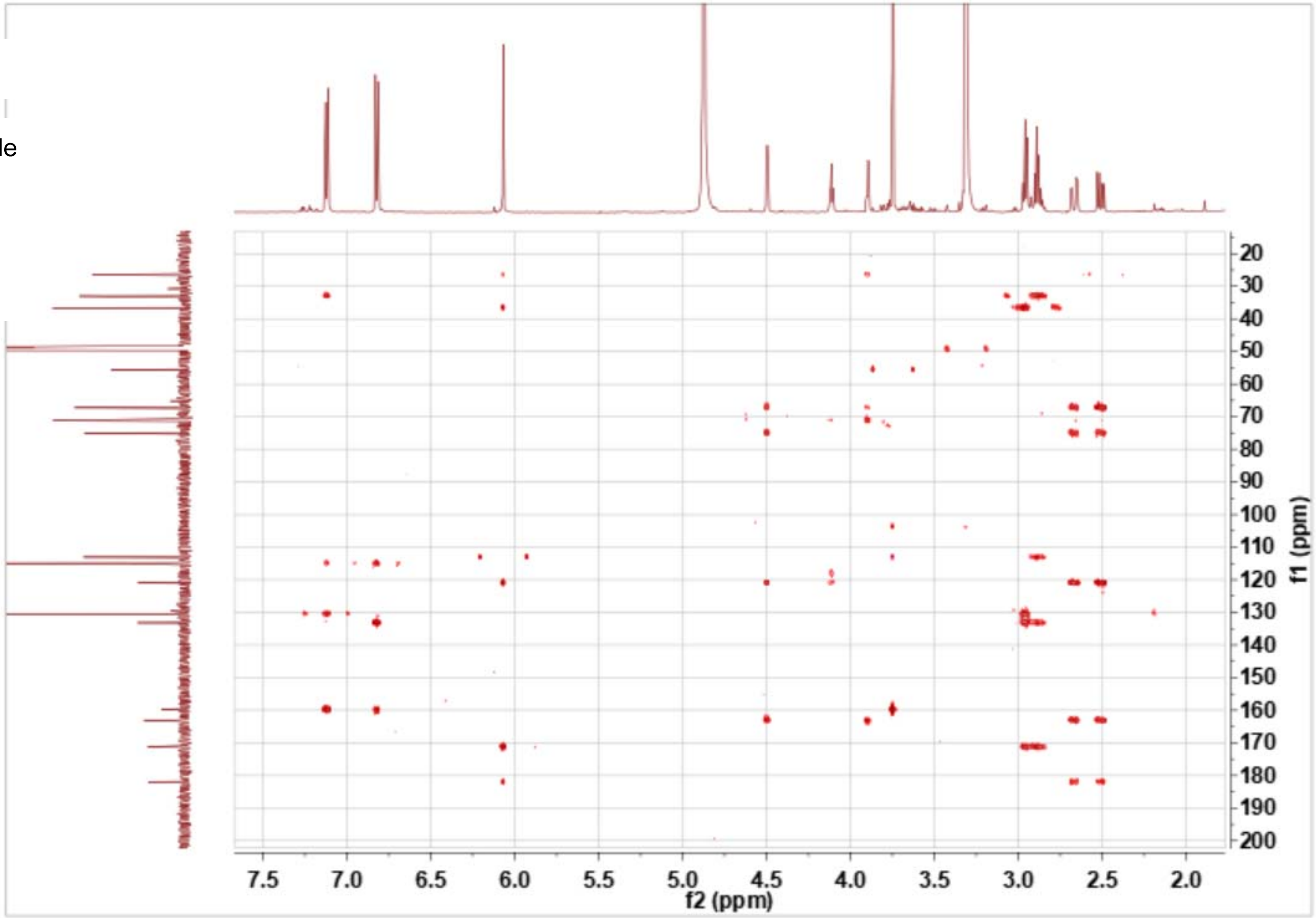

Fig. S24. ROESY spectrum of **3**.

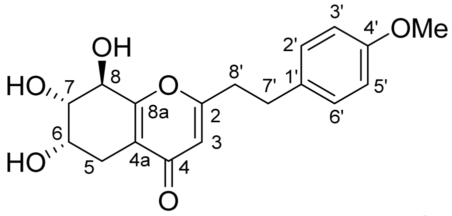

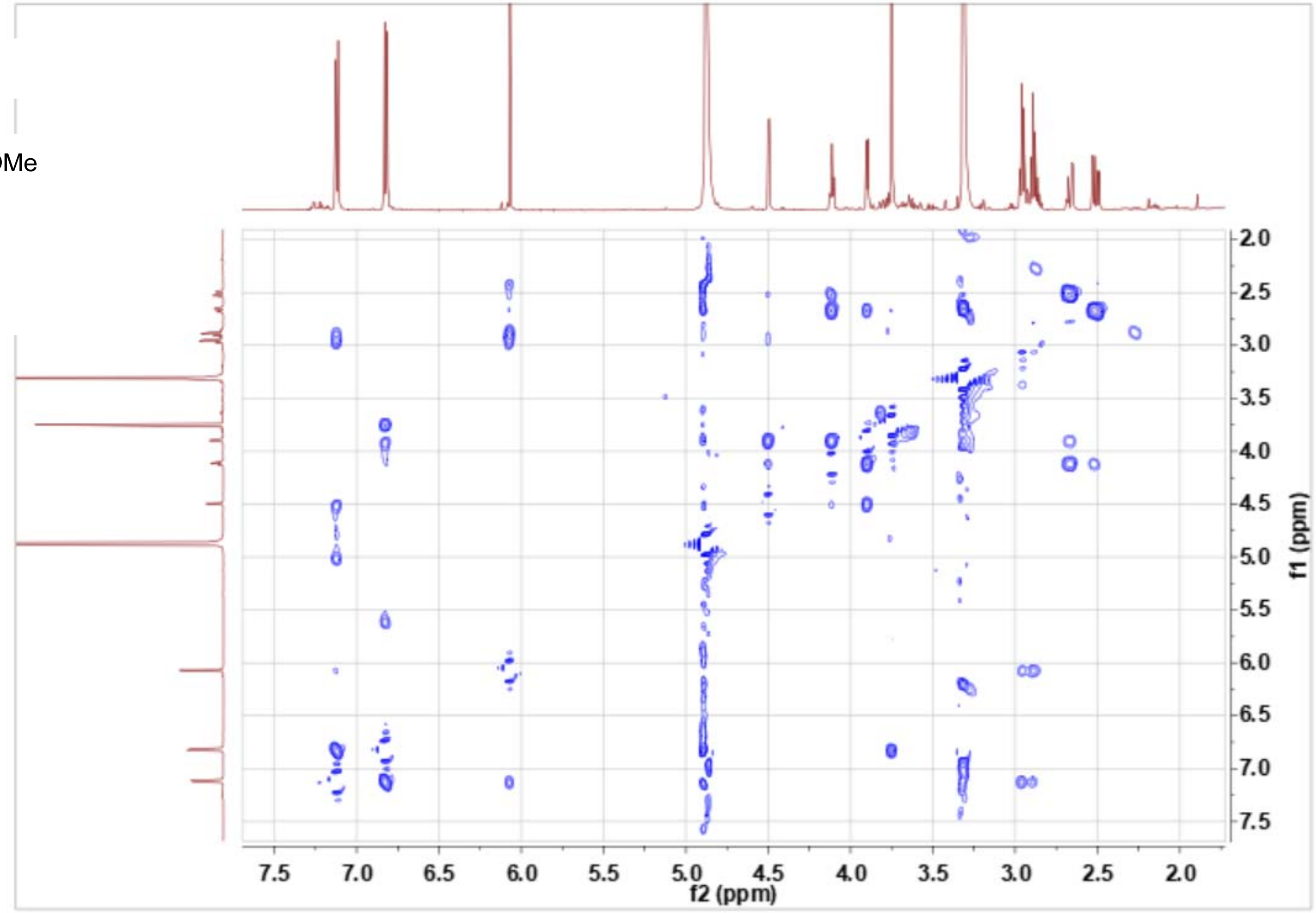

Fig. S25. HRESIMS spectrum of 3.

Qualitative Analysis Report

**Data Filename**	190722ESIA4.d	**Sample Name**	pes21
**Sample Type**	Sample	**Position**	
**Instrument Name**	Agilent G6230 TOF MS	**User Name**	KIB
**Acq Method**	ESI.m	**Acquired Time**	7/22/2019 2:42:49 PM
**IRM Calibration Status**	Success	**DA Method**	ESI.m
**Comment**			

Sample Group | **Info.**
Acquisition SW Version 6200 series TOF/6500 series Q-TOF B.05.01 (B5125.2)

User Spectra

Fragmentor Voltage	Collision Energy	Ionization Mode
200	0	ESI

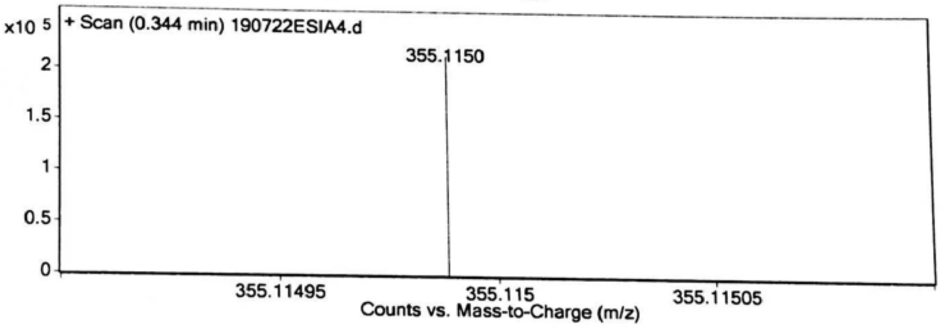

Peak List

m/z	z	Abund	Formula	Ion
102.1276		31981.33		
121.0509	1	247749.39		
122.0555	1	30285.31		
293.1739	1	25359.54		
355.115	1	214460.2	C18 H20 Na O6	M+
356.1181	1	40208.53	C18 H20 Na O6	M+
396.1405	1	23391.71		
687.2406	1	76241.02		
688.244	1	31913.13		
922.0098	1	103730.59		

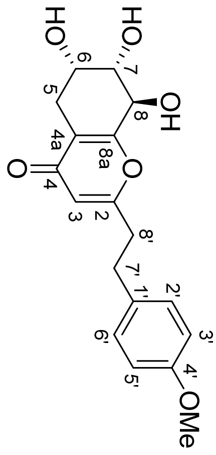

Formula Calculator Element Limits

Element	Min	Max
C	0	200
H	0	400
O	0	10
Na	1	1

Formula Calculator Results

Formula	CalculatedMass	Mz	Diff.(mDa)	Diff. (ppm)	DBE
C18 H20 Na O6	355.1158	355.1150	0.8	2.1	8.5

--- End Of Report ---

Fig. S26. ECD spectrum of **3**.

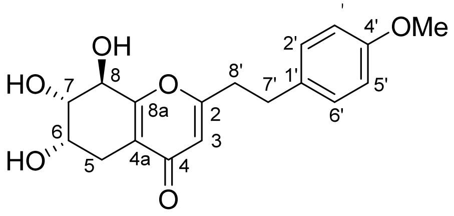

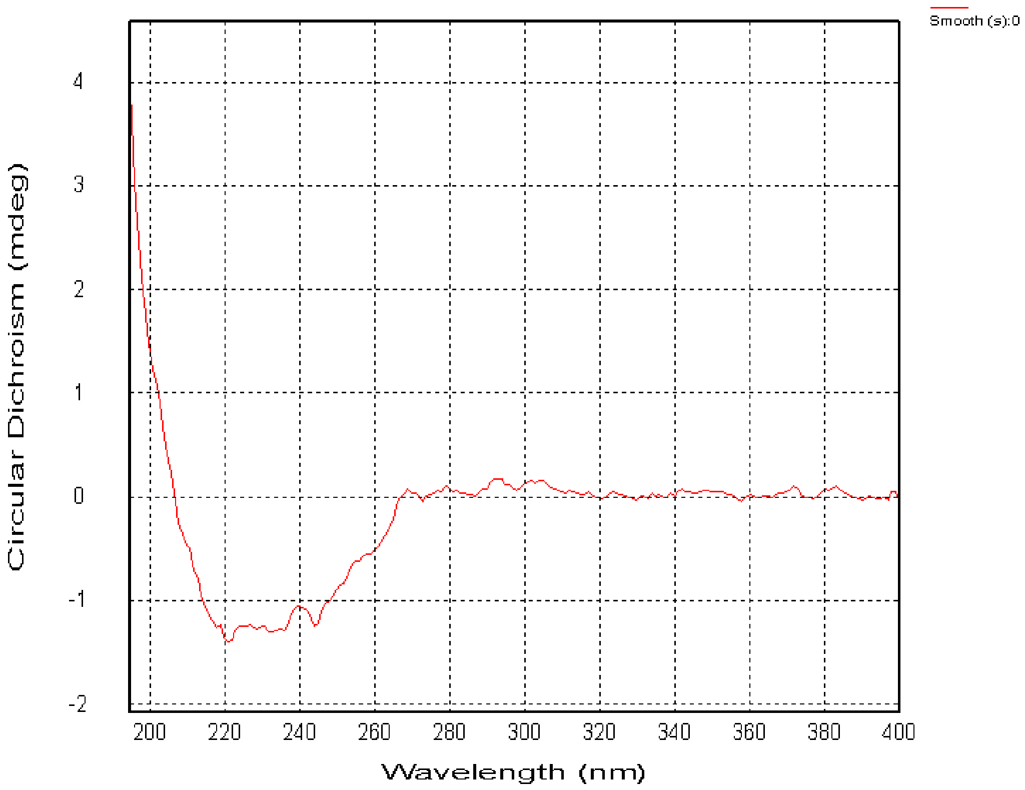

File: CD PES21-1mm (195-400)19092306.dsx
ProBinaryX
Attributes :
- Time Stamp :Mon Sep 23 18:51:30 2019

- File ID : {F8628197-CDD3-4925-9F54-DA9C79A52B43}
- Is CFR Compliant : false
- Original data has not been modified.

Remarks:
- User: APLService
- Date: 2019/09/23
- Instrument: 0218
- DetectorType: PMT
- DichOS Calibration Correction Curve: 0218/1
- HV (CDDC channel): 0 v
- Time per point: 1 s
- Description: Sample 1
- Concentration: 0.0994mg/mL MeOH
- Pathlength: 1 mm
- Temperature: 20℃

Settings:
- Time-per-point: 1s (25us x 40000)
- SE
- Wavelength: 195nm - 400nm
- Step Size: 1nm
- Bandwidth: 1nm

Fig. S27. ^{1}H NMR spectrum of **4** (methanol-d_4, 500 MHz).

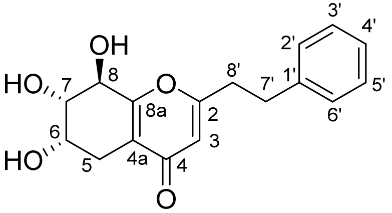

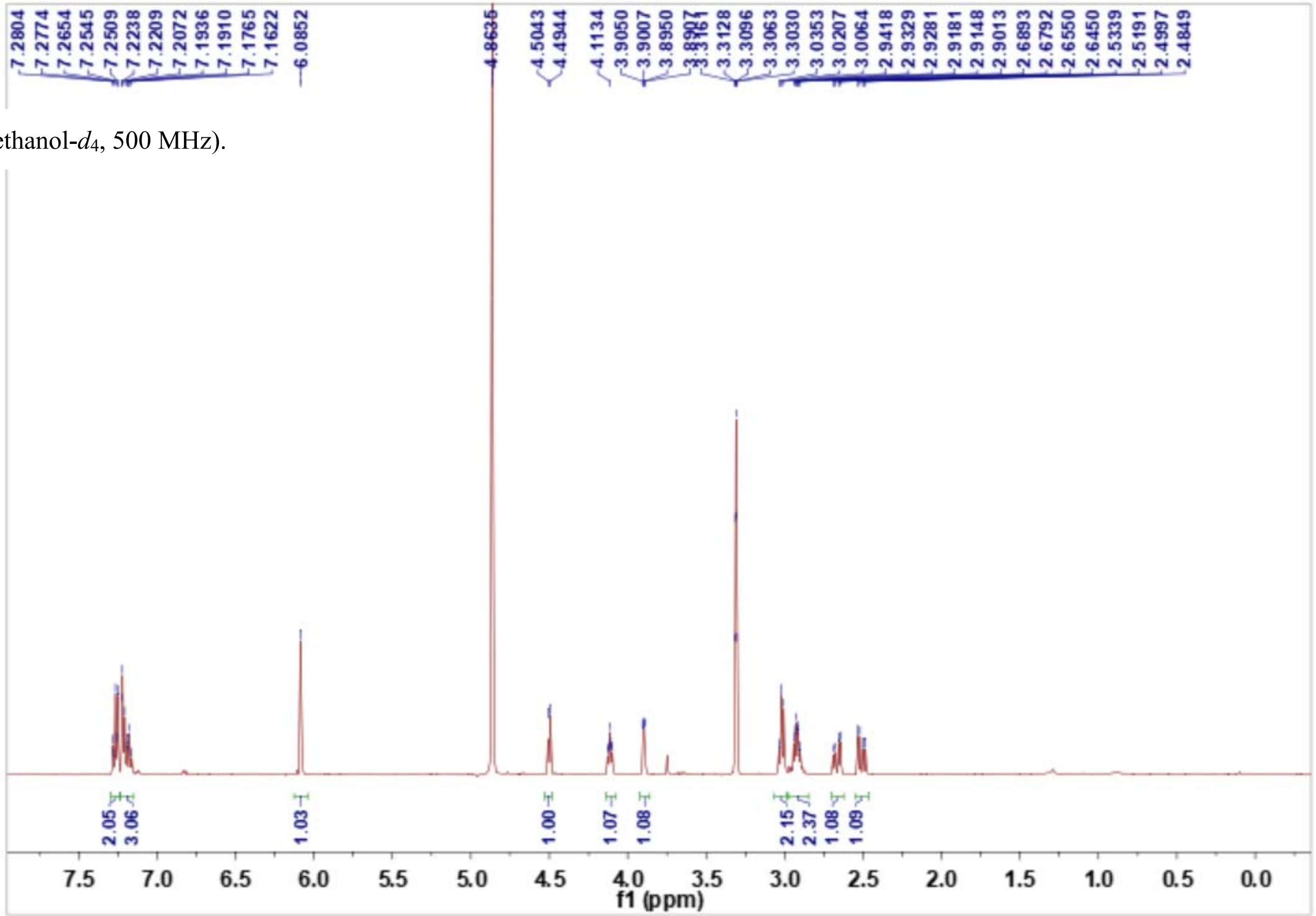

Fig. S28. ^{13}C NMR spectrum of **4** (methanol-d_4, 126 MHz).

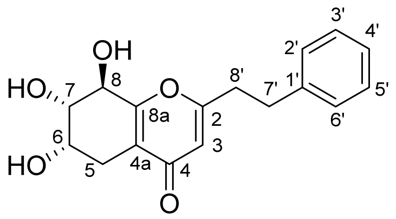

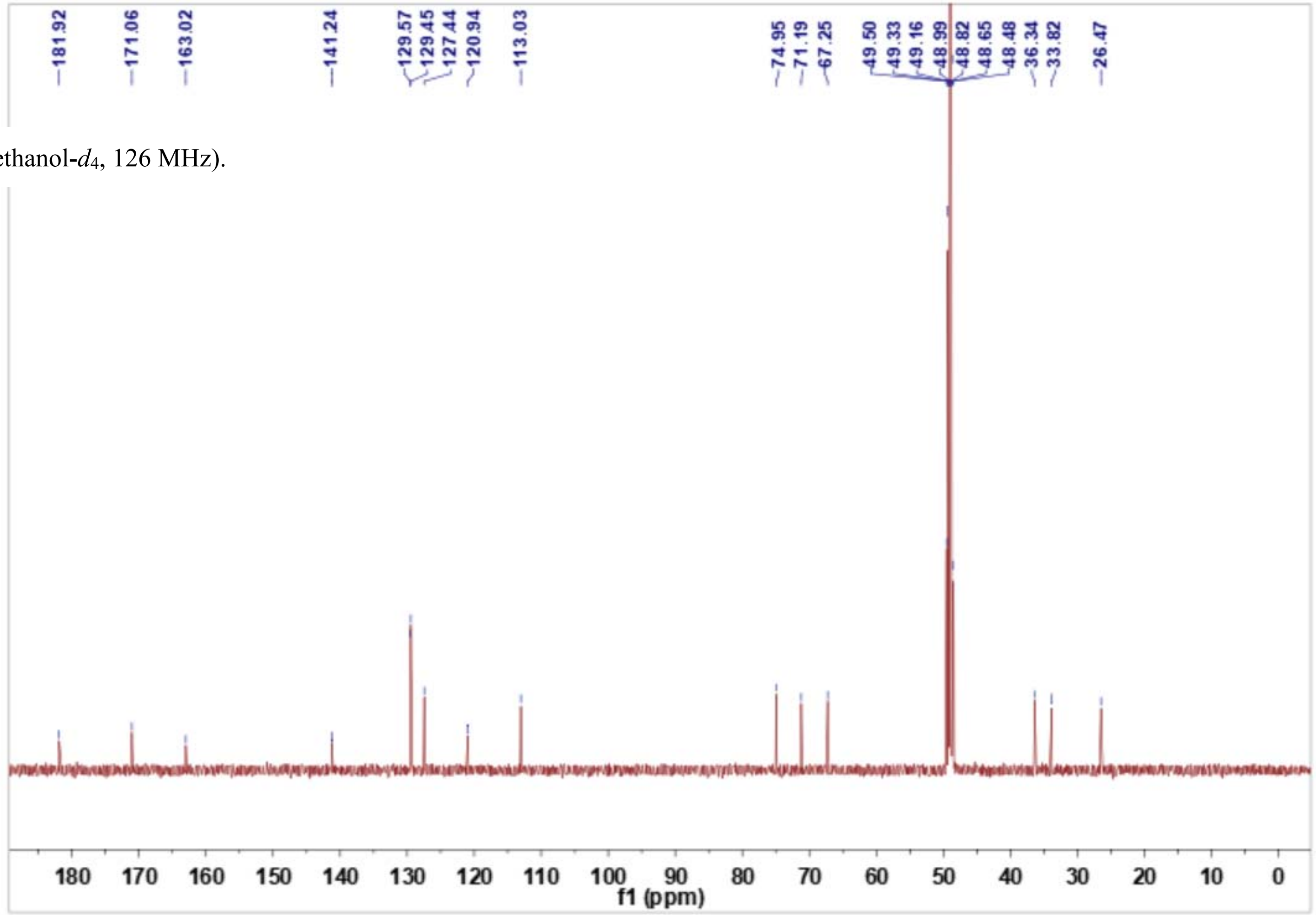

Fig. S29. HSQC spectrum of **4**.

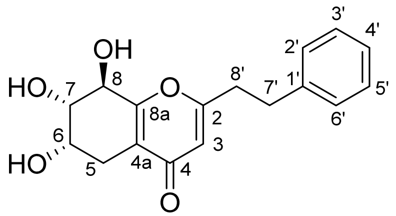

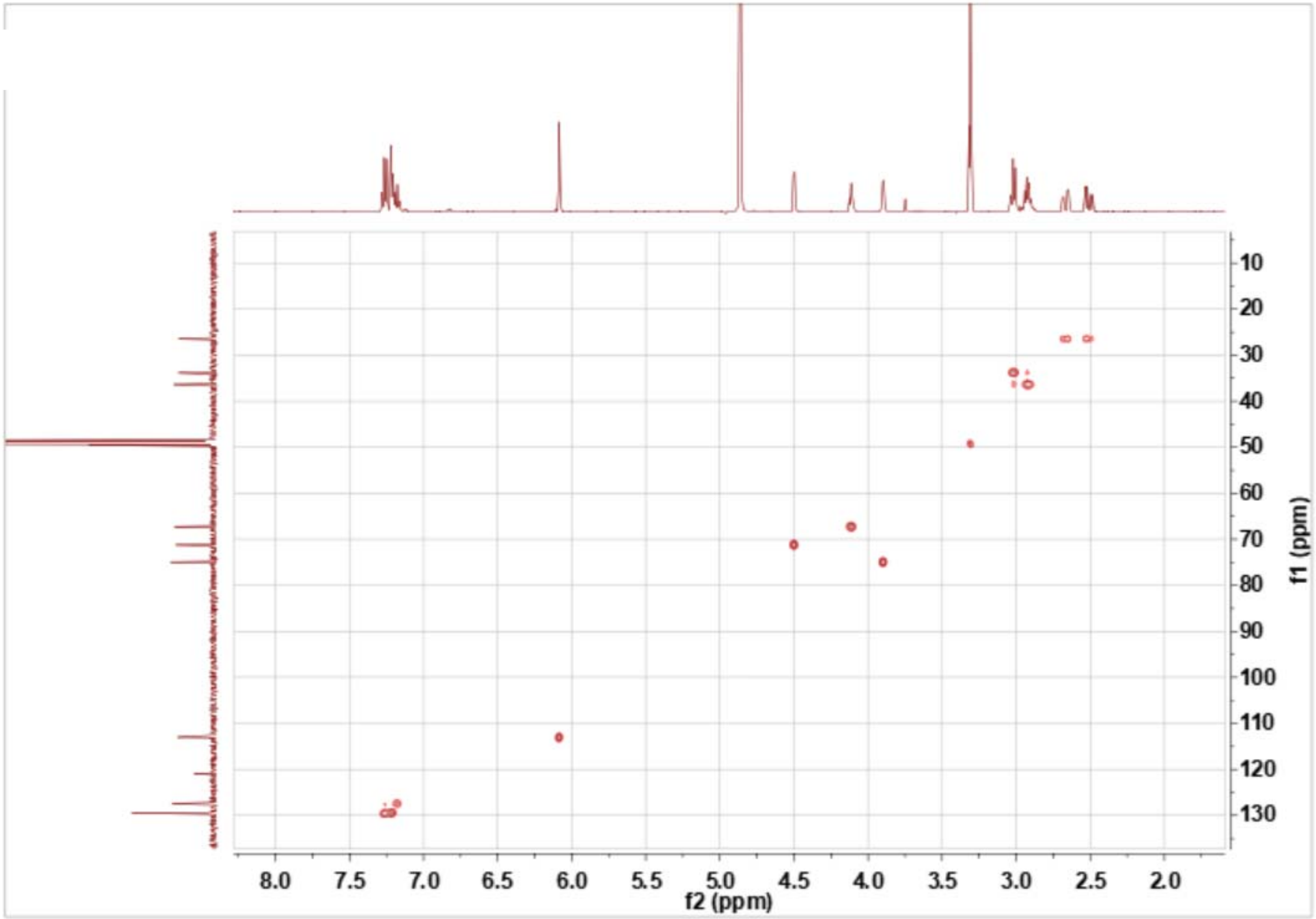

Fig. S30. ^1H–^1H COSY spectrum of **4**.

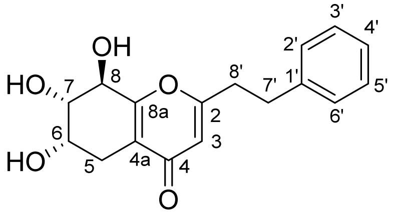

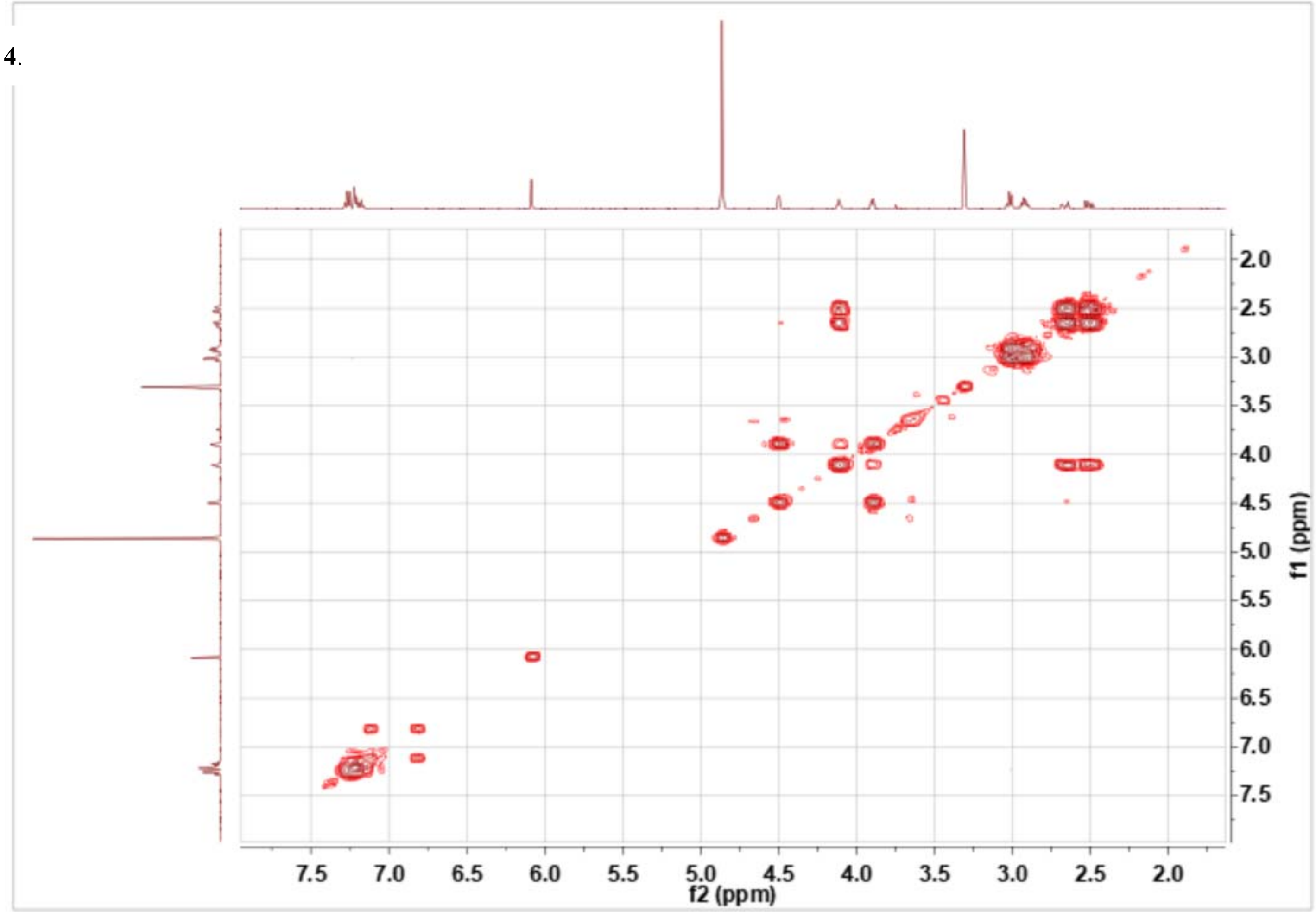

Fig. S31. HMBC spectrum of **4**.

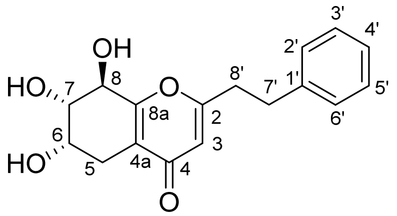

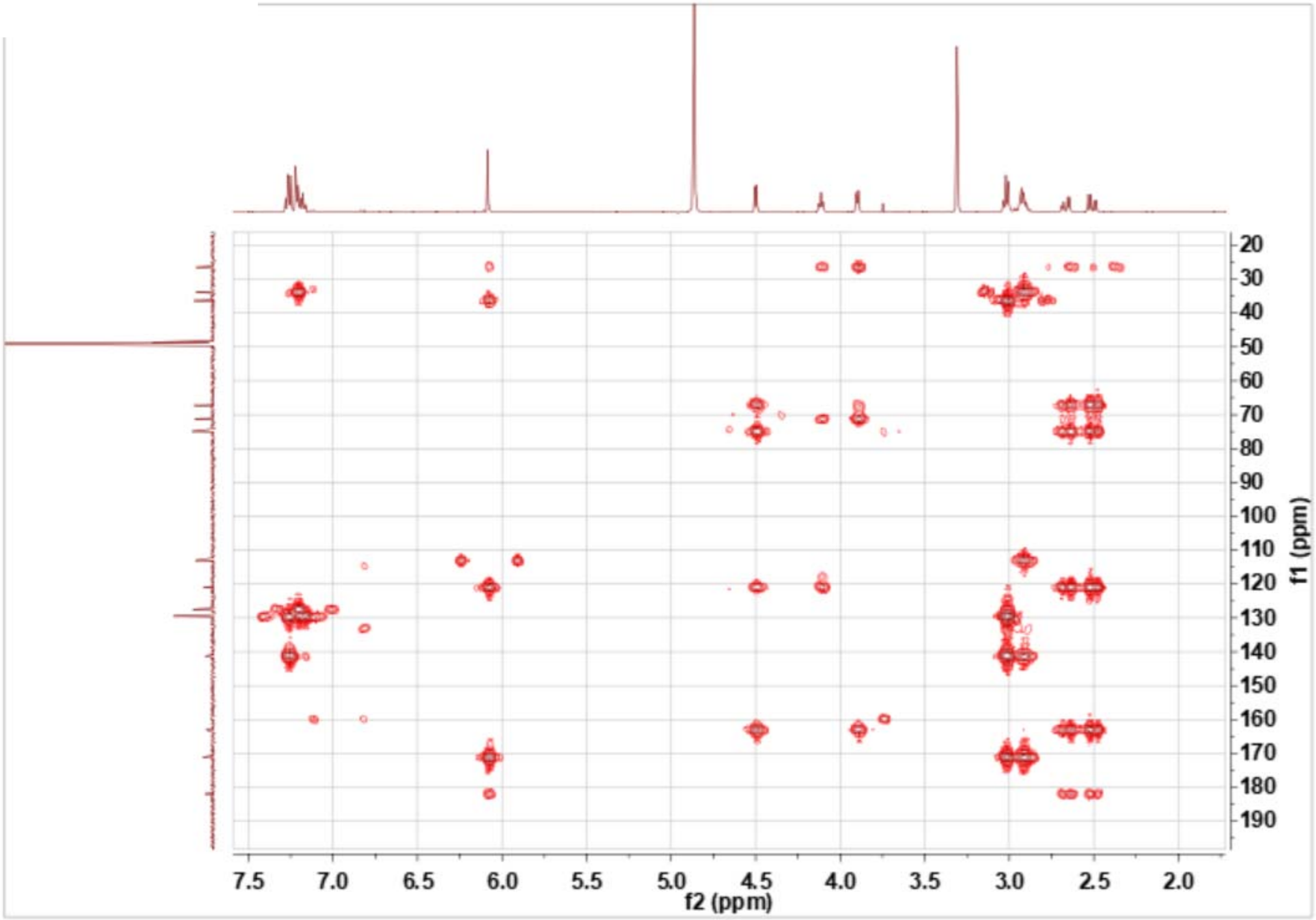

Fig. S32. ROESY spectrum of **4**.

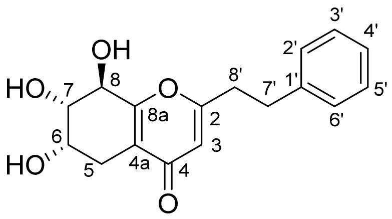

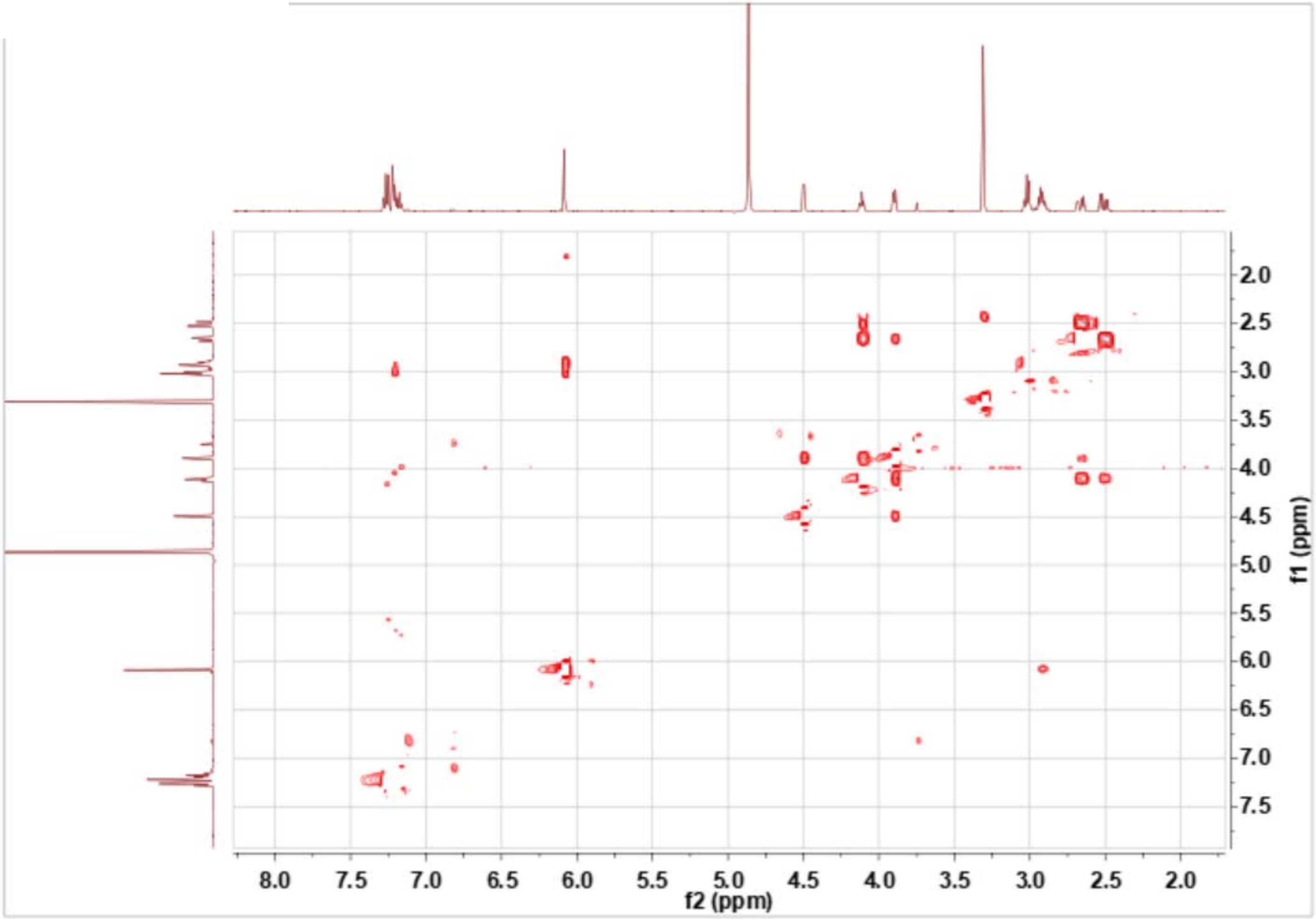

Fig. S33. HRESIMS spectrum of **4**.

Qualitative Analysis Report

**Data Filename**	190722ESIA3.d	**Sample Name**	pes19
**Sample Type**	Sample	**Position**	
**Instrument Name**	Agilent G6230 TOF MS	**User Name**	KIB
**Acq Method**	ESI.m	**Acquired Time**	7/22/2019 2:41:20 PM
**IRM Calibration Status**	Success	**DA Method**	ESI.m
**Comment**			

Sample Group **Info.**

Acquisition SW Version 6200 series TOF/6500 series Q-TOF B.05.01 (B5125.2)

User Spectra

Fragmentor Voltage	Collision Energy	Ionization Mode
200	0	ESI

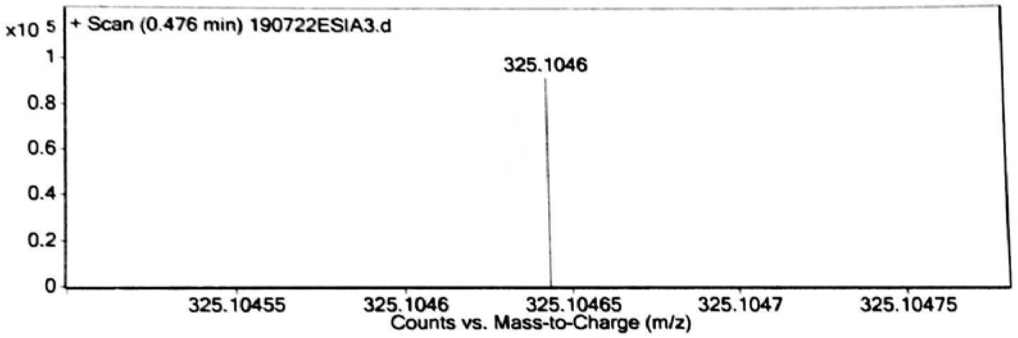

Peak List

m/z	z	Abund	Formula	Ion
121.0509	1	70124.66		
303.1226	1	34721.67		
325.1046	1	92083.17	C17 H18 Na O5	M+
366.1315	1	182561.53		
367.1339	1	41816.7		
627.2208	1	244796.11		
628.2238	1	90836.45		
922.0098	1	30757.37		
929.3353	1	116803.5		
930.3393	1	62667.83		

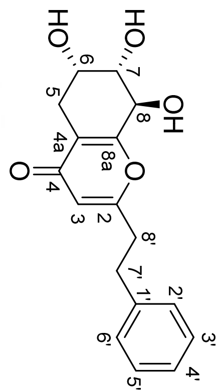

Formula Calculator Element Limits

Element	Min	Max
C	0	200
H	0	400
O	0	10
Na	1	1

Formula Calculator Results

Formula	CalculatedMass	Mz	Diff.(mDa)	Diff. (ppm)	DBE
C17 H18 Na O5	325.1052	325.1046	0.6	1.8	8.5

--- End Of Report ---

Fig. S34. ECD spectrum of **4**.

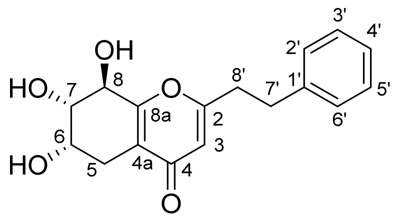

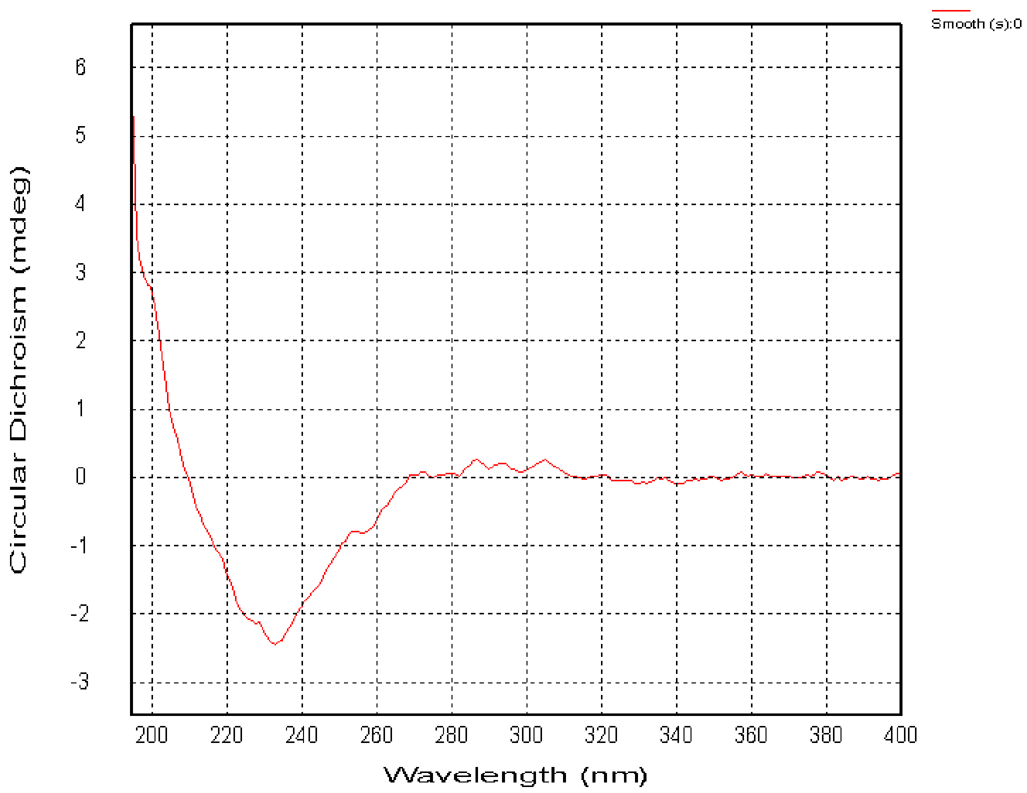

File: CD PES19-1mm (195-400)19080108.dsx
ProBinaryX
Attributes :
- Time Stamp :Thu Aug 01 19:47:33 2019

- File ID : {926B7B33-47BB-4e4c-BE82-8472FD9AAAB1}
- Is CFR Compliant : false
- Original data has not been modified.

Remarks:
- User: APLService
- Date: 2019/08/01
- Instrument: 0218
- DetectorType: PMT
- DichOS Calibration Correction Curve: 0218/1
- HV (CDDC channel): 0 v
- Time per point: 1 s
- Description: Sample 1
- Concentration: 0.0720mg/mL MeOH
- Pathlength: 1 mm
- Temperature: 20℃

Settings:
- Time-per-point: 1s (25us x 40000)
- SE
- Wavelength: 195nm - 400nm
- Step Size: 1nm
- Bandwidth: 1nm

Fig. S35. ^{1}H NMR spectrum of **5** (methanol-d_4, 600 MHz).

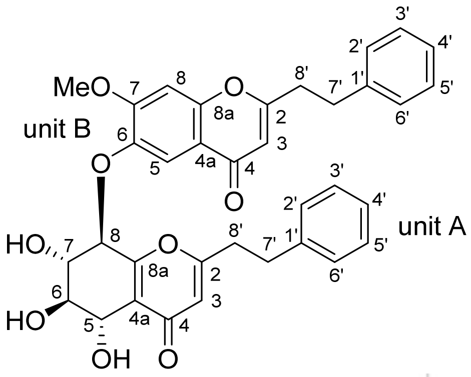

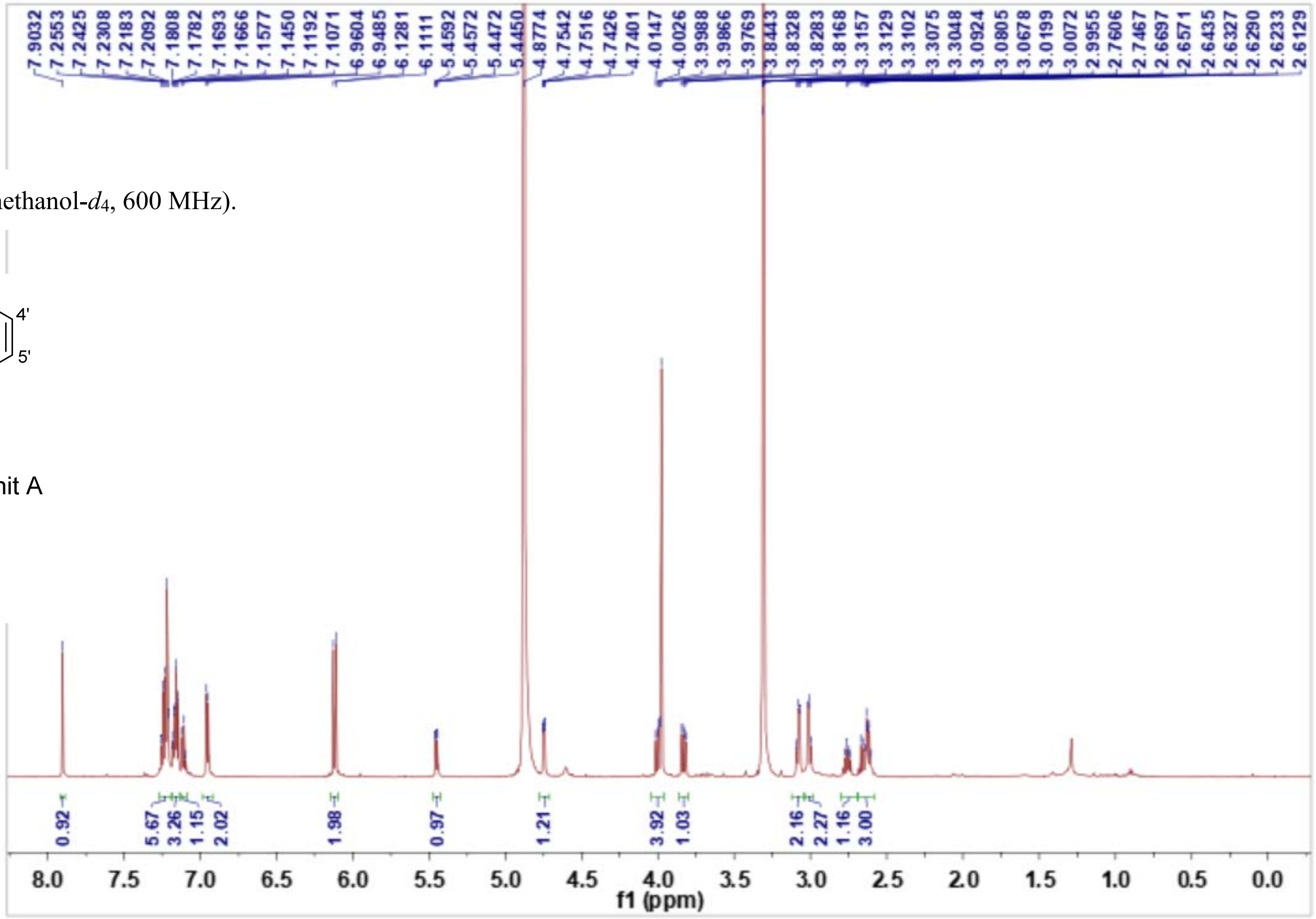

Fig. S36. ^{13}C NMR spectrum of **5** (methanol-d_4, 151 MHz).

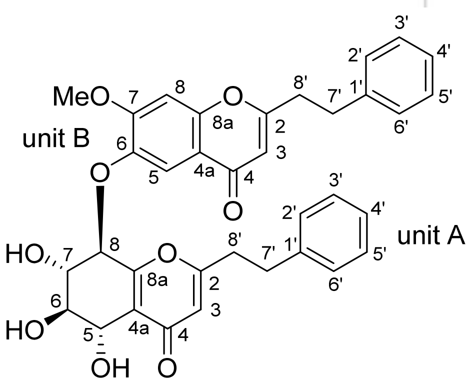

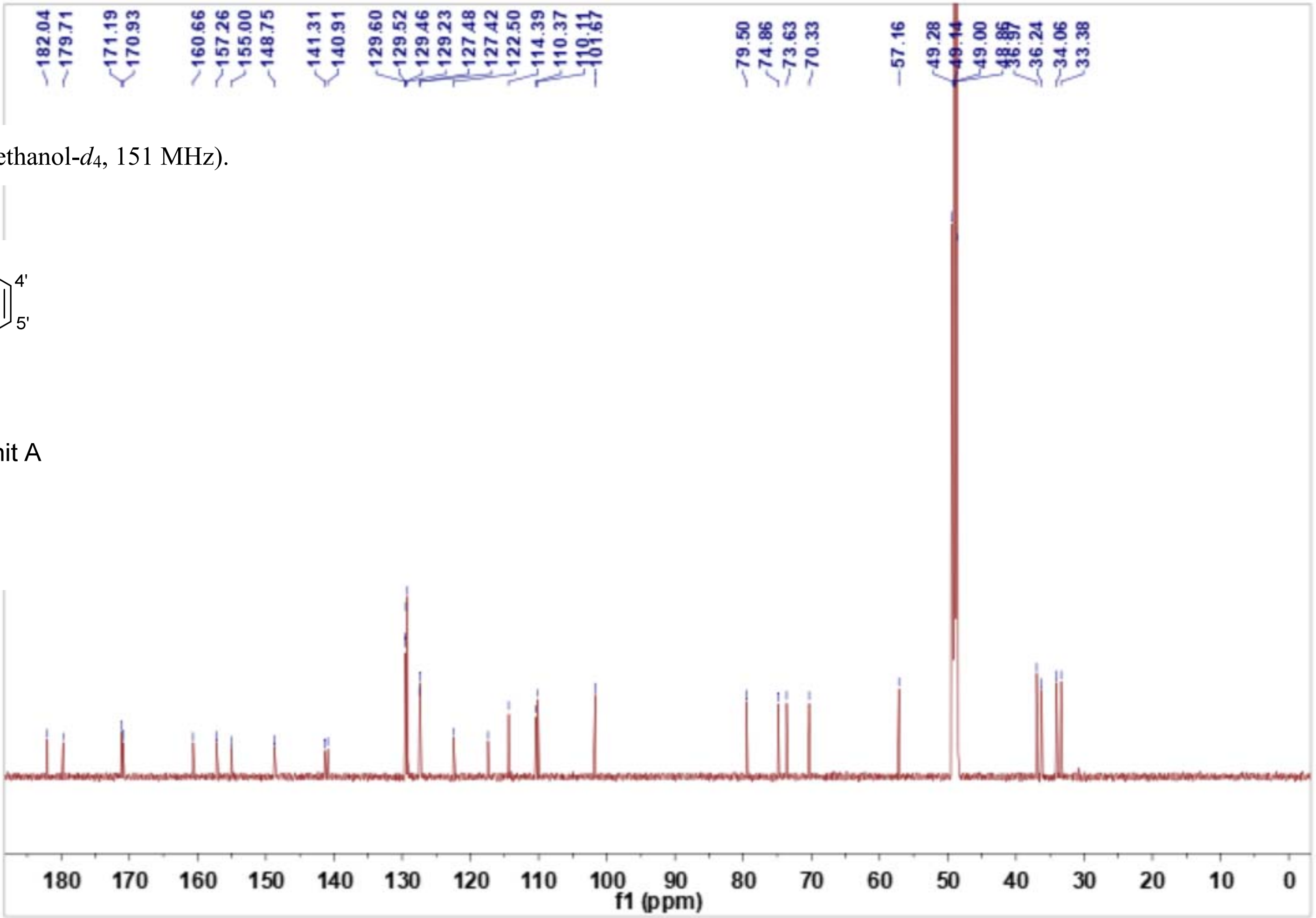

Fig. S37. HSQC spectrum of **5**.

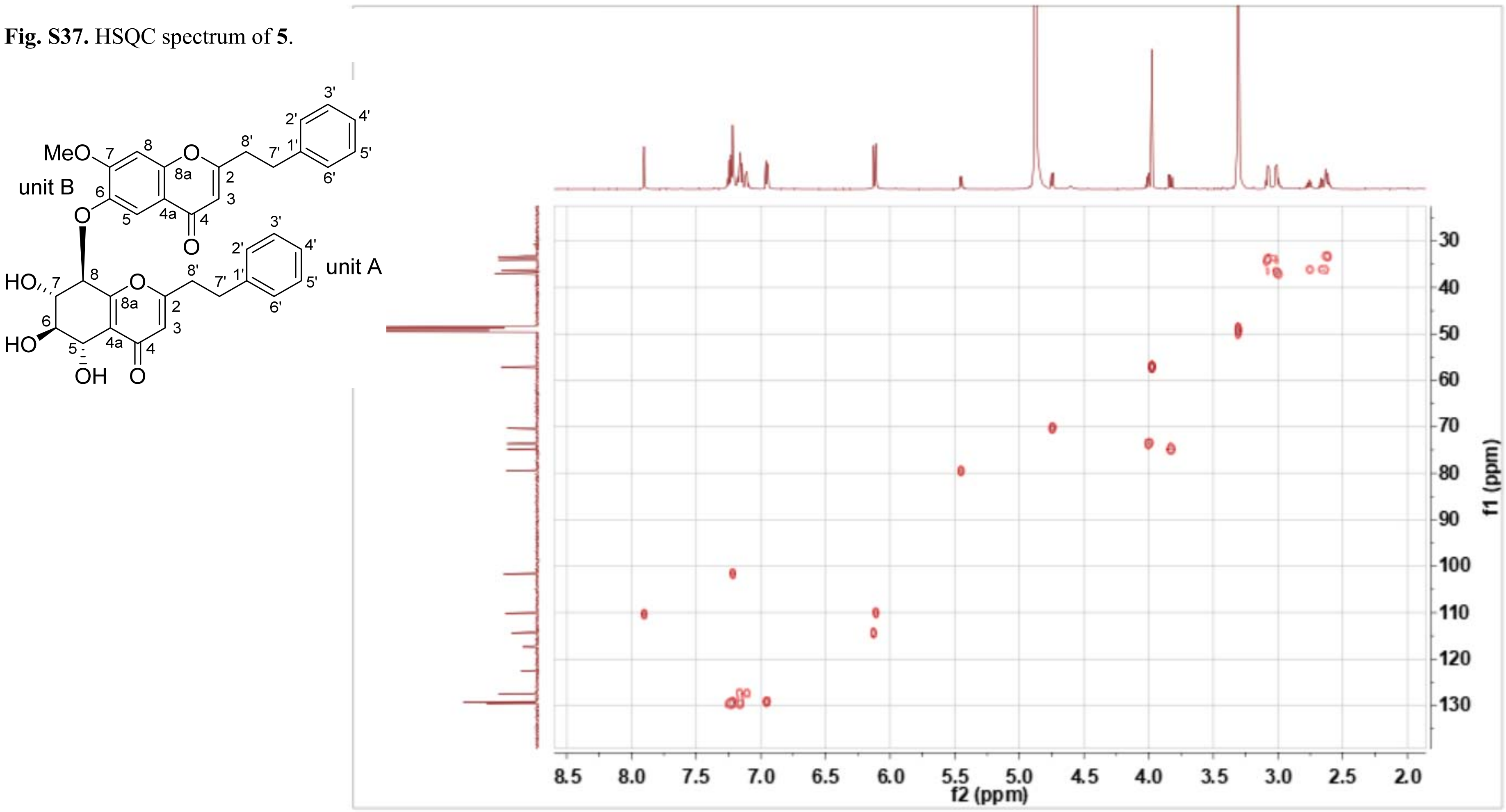

Fig. S38. ^{1}H–^{1}H COSY spectrum of **5**.

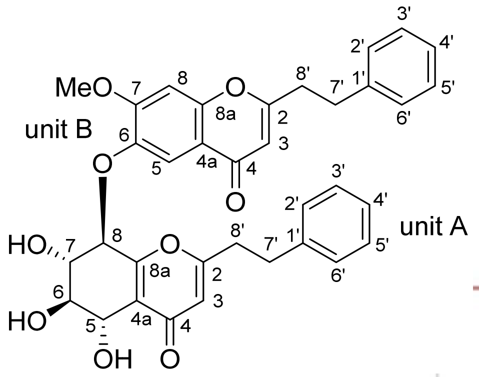

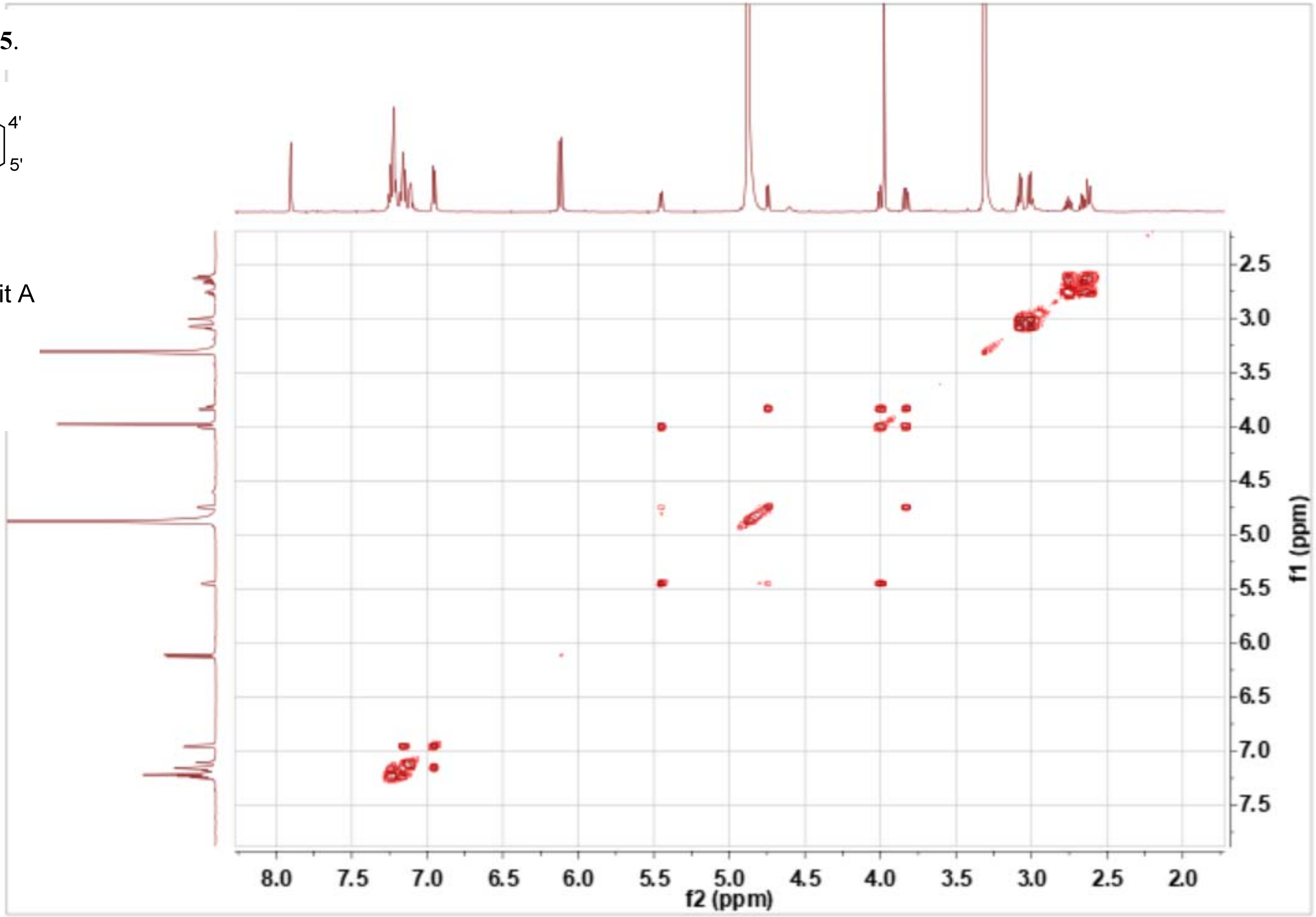

Fig. S39. HMBC spectrum of **5**.

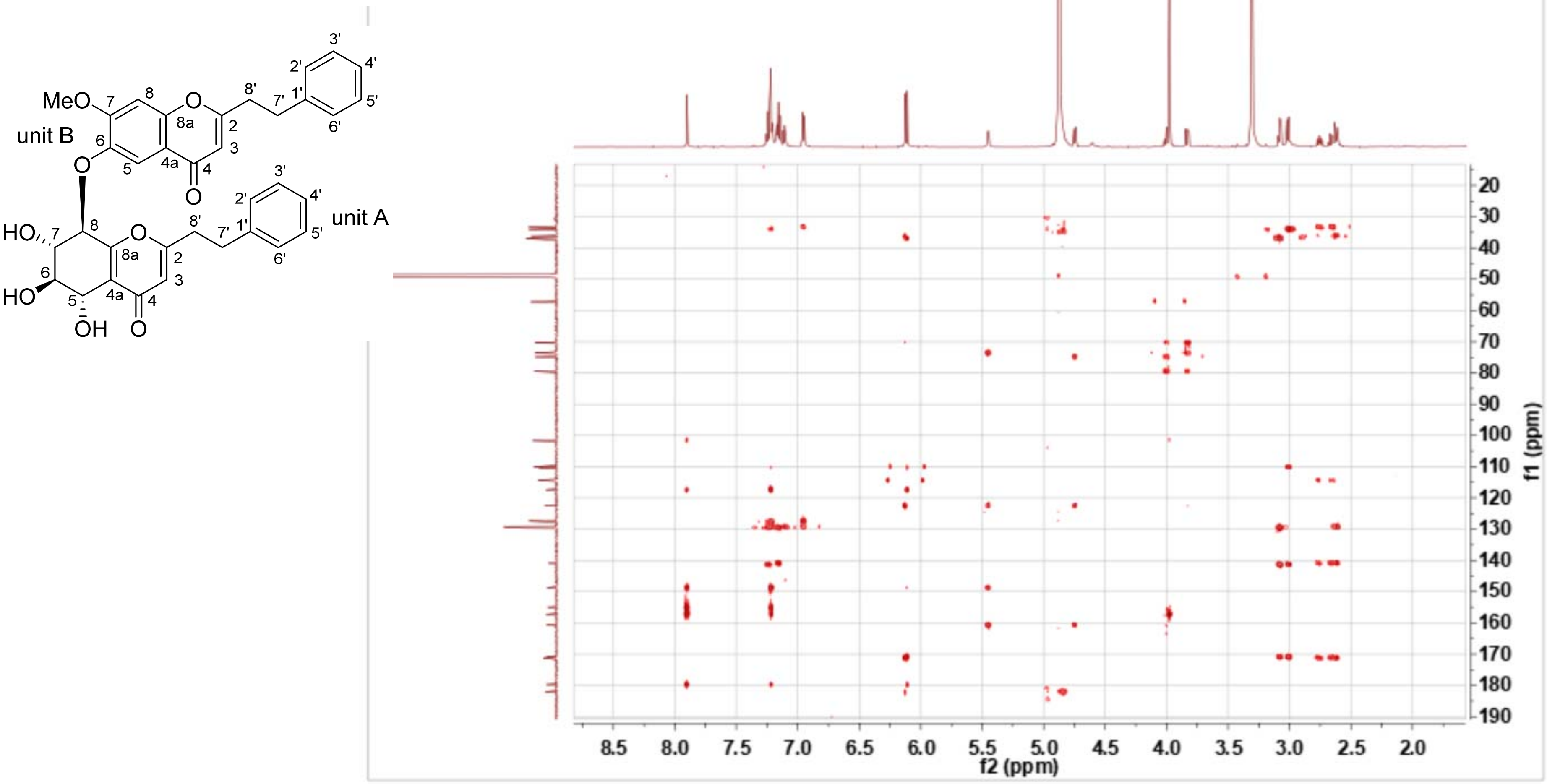

Fig. S40. ROESY spectrum of **5**.

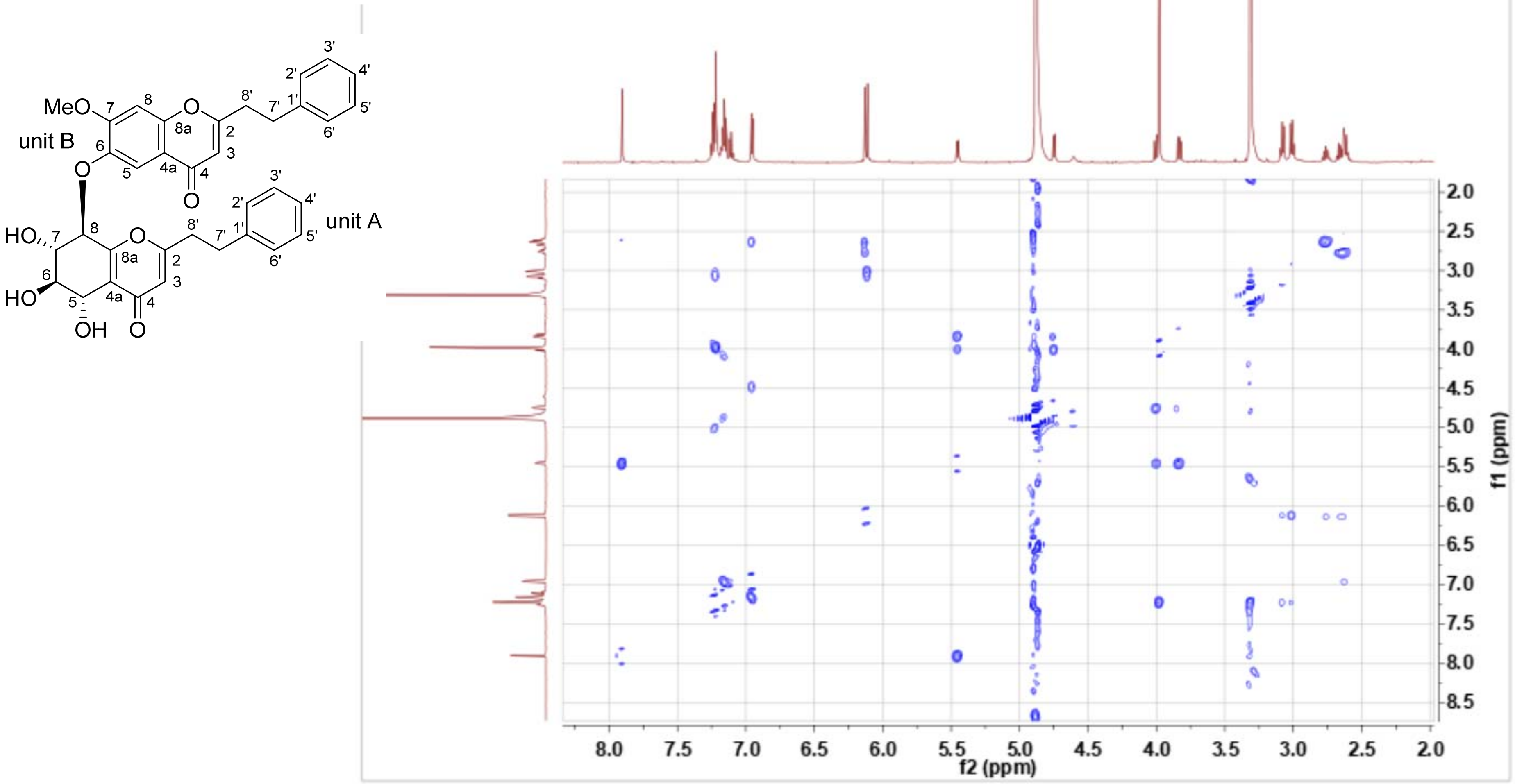

Fig. S41. HRESIMS spectrum of **5**.

Qualitative Analysis Report

**Data Filename**	20200408ESIA1.d	**Sample Name**	pes48
**Sample Type**	Sample	**Position**	
**Instrument Name**	Agilent G6230 TOF MS	**User Name**	KIB
**Acq Method**	ESI.m	**Acquired Time**	4/3/2020 10:36:18 AM
**IRM Calibration Status**	Success [redacted]	**DA Method**	ESI.m
**Comment**			

Sample Group **Info.**

Acquisition SW Version 6200 series TOF/6500 series Q-TOF B.05.01 (B5125.2)

User Spectra

Fragmentor Voltage	Collision Energy	Ionization Mode
200	0	ESI

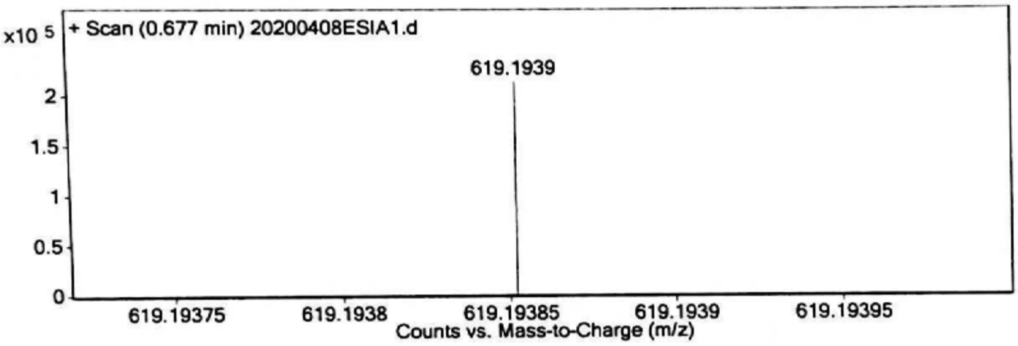

Peak List

m/z	z	Abund	Formula	Ion
121.0509	1	253900.06		
164.0661	1	34503.57		
173.0666	1	55601.77		
301.1408	1	113363.21		
619.1939	1	214795.95	C35 H32 Na O9	M+
620.1971	1	82779.2	C35 H32 Na O9	M+
922.0098	1	299801.5		
923.0122	1	54811.11		
1215.3971	1	86247.47		
1216.3999	1	70781.76		

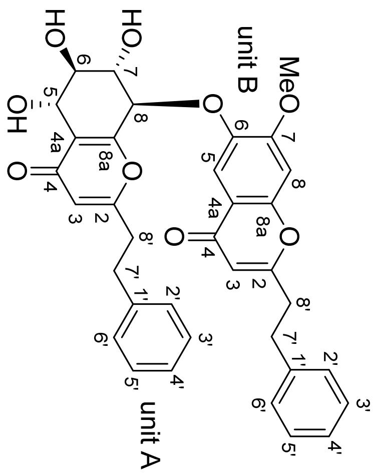

Formula Calculator Element Limits

Element	Min	Max
C	0	200
H	0	400
O	5	13
Na	1	1

Formula Calculator Results

Formula	CalculatedMass	Mz	Diff.(mDa)	Diff. (ppm)	DBE
C35 H32 Na O9	619.1944	619.1939	0.5	0.8	19.5

--- End Of Report ---

Fig. S42. ECD spectrum of **5**.

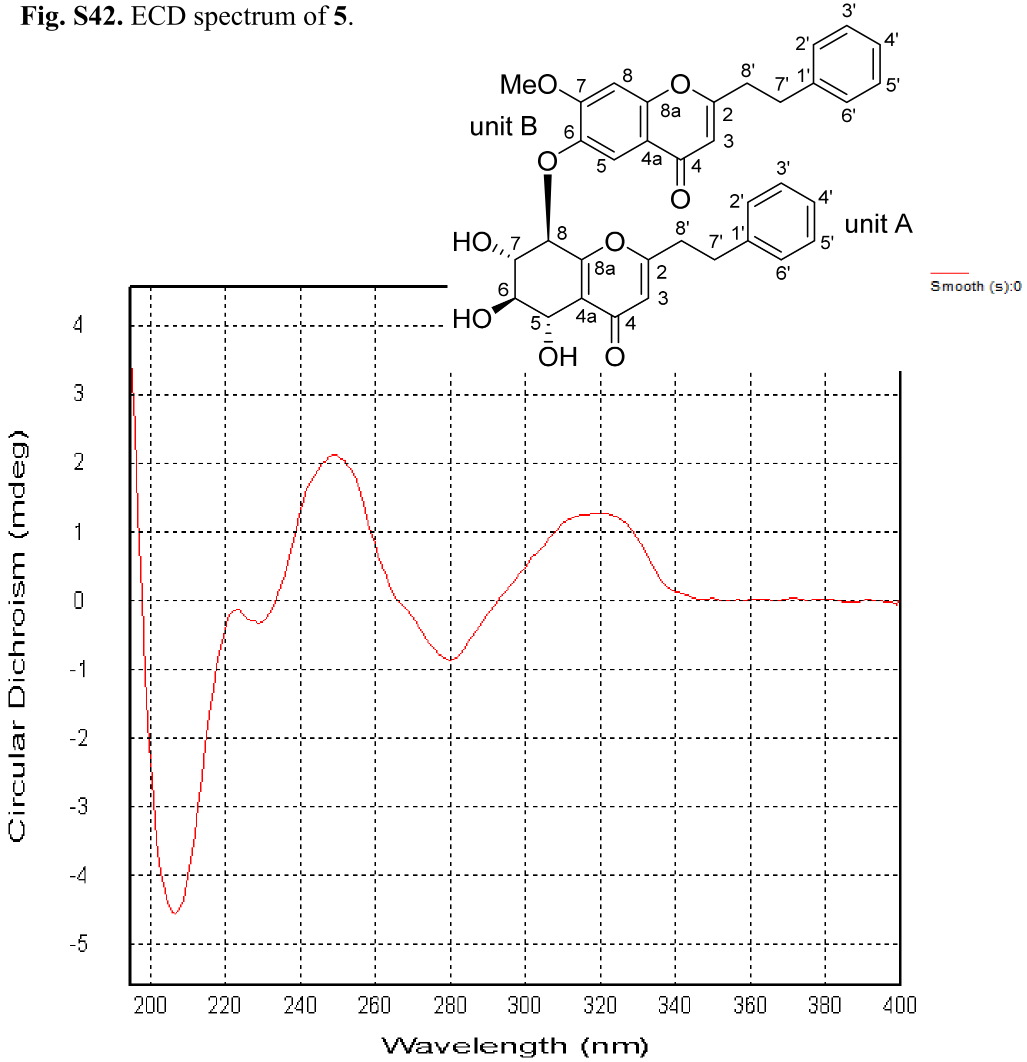

File: PES48(195-400nm)19112823.dsx
ProBinaryX
Attributes :
- Time Stamp :Thu Nov 28 19:11:46 2019

- File ID : {75755780-41D6-4282-B678-93759E12B8DA}
- Is CFR Compliant : false
- Original data has not been modified.

Remarks:
- User: CD
- Date: 2019/11/28
- Instrument: 0547
- DetectorType: LAAPD
- DichOS Calibration Correction Curve: 0547/2
- HV (CDDC channel): 0 v
- Time per point: 1 s
- Description: Sample 1
- Concentration: 0.0408mg/mL MeOH
- Pathlength: 1 mm
- Temperature: 20℃

Settings:
- Time-per-point: 1s (25us x 40000)
- SE
- Wavelength: 195nm - 400nm
- Step Size: 1nm
- Bandwidth: 1nm

Fig. S43. ^{1}H NMR spectrum of **6** (methanol-d_4, 500 MHz).

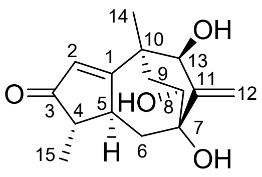

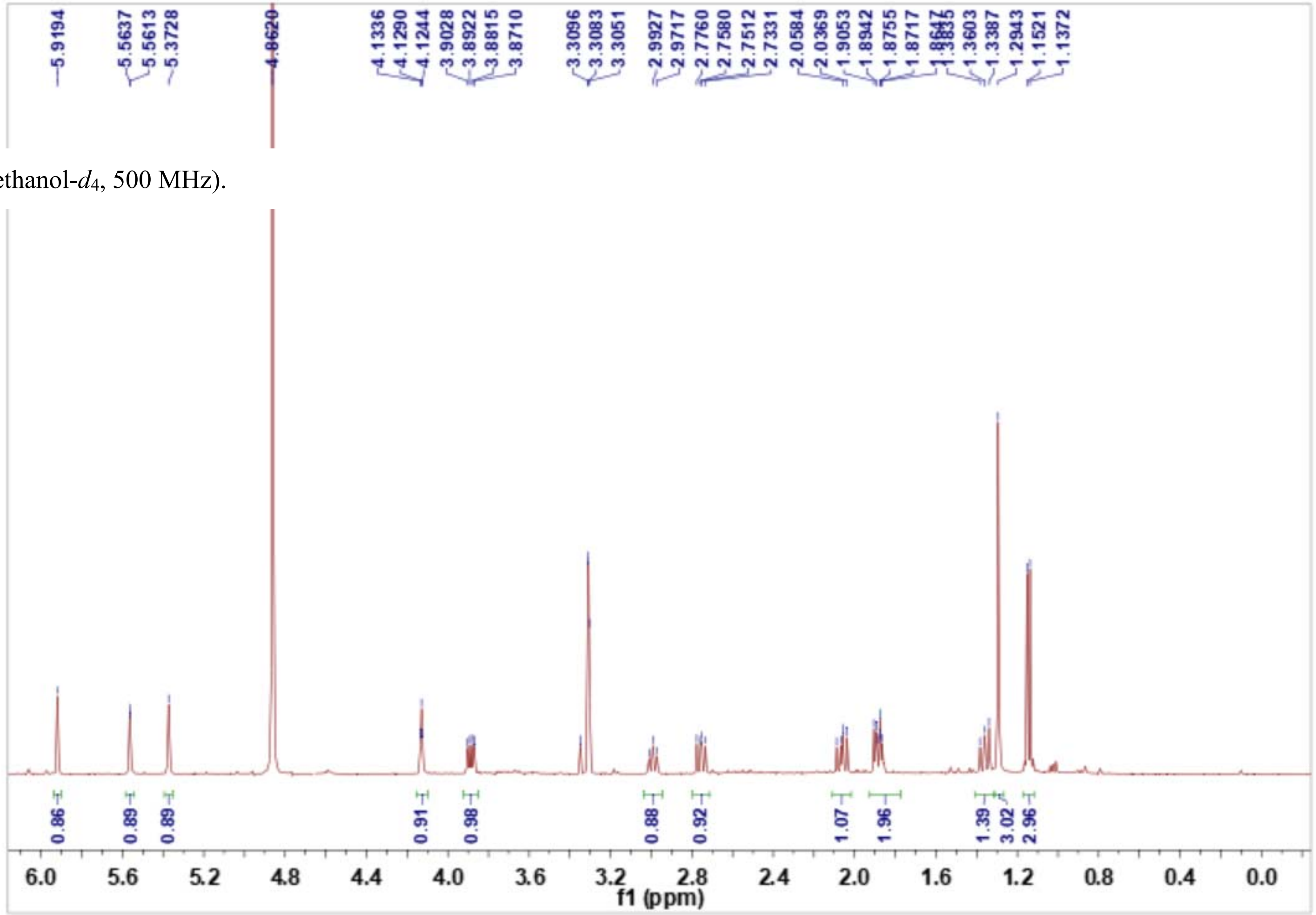

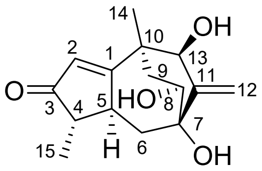

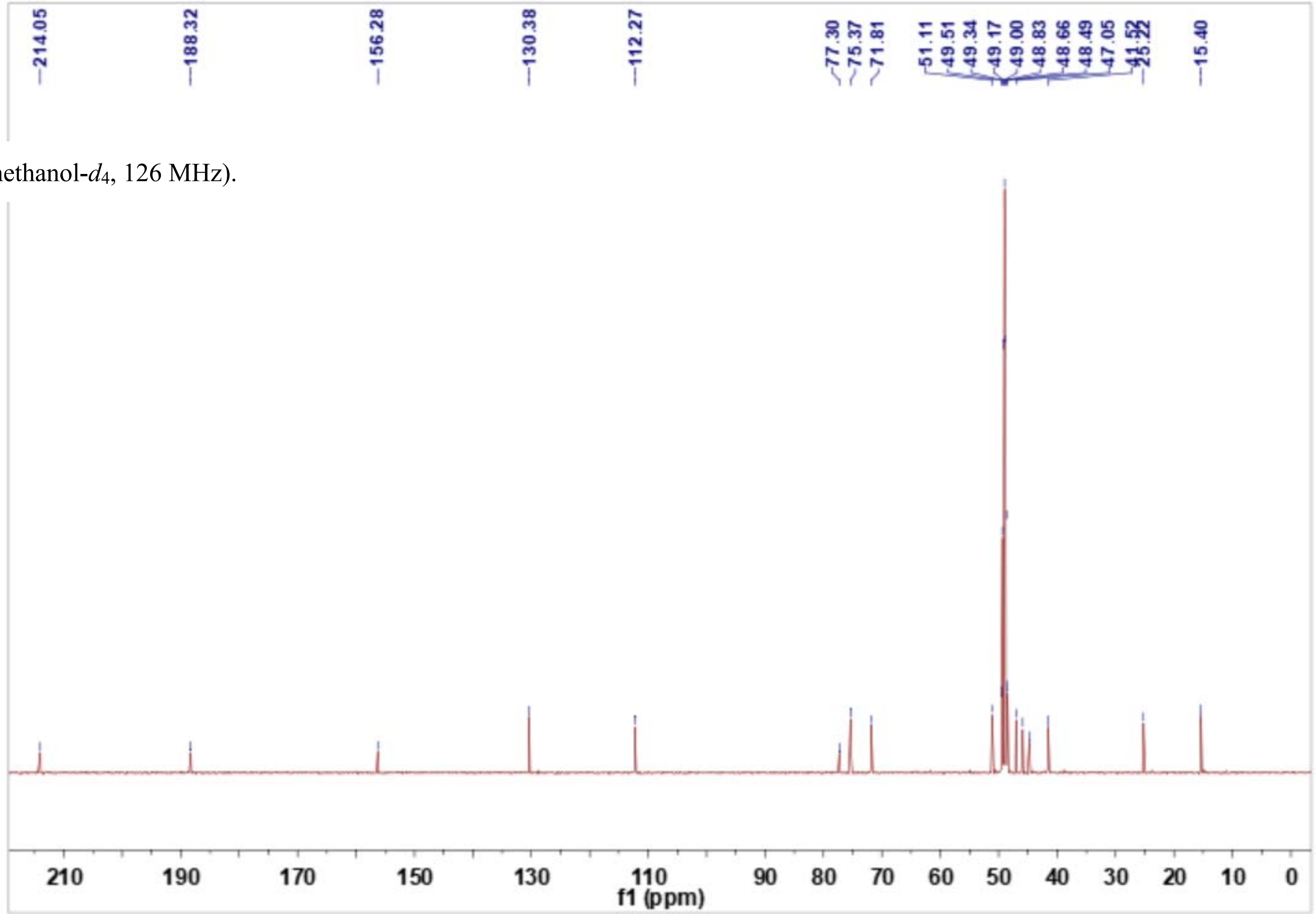

Fig. S44. ^{13}C NMR spectrum of **6** (methanol-d_4, 126 MHz).

Fig. S45. HSQC spectrum of **6**.

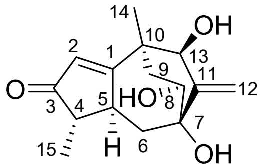

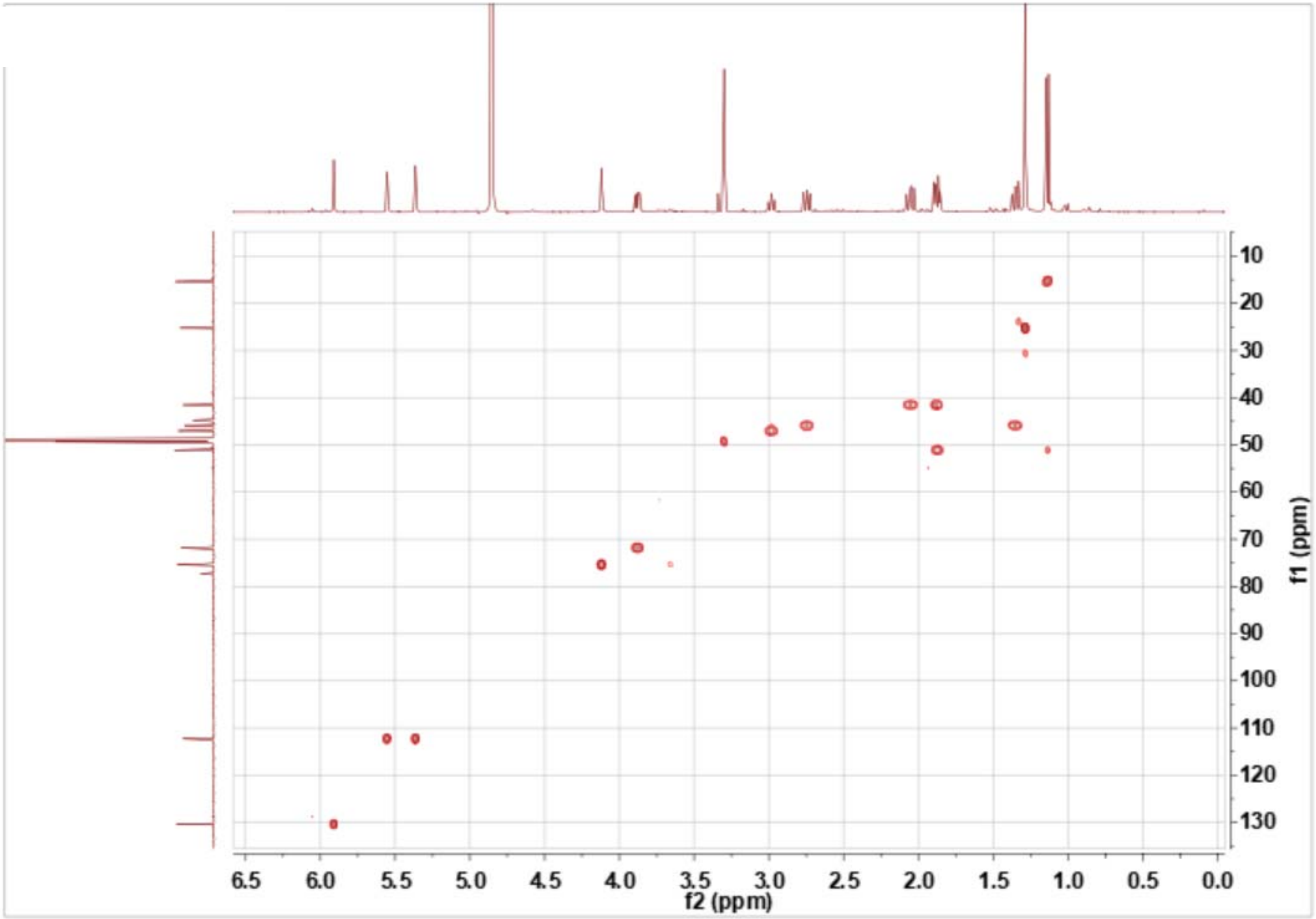

Fig. S46. ^1H–^1H COSY spectrum of **6**.

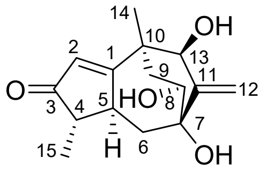

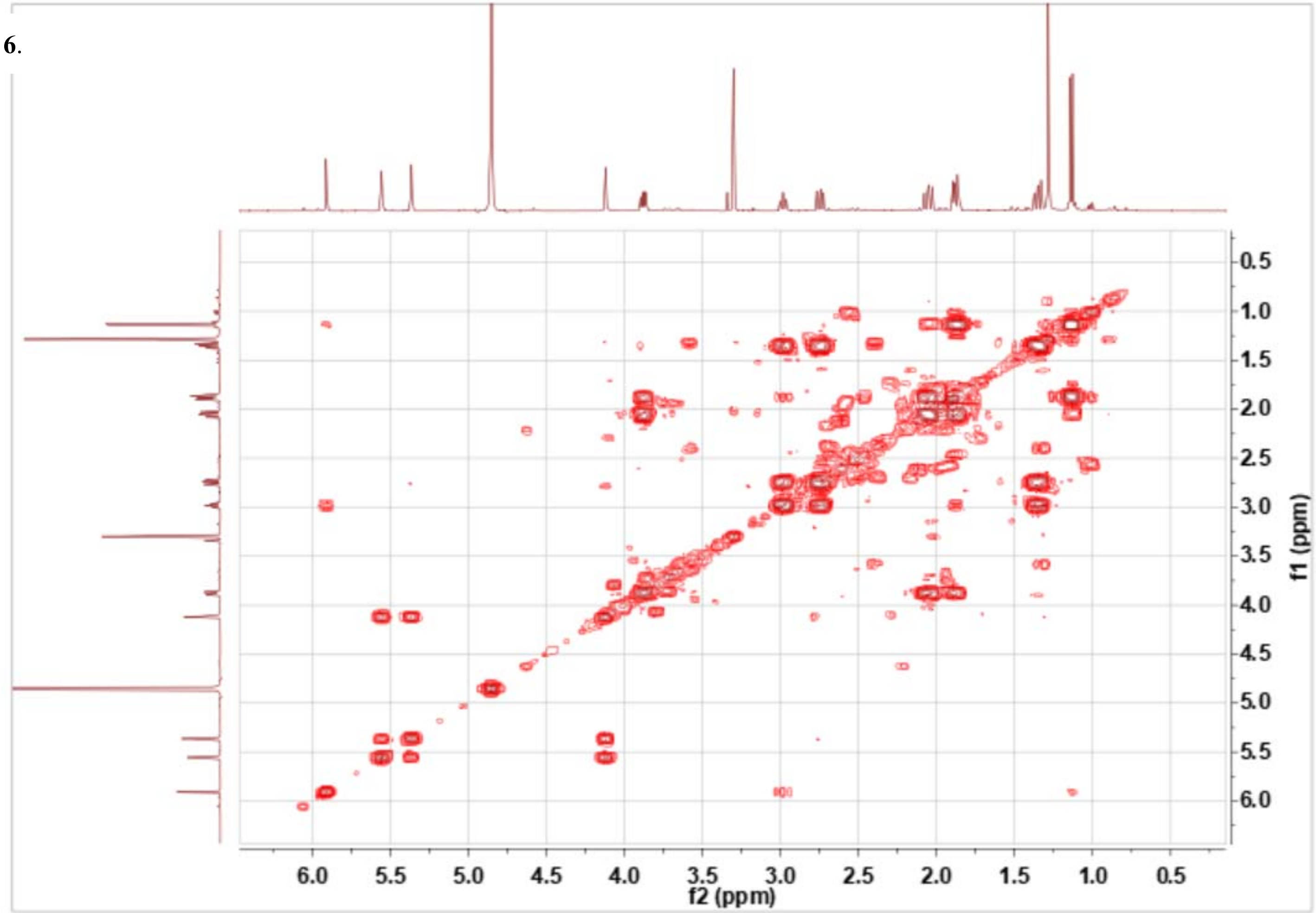

Fig. S47. HMBC spectrum of **6**.

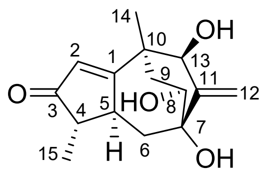

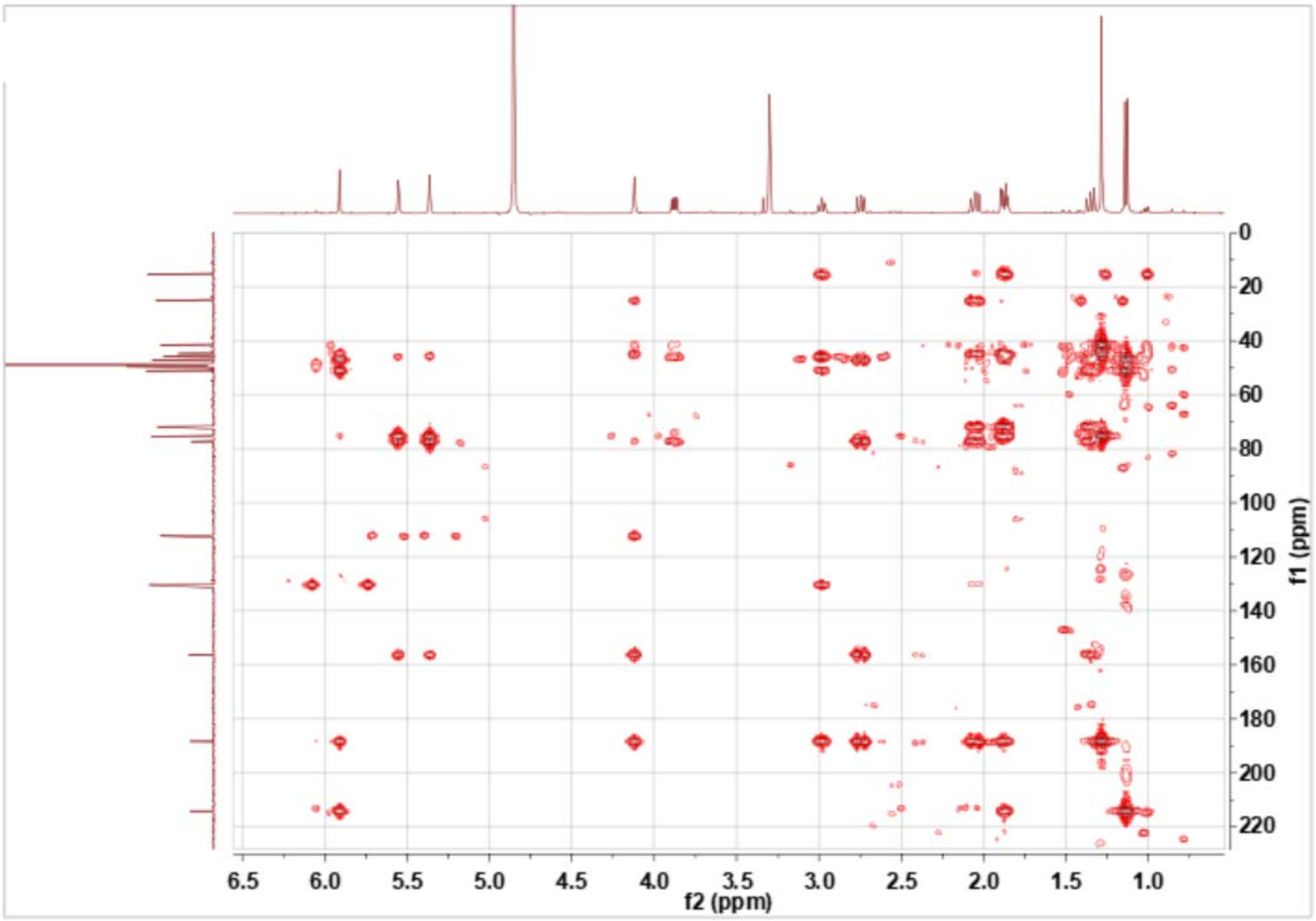

Fig. S48. ROESY spectrum of **6**.

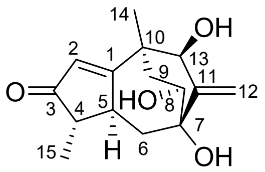

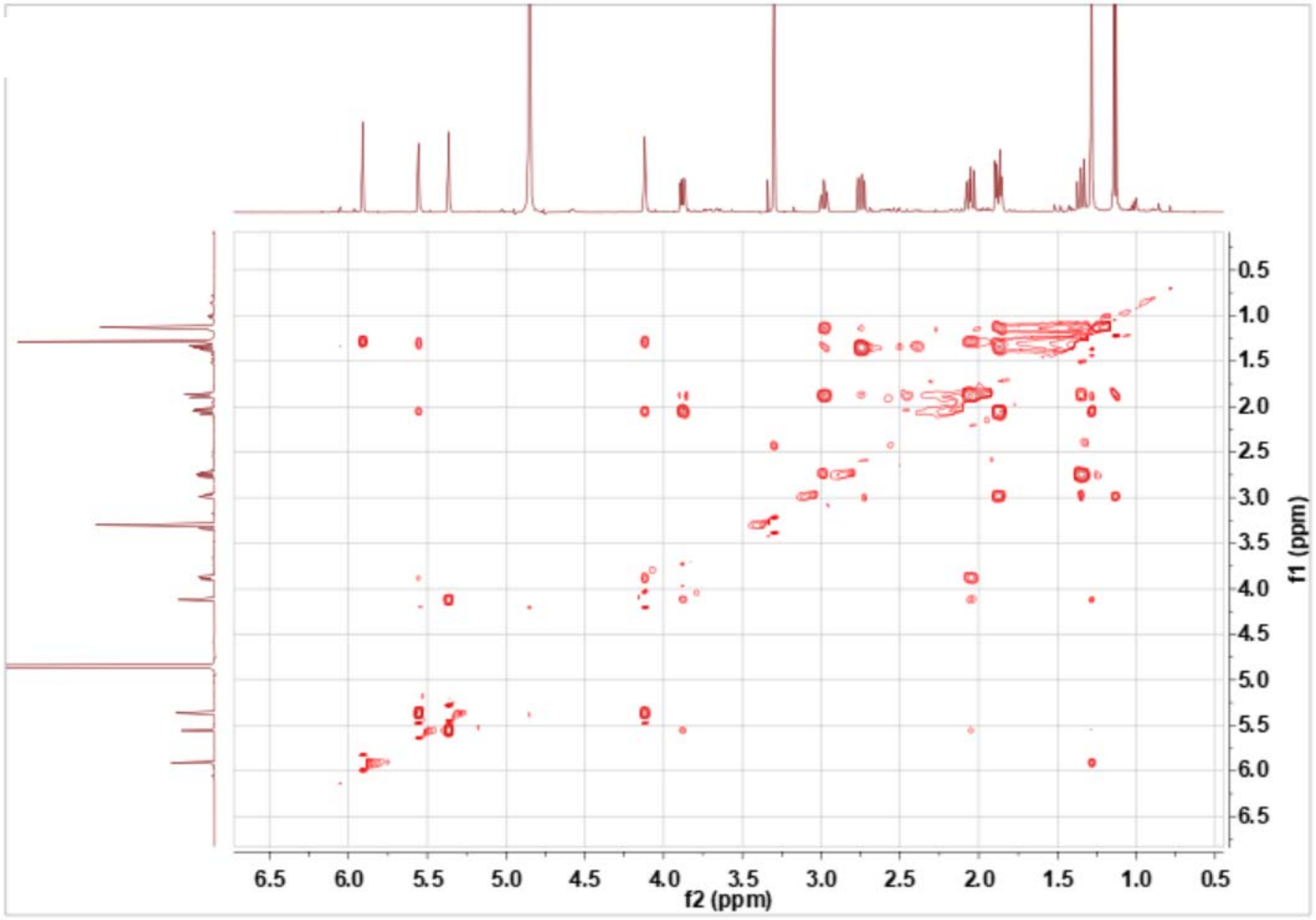

Fig. S49. HRESIMS spectrum of **6**.

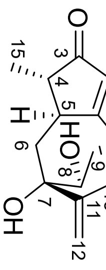

Qualitative Analysis Report

**Data Filename**	190614ESIA4.d	**Sample Name**	pes5a
**Sample Type**	Sample	**Position**	
**Instrument Name**	Agilent G6230 TOF MS	**User Name**	KIB
**Acq Method**	ESI.m	**Acquired Time**	6/13/2019 2:51:15 PM
**IRM Calibration Status**	[redacted]	**DA Method**	ESI.m
**Comment**			

**Sample Group**		**Info.**
**Acquisition SW**	6200 series TOF/6500 series	
**Version**	Q-TOF B.05.01 (B5125.2)	

User Spectra

Fragmentor Voltage	Collision Energy	Ionization Mode
200	0	ESI

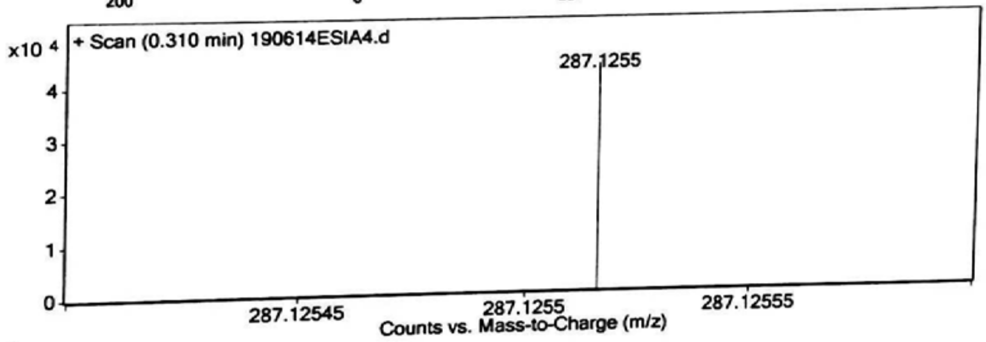

Peak List

m/z	z	Abund	Formula	Ion
121.0509		28356.78		
287.1255	1	43644.62	C15 H20 Na O4	M+
305.1415	1	58157.72		
321.1292	1	38966.68		
323.1429	1	31126.91		
328.1519	1	33387.46		
349.1832	1	42084.13		
393.2096	1	51687.73		
437.2341	1	54478.42		
481.2623	1	40991.34		

Formula Calculator Element Limits

Element	Min	Max
C	0	200
H	0	400
O	0	10
Na	1	1

Formula Calculator Results

Formula	CalculatedMass	Mz	Diff.(mDa)	Diff. (ppm)	DBE
C15 H20 Na O4	287.1259	287.1255	0.4	1.5	5.5

--- End Of Report ---

Fig. S50. ECD spectrum of **6**.

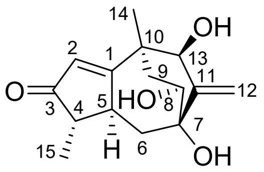

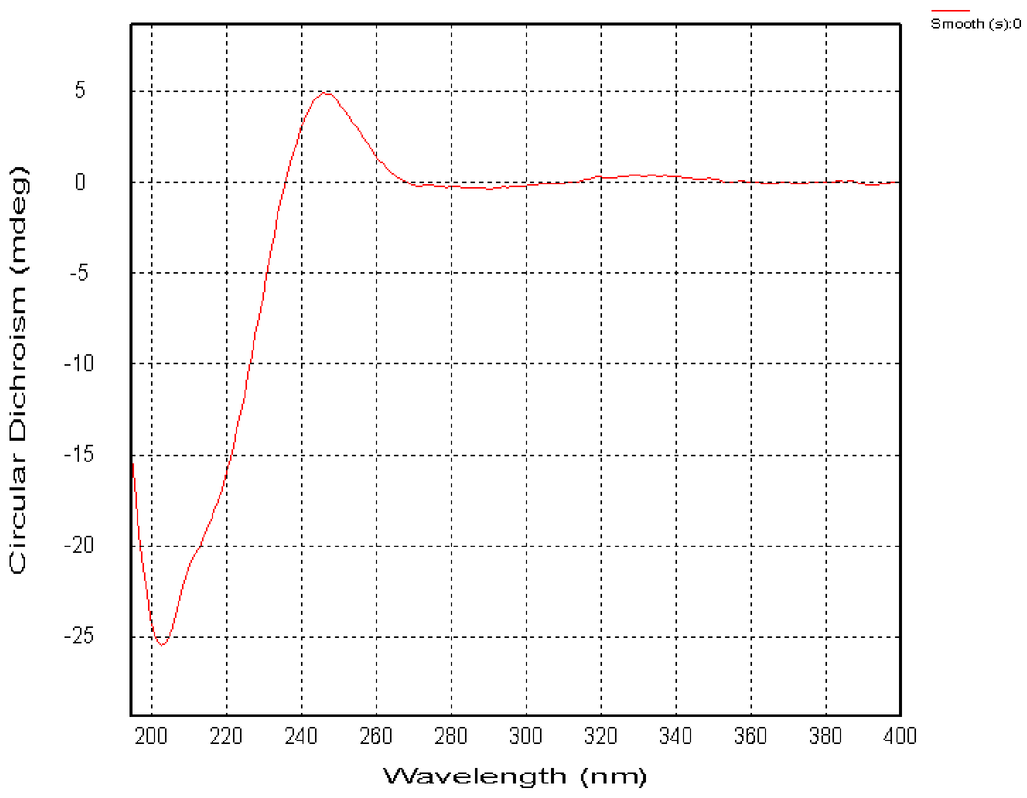

File: CD PES5A-1mm(195-400)19061506.dsx
ProBinaryX
Attributes :
- Time Stamp :Sat Jun 15 14:45:01 2019

- File ID : {C4DEA3EB-A78C-44f1-8276-4B544376A31C}
- Is CFR Compliant : false
- Original data has not been modified.

Remarks:
- User: APLService
- Date: 2019/06/15
- Instrument: 0218
- DetectorType: PMT
- DichOS Calibration Correction Curve: 0218/1
- HV (CDDC channel): 0 v
- Time per point: 1 s
- Description: Sample 1
- Concentration: 0.1500mg/mL MeOH
- Pathlength: 1 mm
- Temperature: 20℃

Settings:
- Time-per-point: 1s (25us x 40000)
- SE
- Wavelength: 195nm - 400nm
- Step Size: 1nm
- Bandwidth: 1nm

Fig. S51. ^{1}H NMR spectrum of **7** (methanol-d_4, 800 MHz).

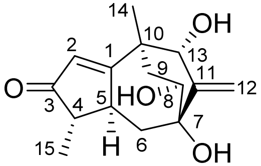

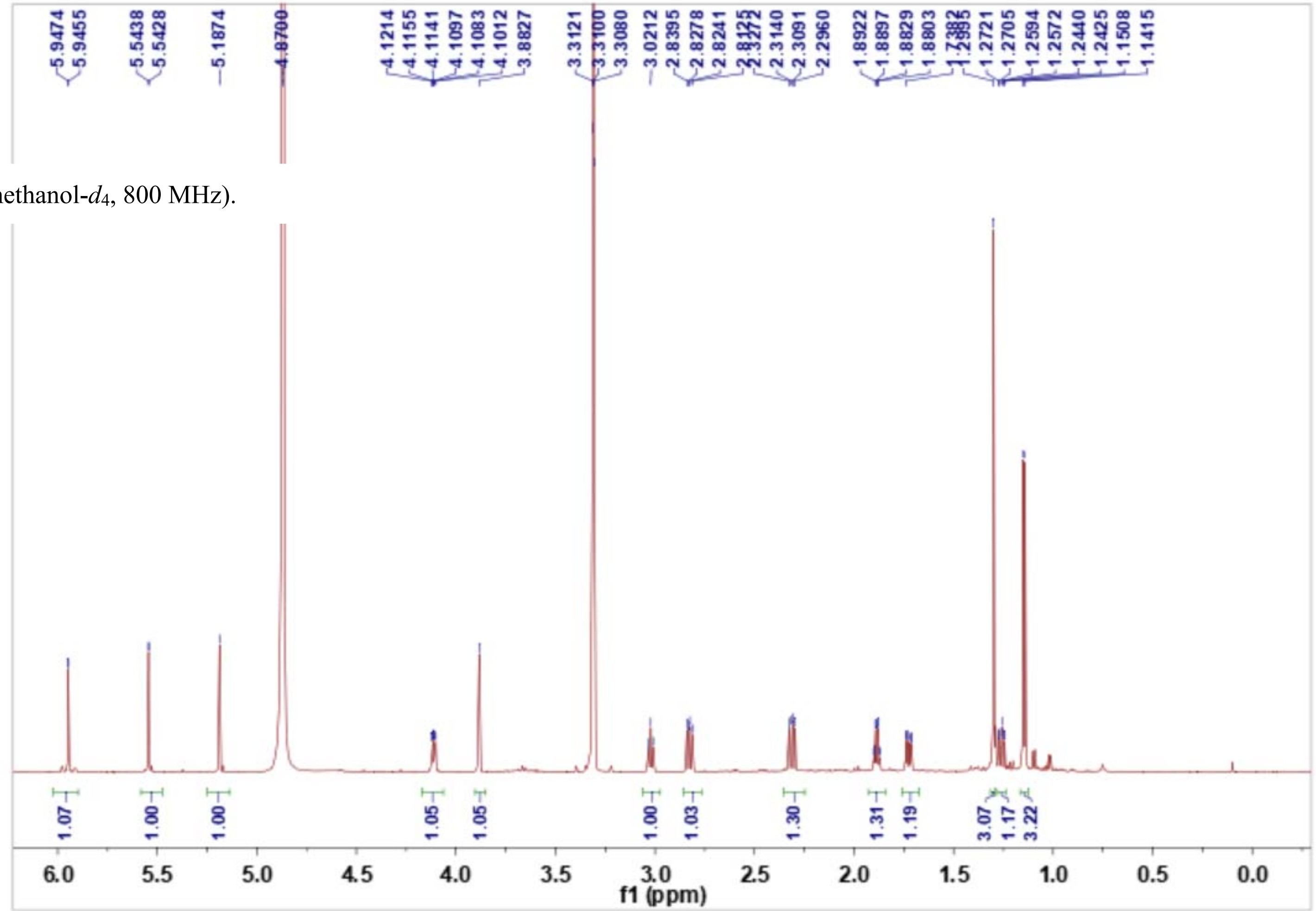

Fig. S52. ^{13}C NMR spectrum of **7** (methanol-d_4, 201 MHz).

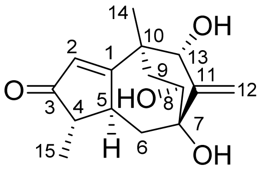

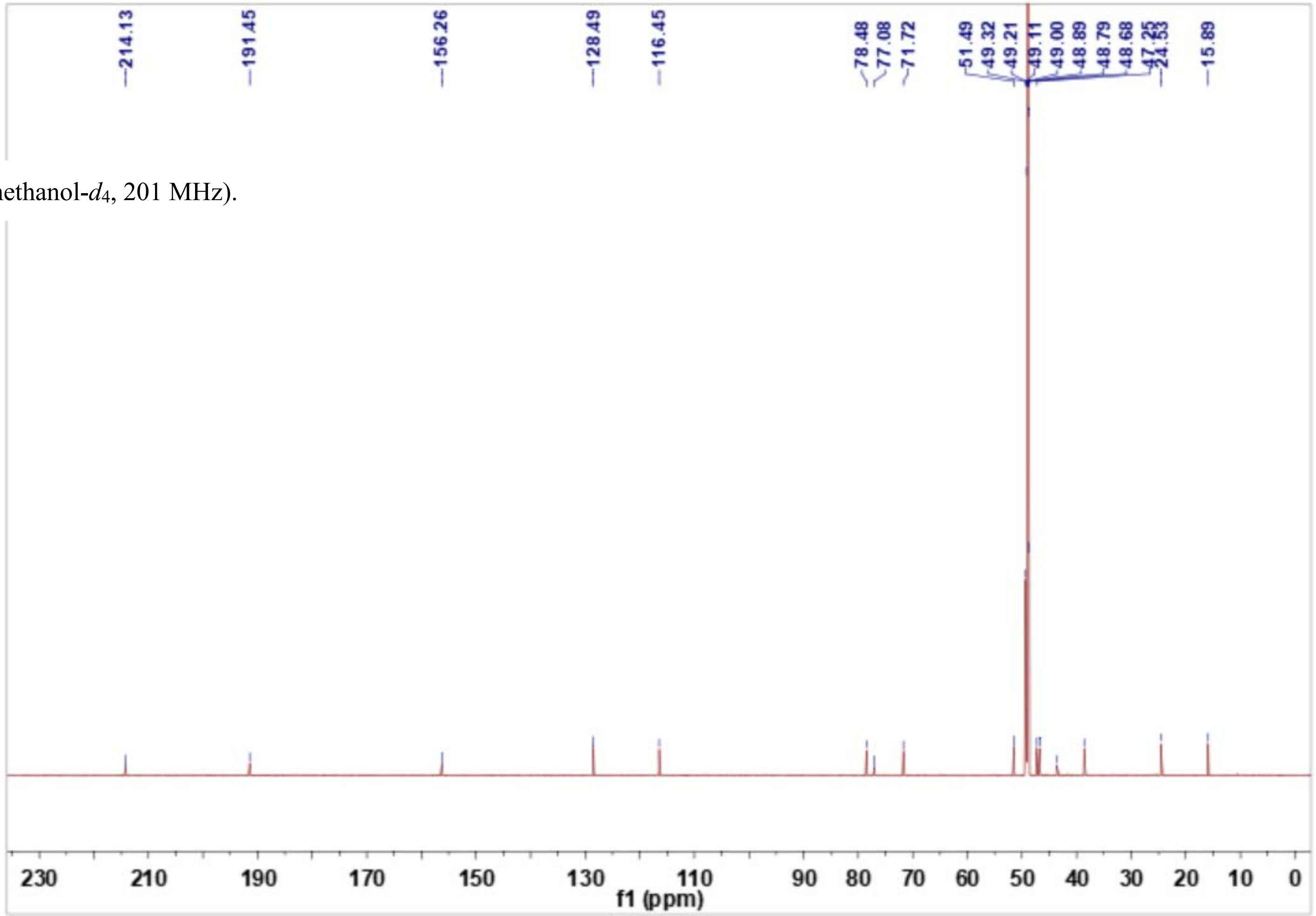

Fig. S53. HSQC spectrum of **7**.

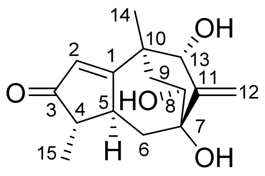

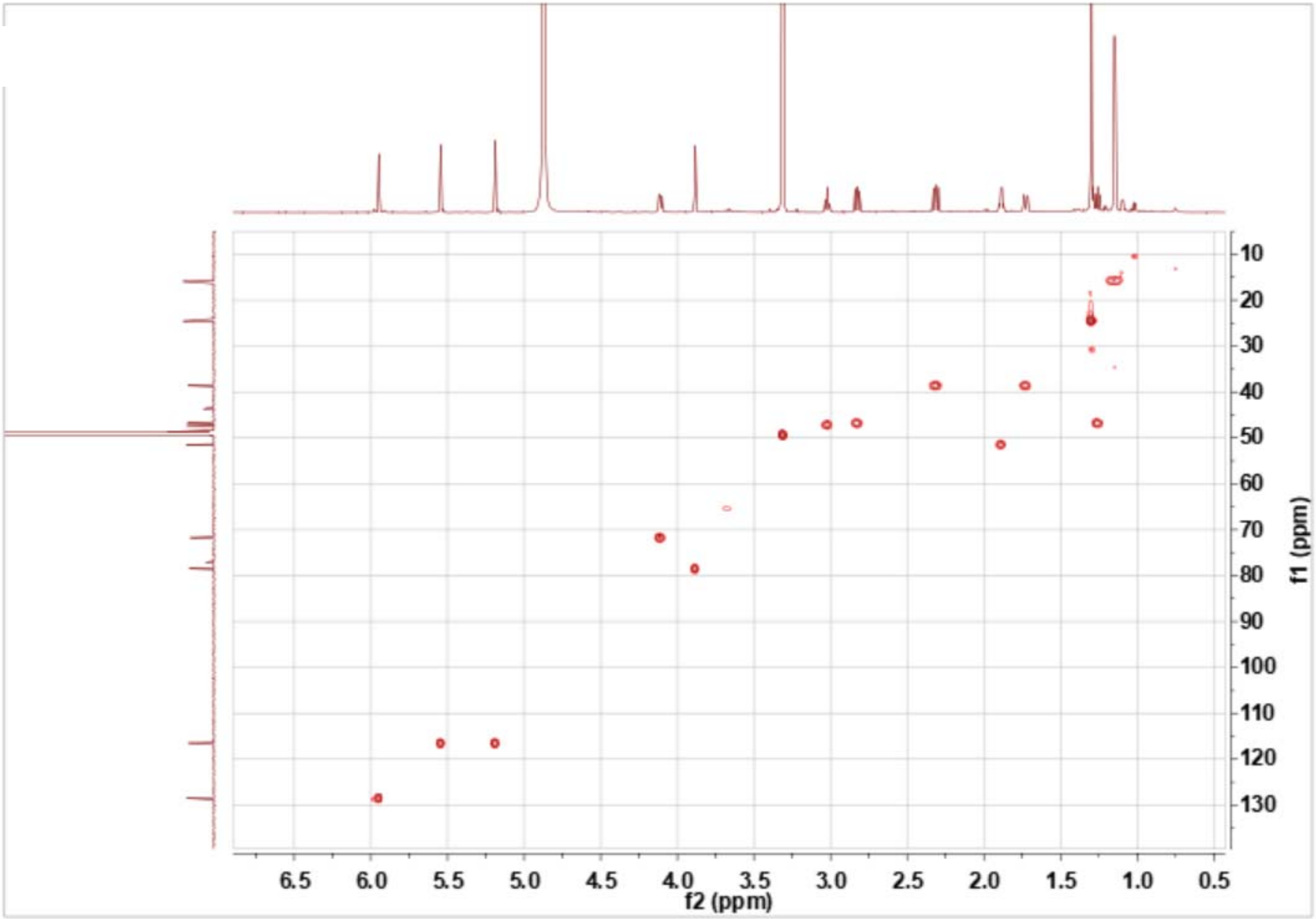

Fig. S55. HMBC spectrum of **7**.

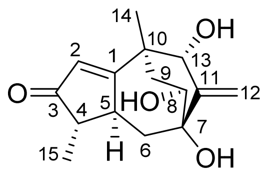

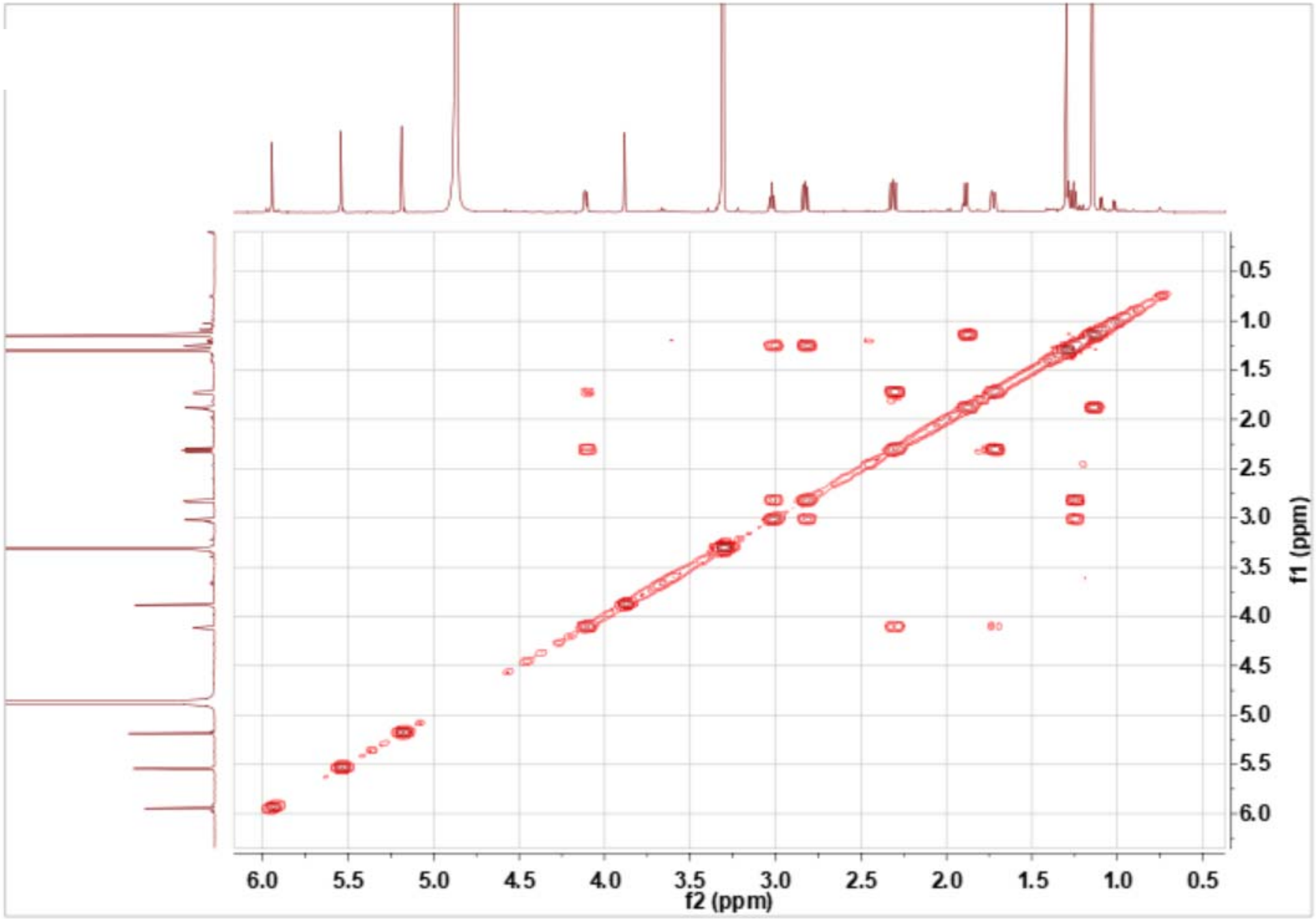

Fig. S55. HMBC spectrum of **7**.

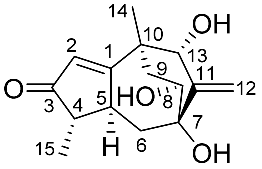

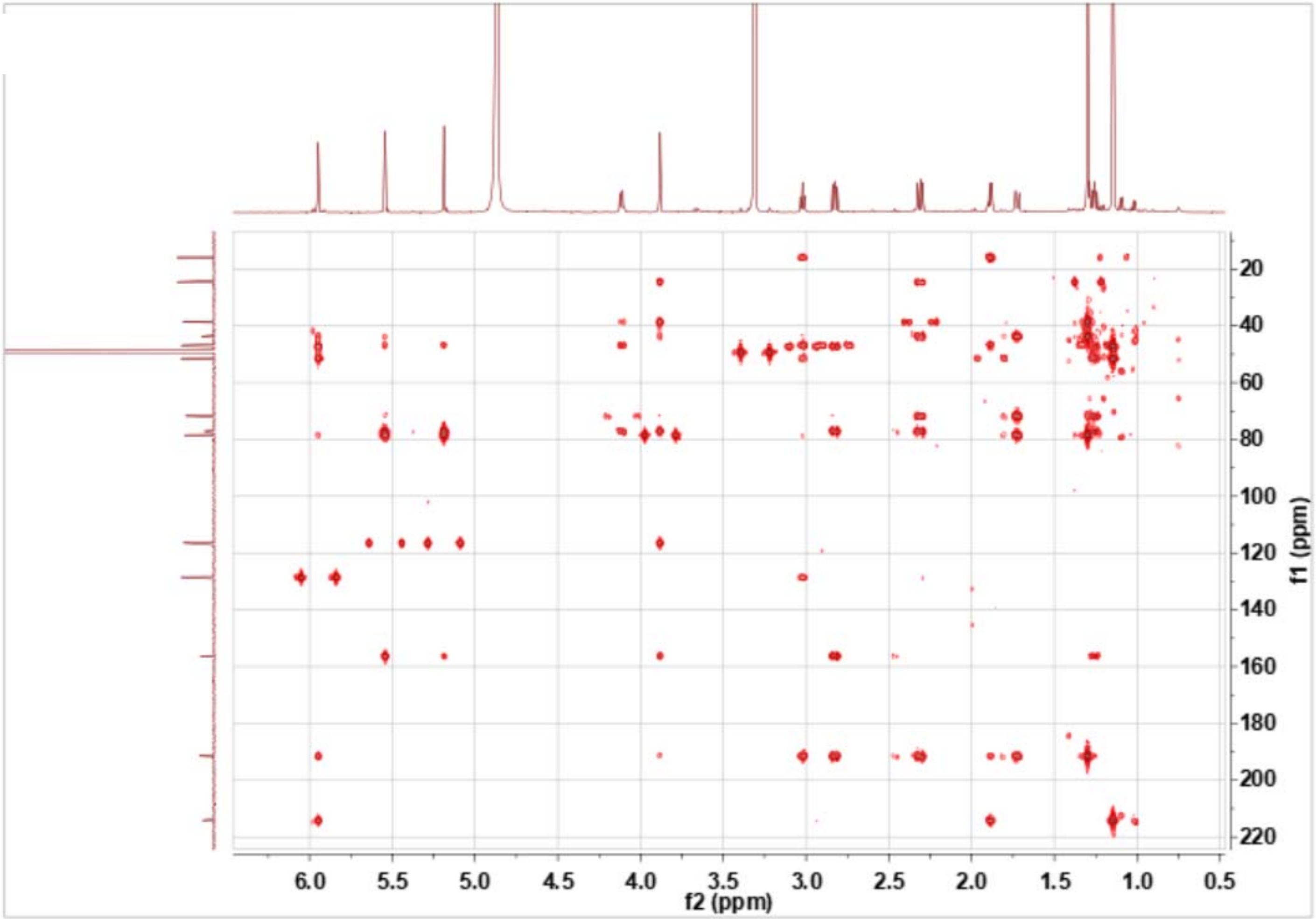

Fig. S56. ROESY spectrum of **7**.

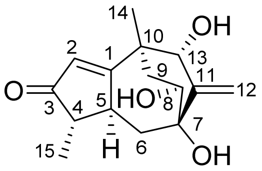

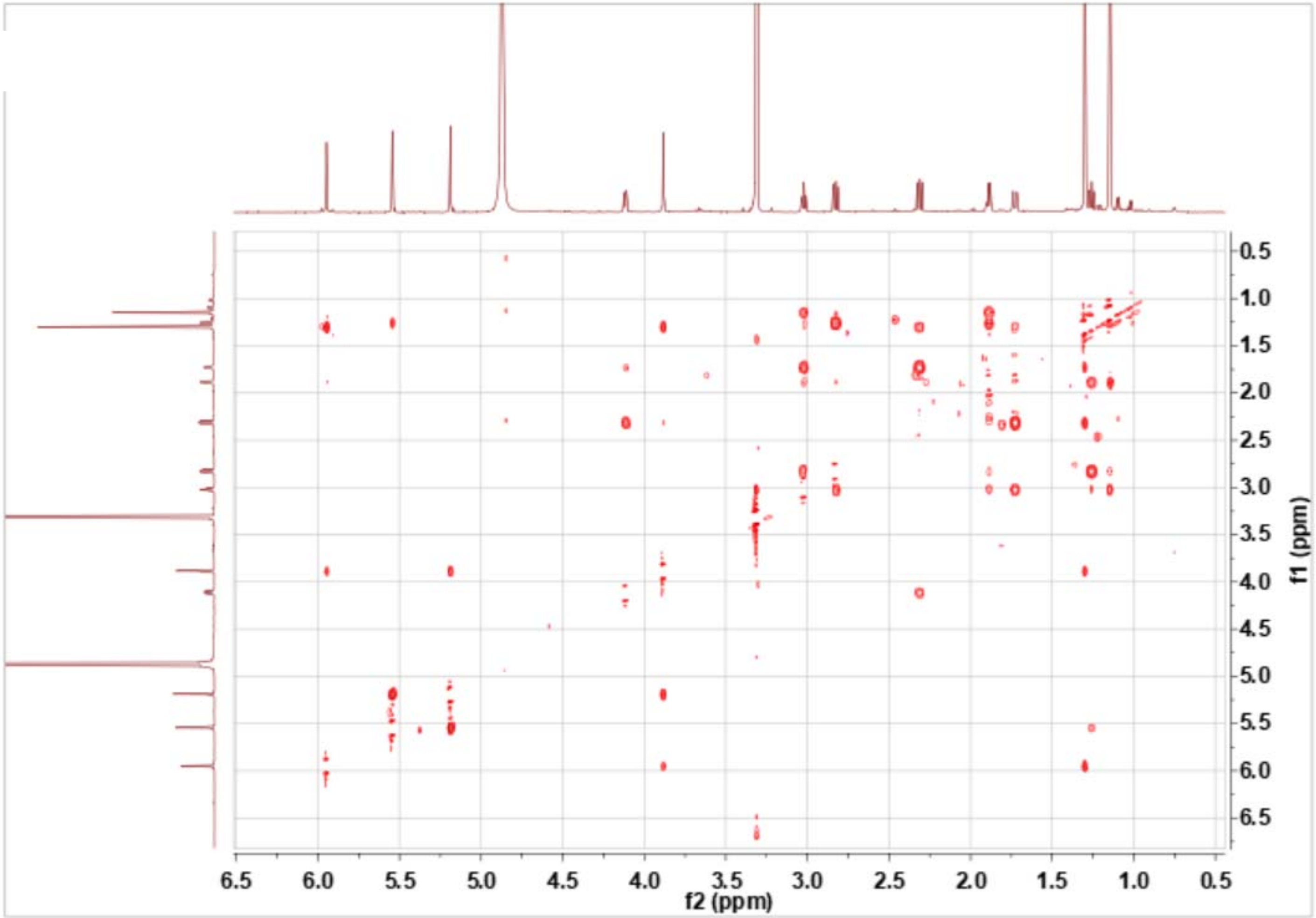

Fig. S57. HRESIMS spectrum of **7**.

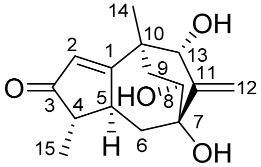

Qualitative Analysis Report

**Data Filename**	190614ESIA3.d	**Sample Name**	pes14
**Sample Type**	Sample	**Position**	
**Instrument Name**	Agilent G6230 TOF MS	**User Name**	KIB
**Acq Method**	ESI.m	**Acquired Time**	6/13/2019 2:47:41 PM
**IRM Calibration Status**	Success	**DA Method**	ESI.m
**Comment**			

Sample Group **Info.**

Acquisition SW Version 6200 series TOF/6500 series Q-TOF B.05.01 (B5125.2)

User Spectra

Fragmentor Voltage	Collision Energy	Ionization Mode
200	0	ESI

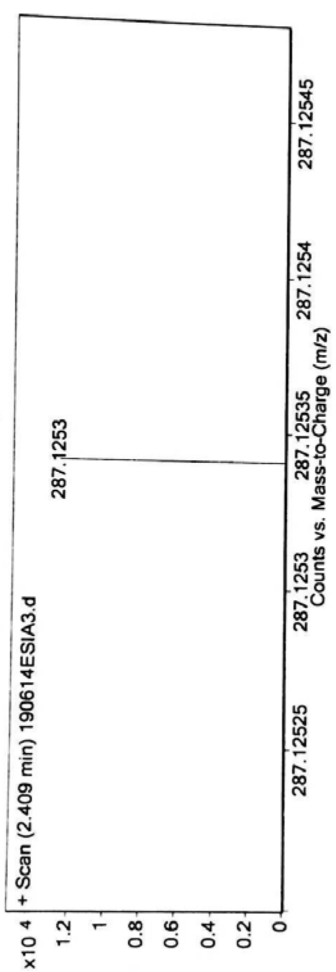

Peak List

m/z	z	Abund
102.1283	1	100674.15
121.0509	1	120277.59
186.2212	1	31190.89
303.1205		71541.98
304.1252		35969.71
305.1362	1	62793.72
307.1513	1	36667.13
323.1463	1	51547.02
437.1982	1	25793
922.0098	1	47798.52

Formula Calculator Element Limits

Element	Min	Max
C	0	200
H	0	400
O	0	10
Na	1	1

Formula Calculator Results

Formula	CalculatedMass	Mz	Diff.(mDa)	Diff. (ppm)	DBE
C15 H20 Na O4	287.1259	287.1253	0.6	2.2	5.5

--- End Of Report ---

Fig. S58. ECD spectrum of **7**.

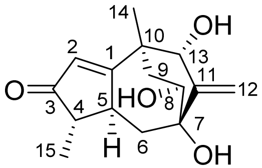

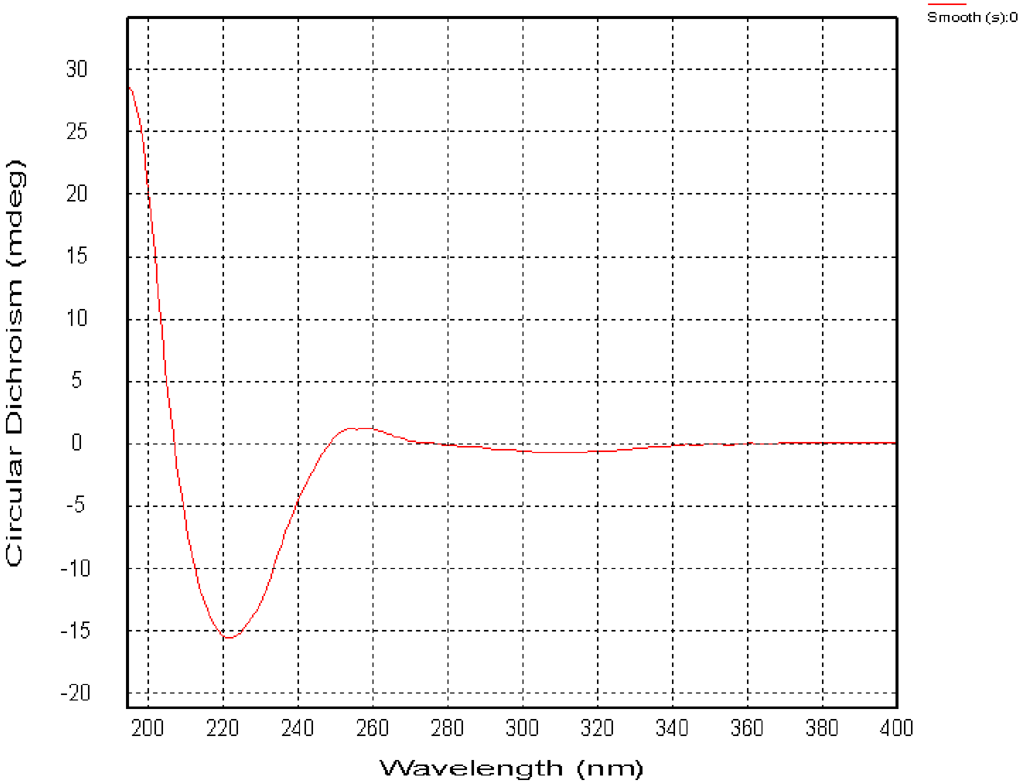

File: CD PES14-1mm(195-400)19061505.dsx

ProBinaryX

Attributes :

- Time Stamp :Sat Jun 15 14:20:04 2019

- File ID : {72D2FBCF-40AE-4de7-8F8E-1DF9D63216C6}
- Is CFR Compliant : false
- Original data has not been modified.

Remarks:

- User: APLService
- Date: 2019/06/15
- Instrument: 0218
- DetectorType: PMT
- DichOS Calibration Correction Curve: 0218/1
- HV (CDDC channel): 0 v
- Time per point: 1 s
- Description: Sample 1
- Concentration: 0.1800mg/mL MeOH
- Pathlength: 1 mm
- Temperature: 20℃

Settings:

- Time-per-point: 1s (25us x 40000)
- SE
- Wavelength: 195nm - 400nm
- Step Size: 1nm
- Bandwidth: 1nm

Fig. S59. ^{1}H NMR spectrum of **8** (methanol-d_4, 800 MHz).

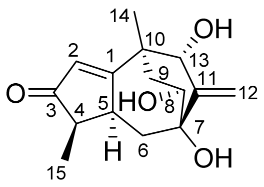

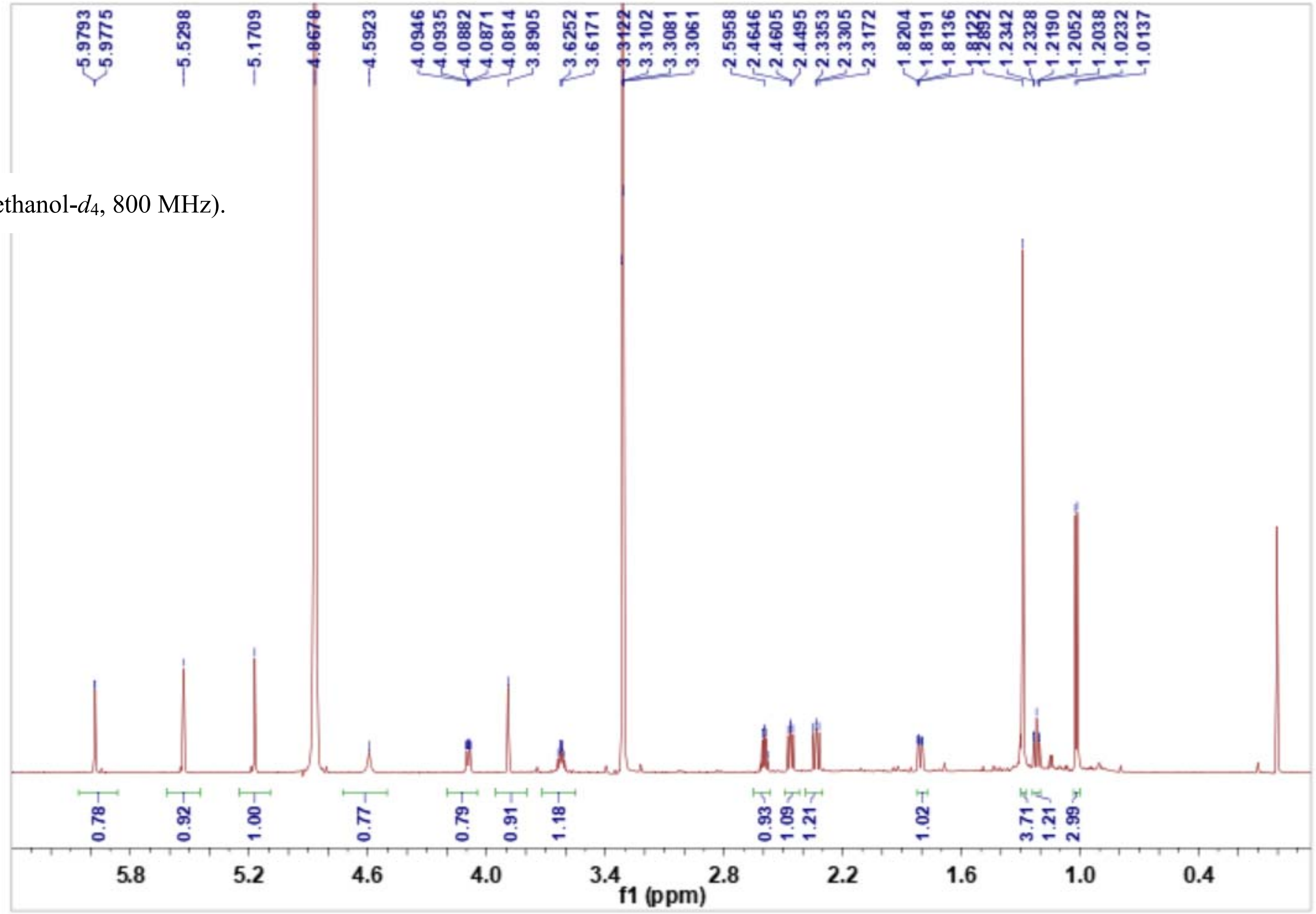

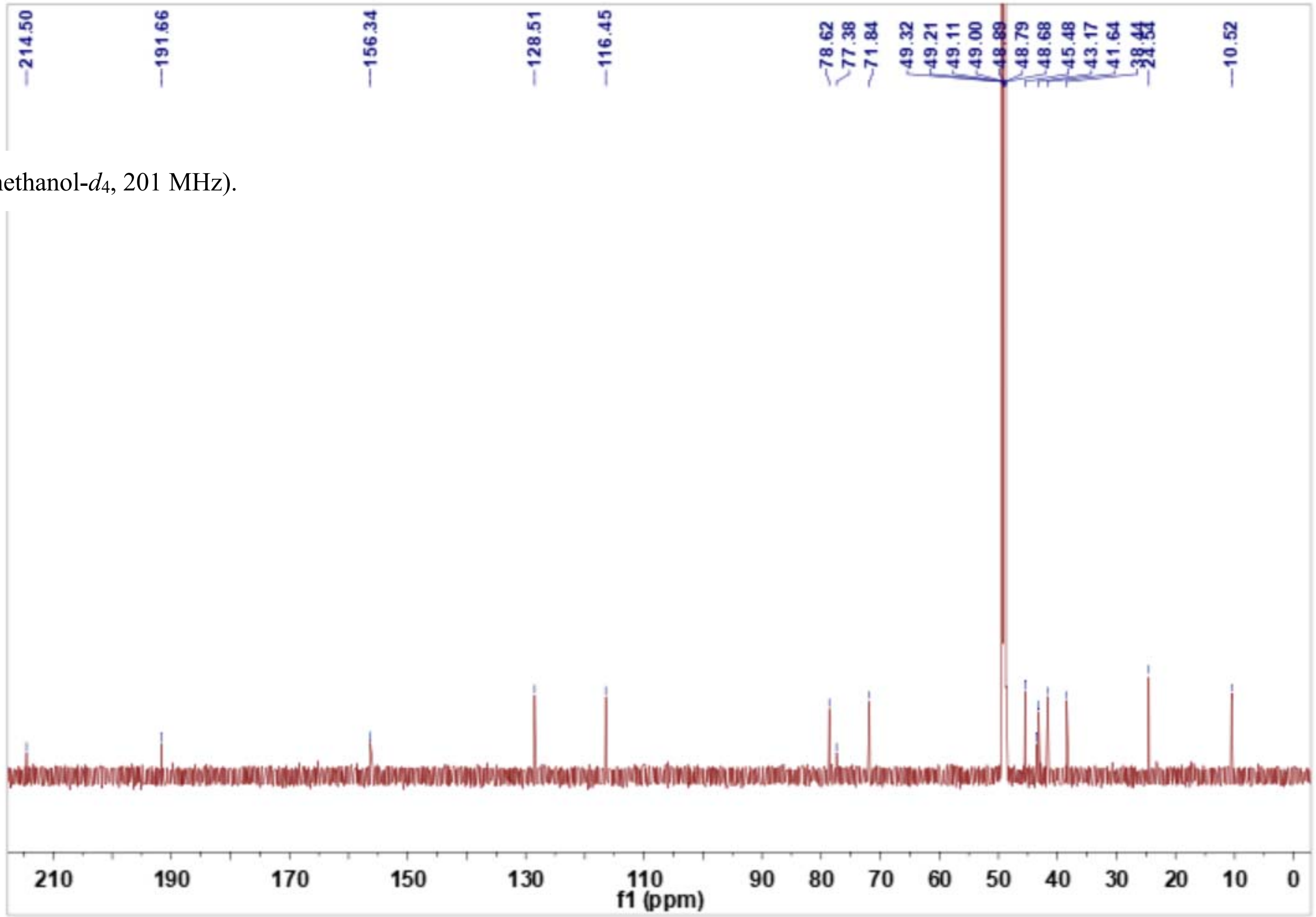

Fig. S60. ^{13}C NMR spectrum of **8** (methanol-d_4, 201 MHz).

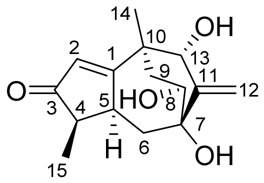

Fig. S61. HSQC spectrum of **8**.

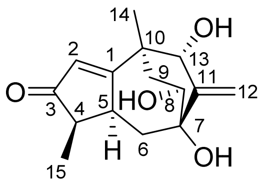

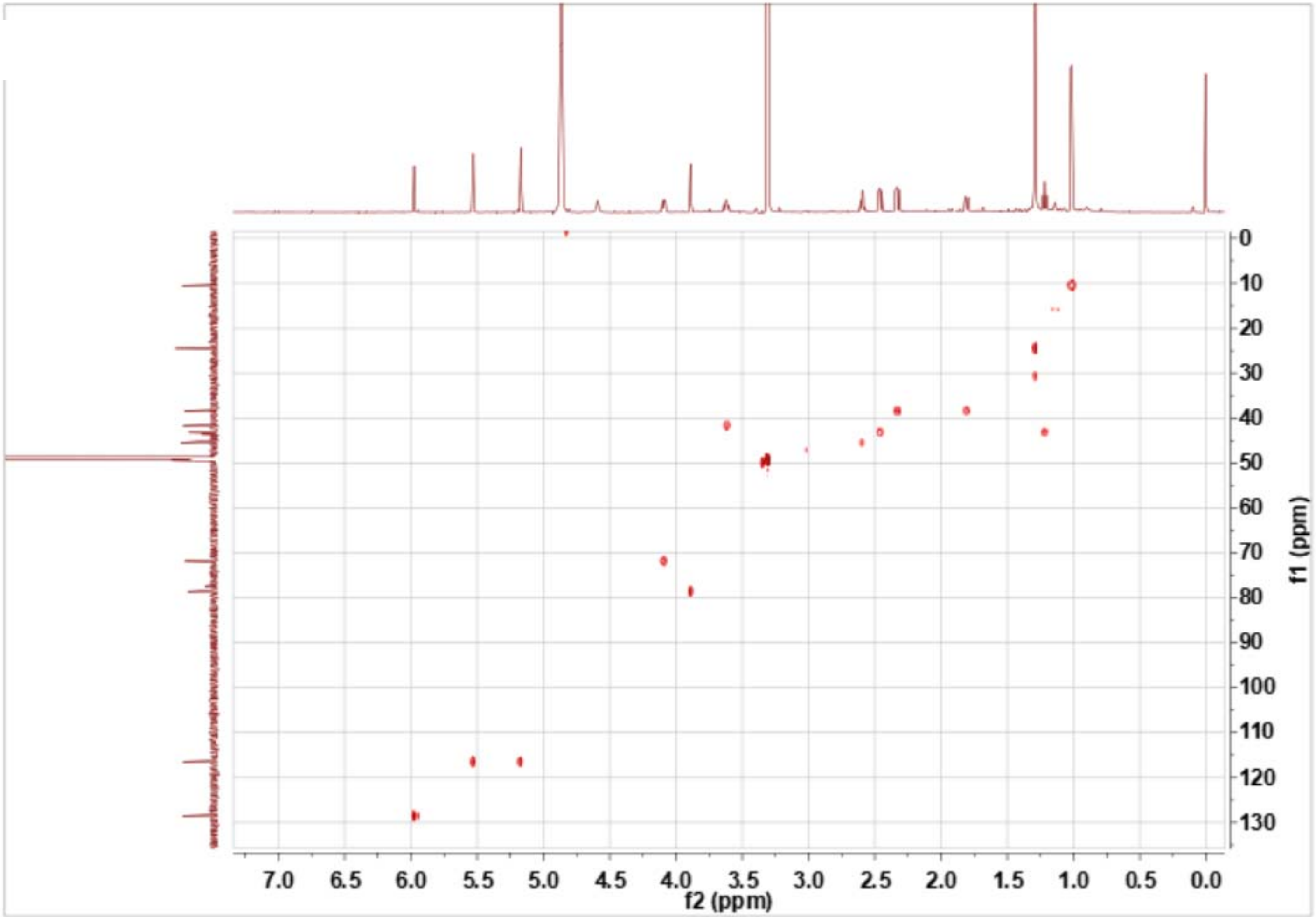

Fig. S62. ^{1}H–^{1}H COSY spectrum of **8**.

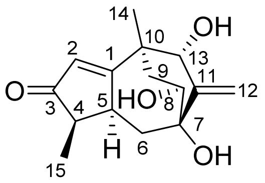

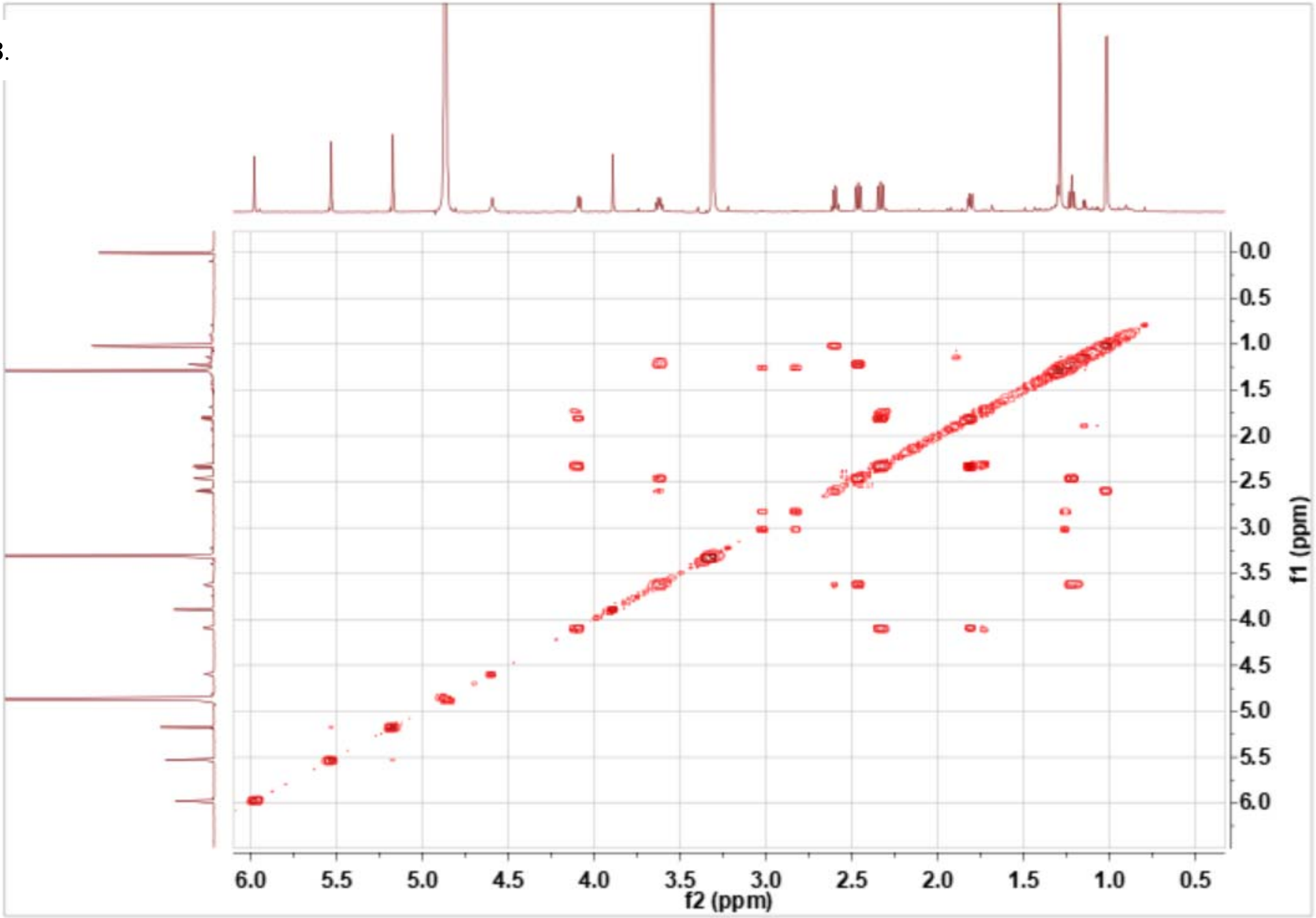

Fig. S63. HMBC spectrum of **8**.

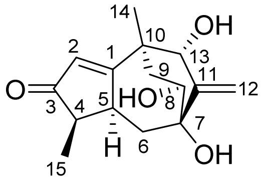

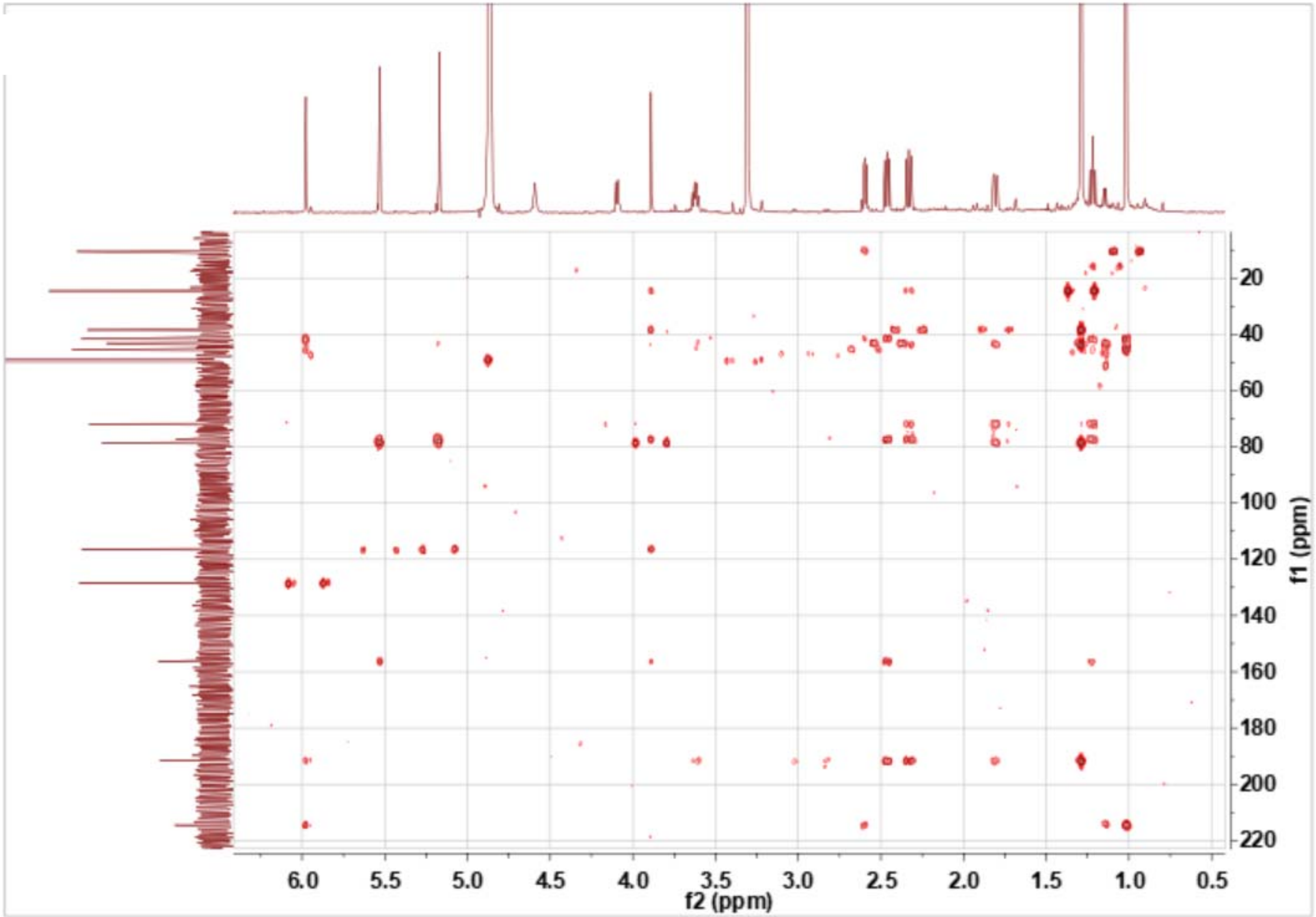

Fig. S64. ROESY spectrum of **8**.

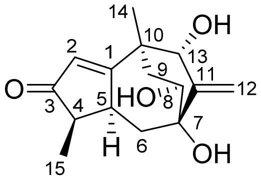

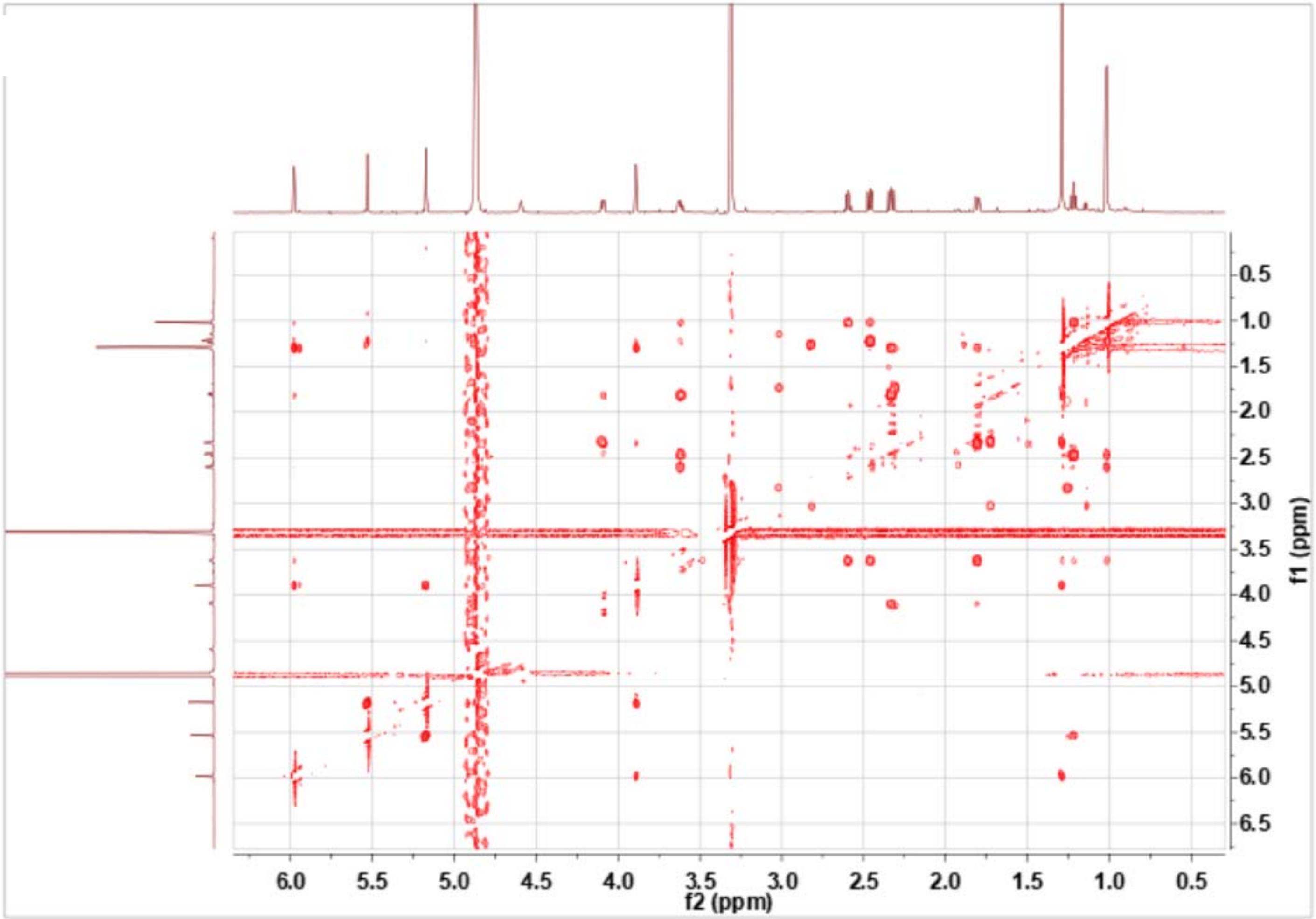

Fig. S65. HREIMS spectrum of **8**.

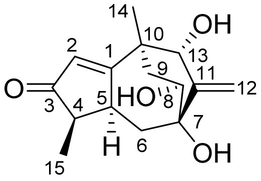

Page 1

Single Mass Analysis (displaying only valid results)

Tolerance = 10.0 PPM / DBE: min = -1.5, max = 50.0
Selected filters: None

HRESIMS → HREIMS

Monoisotopic Mass, Odd and Even Electron Ions
10 formula(e) evaluated with 1 results within limits (up to 50 closest results for each mass)
Elements Used:
C: 0-200 H: 0-400 O: 3-5

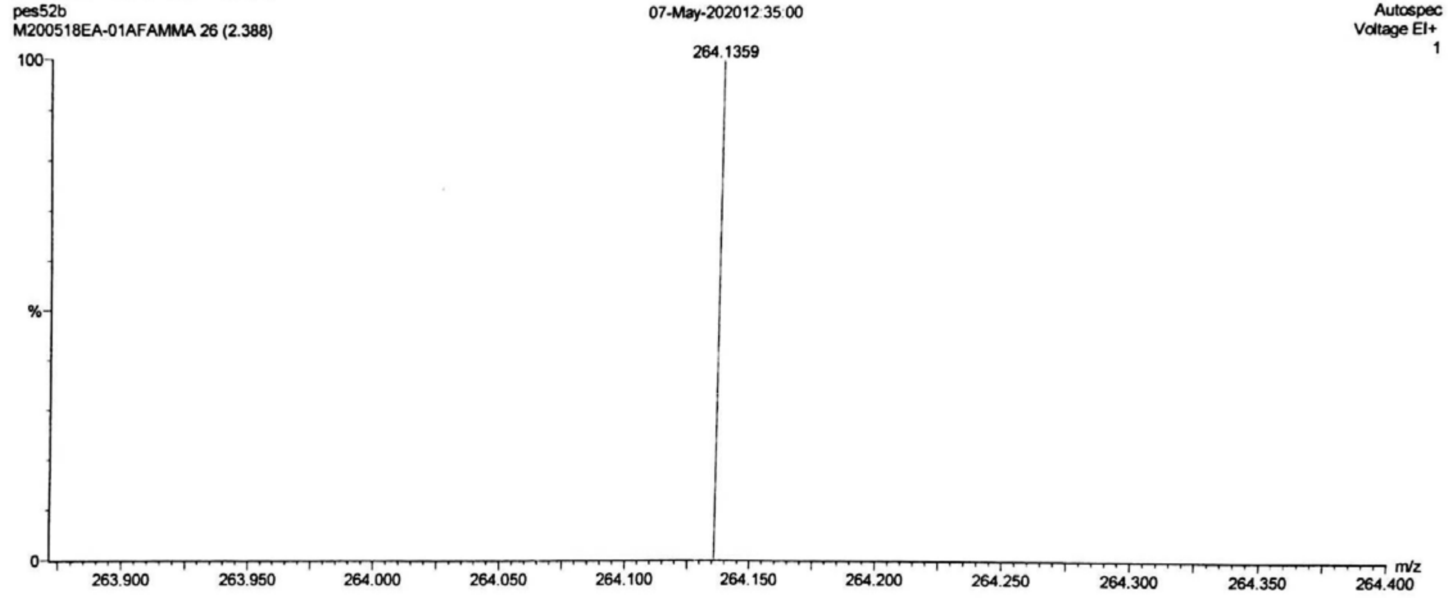

Minimum:				-1.5		
Maximum:		500.0	10.0	50.0		
Mass	Calc. Mass	mDa	PPM	DBE	i-FIT	Formula
264.1359	264.1362	-0.3	-1.1	6.0	5546026.0	C15 H20 O4

Fig. S66. ECD spectrum of **8**.

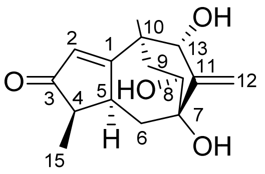

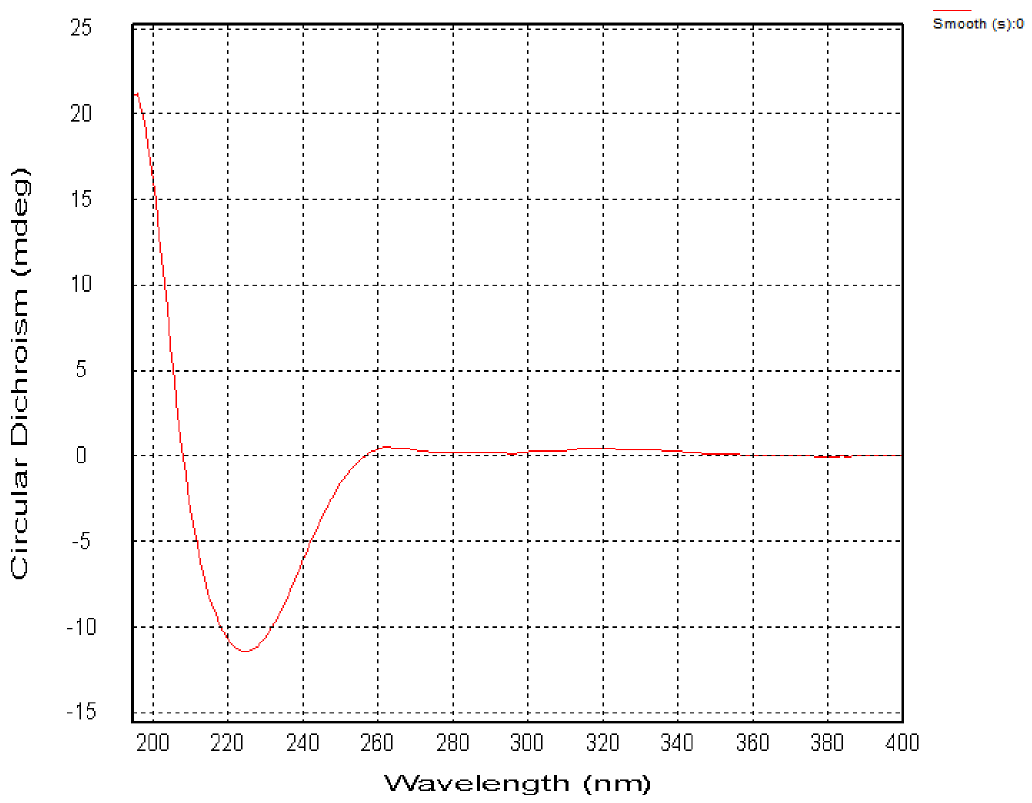

File: PES52B-1mm(195-400nm)20042318.dsx
ProBinaryX
Attributes :
- Time Stamp :Thu Apr 23 16:50:59 2020

- File ID : {1CD44C66-C0B7-440e-A40D-4861E81A244F}
- Is CFR Compliant : false
- Original unaltered data

Remarks:
- User: CD
- Date: 2020/04/23
- Instrument: 0547
- DetectorType: LAAPD
- DichOS Calibration Correction Curve: 0547/2
- HV (CDDC channel): 0 v
- Time per point: 1 s
- Description: Sample 1
- Concentration: 0.1500mg/mLMeOH
- Pathlength: 1 mm
- Temperature: 20℃

Settings:
- HV
- Time-per-point: 1s (25us x 40000)
- SE
- Wavelength: 195nm - 400nm
- Step Size: 1nm
- Bandwidth: 1nm

Fig. S67. ^{1}H NMR spectrum of **9** (methanol-d_4, 500 MHz).

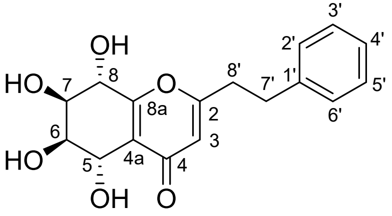

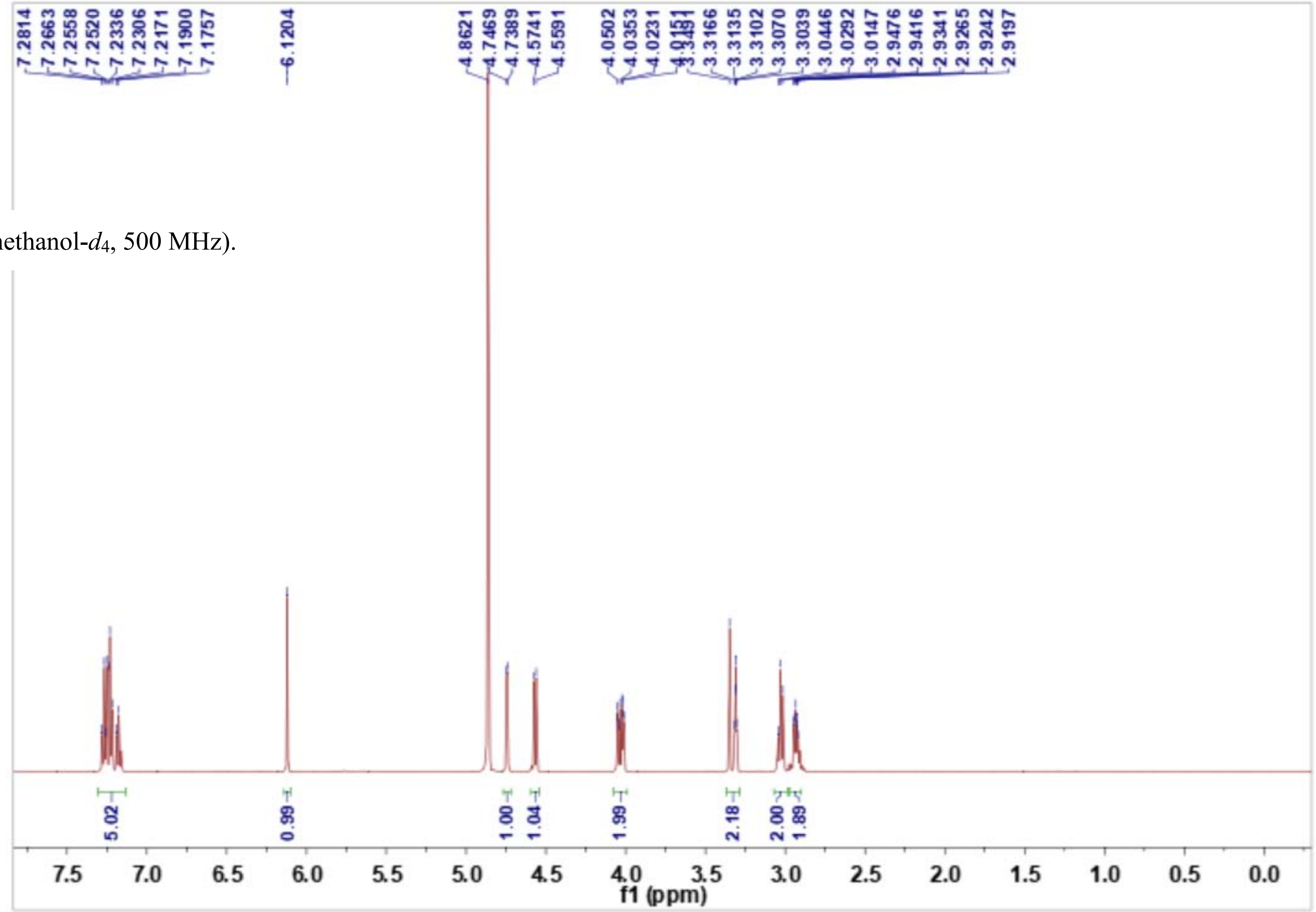

Fig. S68. ^{13}C NMR spectrum of **9** (methanol-d_4, 126 MHz).

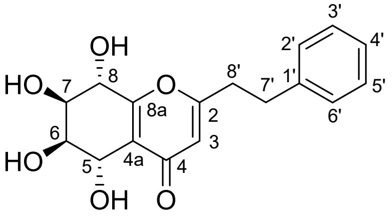

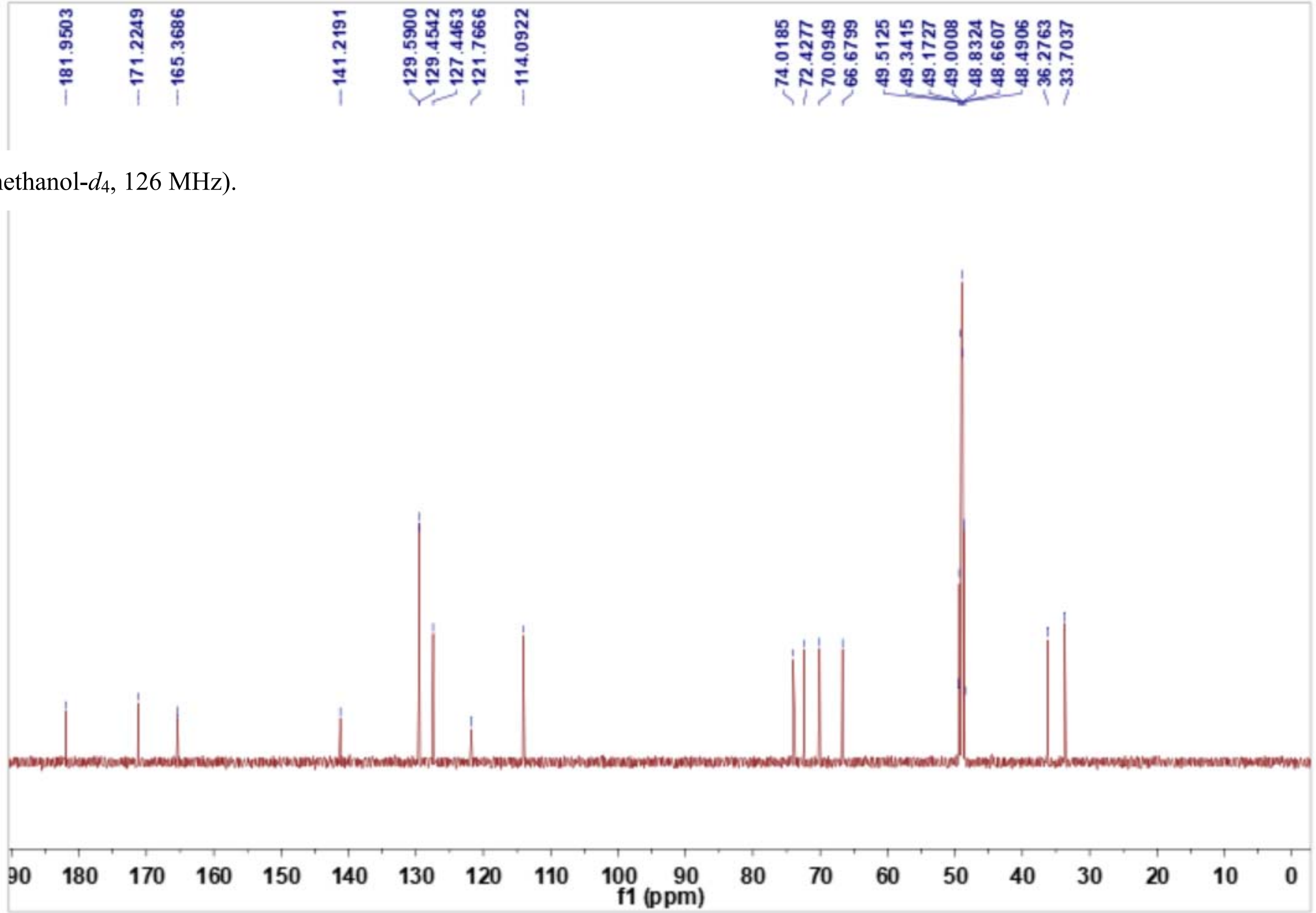

Fig. S69. HSQC spectrum of **9**.

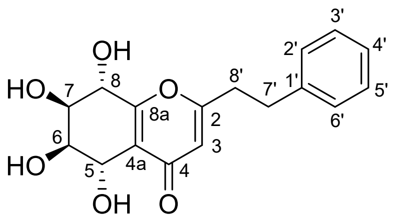

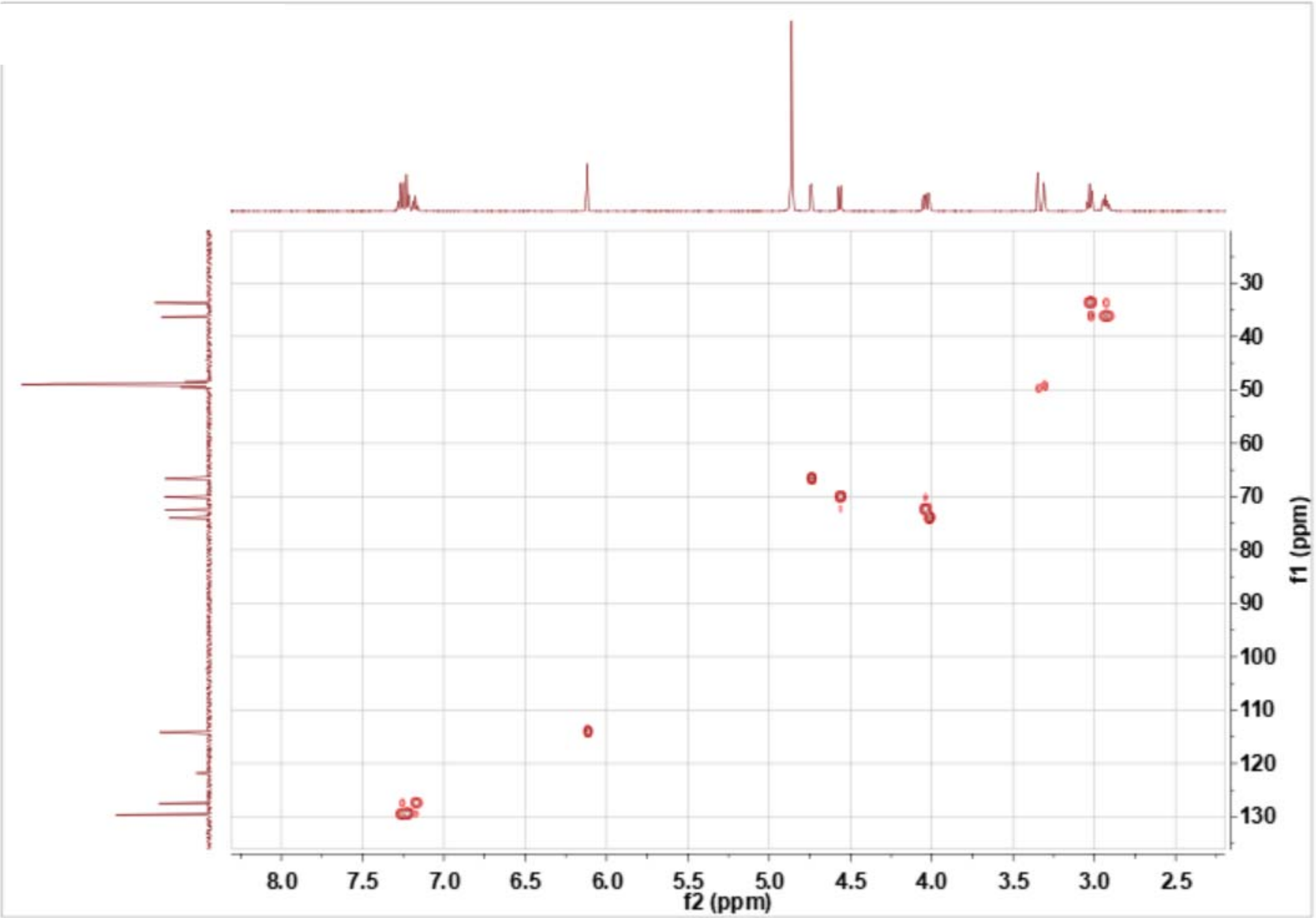

Fig. S70. ^1H–^1H COSY spectrum of **9**.

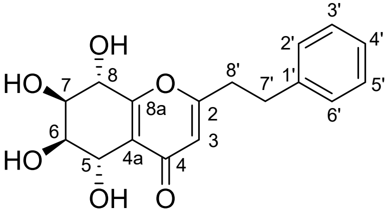

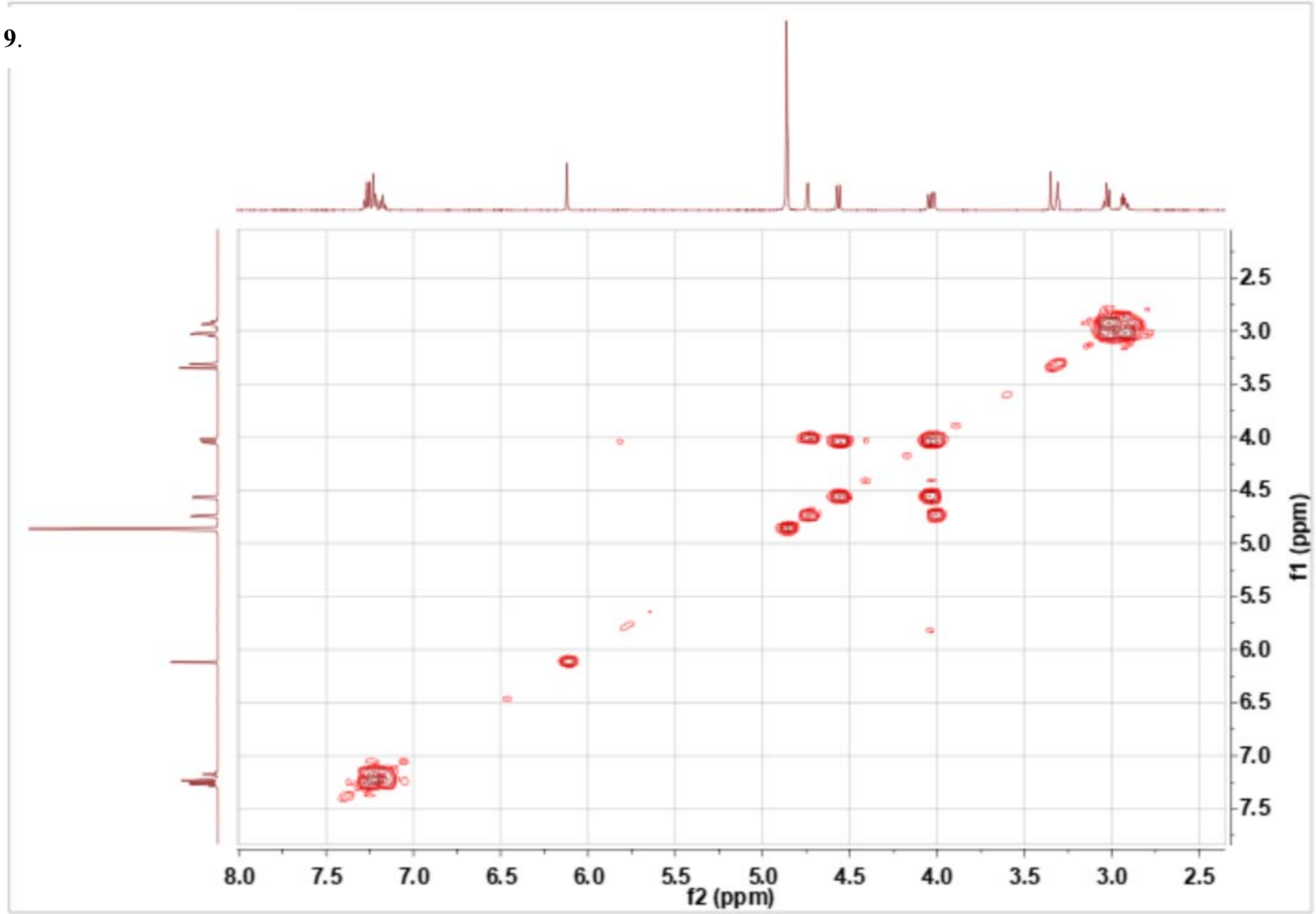

Fig. S71. HMBC spectrum of **9**.

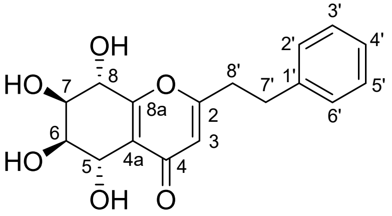

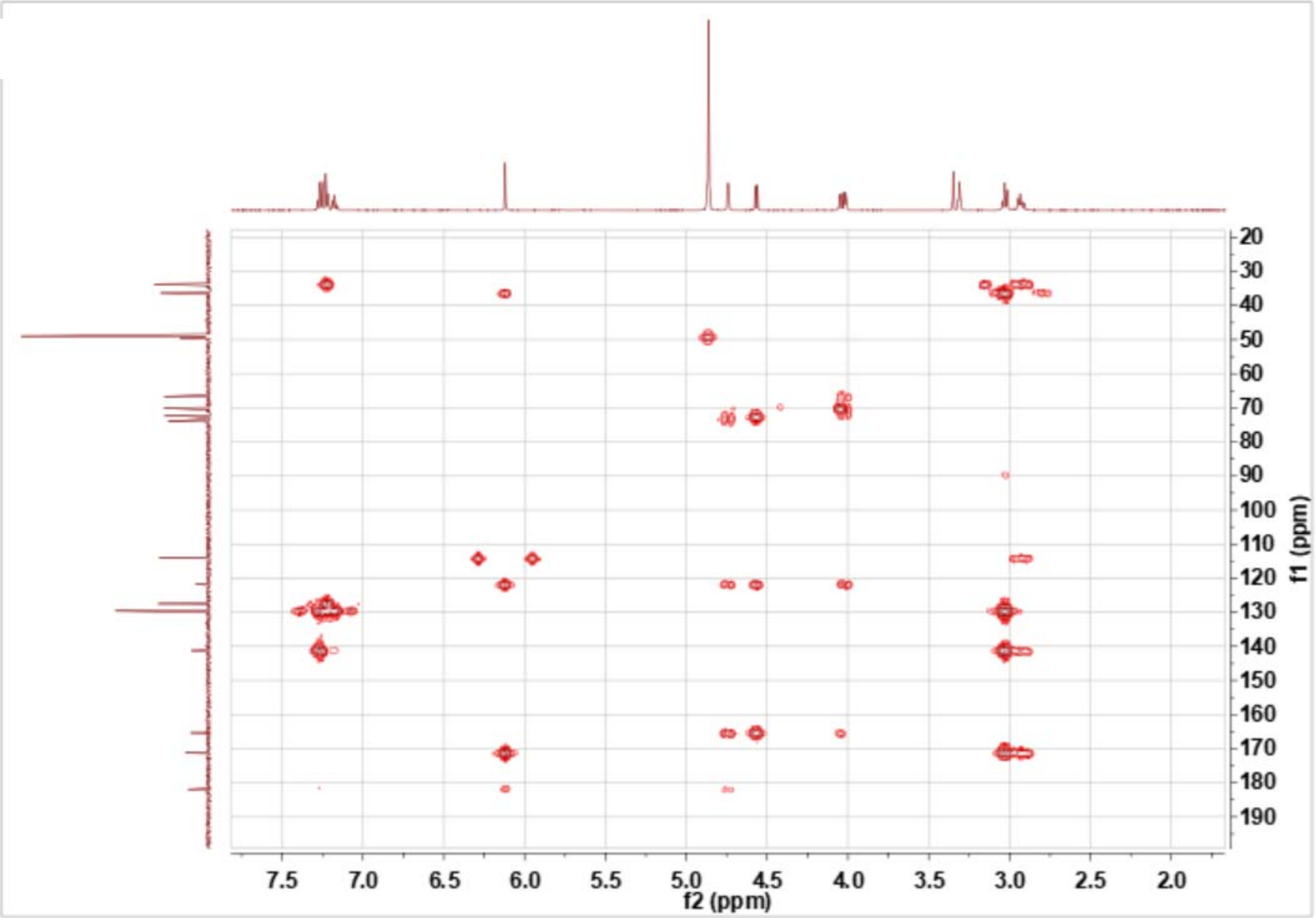

Fig. S72. ROESY spectrum of **9**.

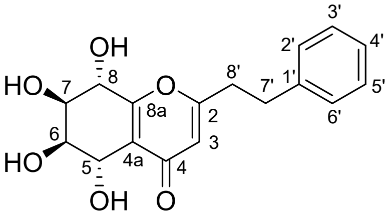

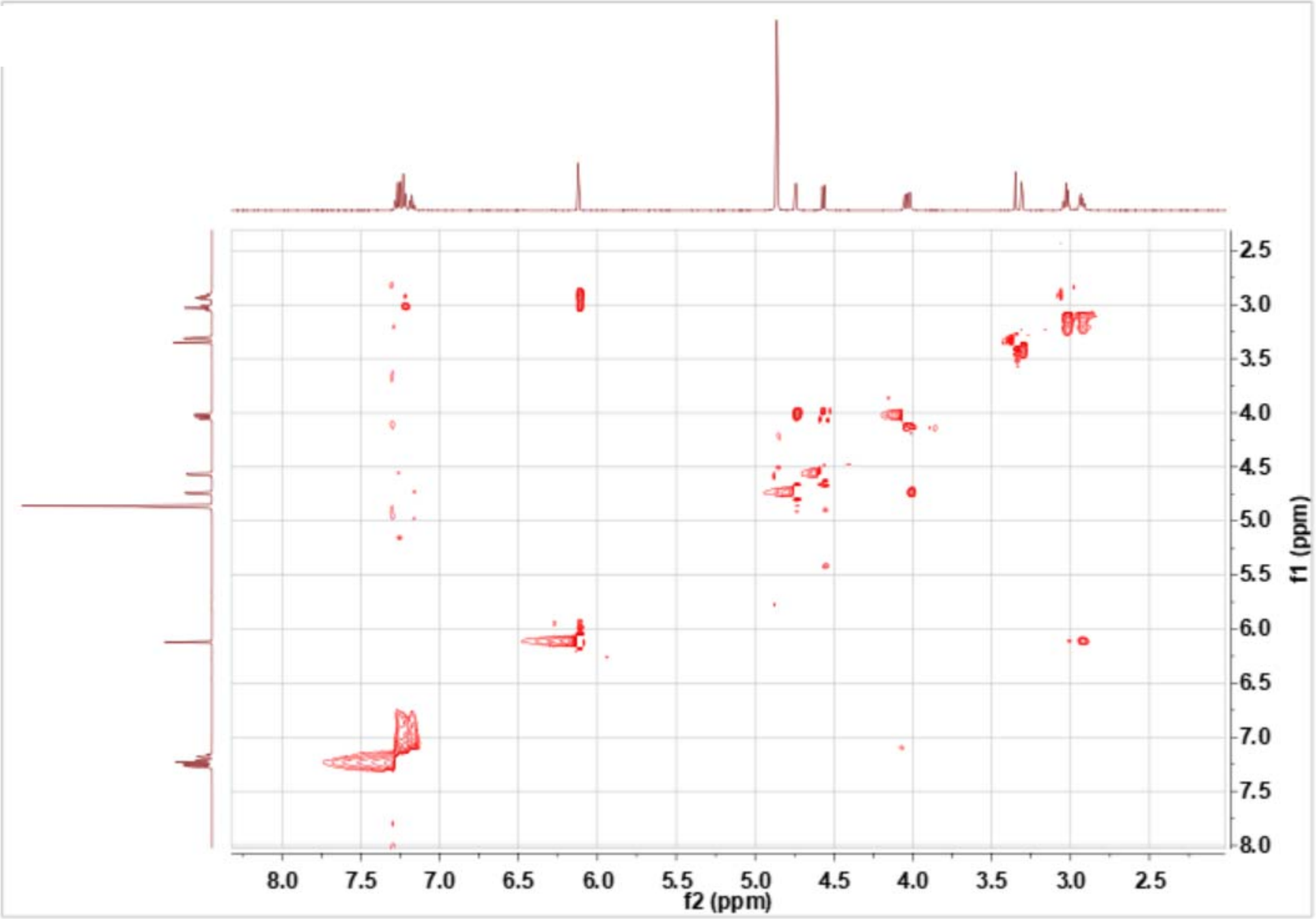

Supplement: Supplementary file 1 — Additional file 1. Chemical structures of known compounds (9–22), key 2D NMR correlations of agarotetrol (9), general experimental procedures, computational methods for ECD of compounds 6–8, and NMR, HRMS, and ECD spectra of compounds 1–8. [file 13659_2022_326_MOESM1_ESM.pdf]
